# Performing statistical analyses on quantitative data in Taverna workflows: An example using R and maxdBrowse to identify differentially-expressed genes from microarray data

**DOI:** 10.1186/1471-2105-9-334

**Published:** 2008-08-07

**Authors:** Peter Li, Juan I Castrillo, Giles Velarde, Ingo Wassink, Stian Soiland-Reyes, Stuart Owen, David Withers, Tom Oinn, Matthew R Pocock, Carole A Goble, Stephen G Oliver, Douglas B Kell

**Affiliations:** 1 Manchester Centre for Integrative Systems Biology and School of Chemistry, Manchester Interdisciplinary Biocentre, University of Manchester, 131 Princess St, Manchester, M1 7DN, UK; 2 Department of Biochemistry, Sanger Building, University of Cambridge, 80 Tennis Court Road, Cambridge, CB2 1GA, UK; 3 Human Media Interaction group, Electrical Engineering, Mathematics and Computer Science, University of Twente, Drienerlolaan 5, 7500 AE, Enschede, The Netherlands; 4 School of Computer Science, Kilburn Building, University of Manchester, Oxford Road, Manchester, M13 9PL, UK; 5 EMBL European Bioinformatics Institute, Hinxton, Cambridge, CB10 1SD, UK; 6 School of Computing Science, University of Newcastle, NE1 7RU, UK

## Abstract

**Background:**

There has been a dramatic increase in the amount of quantitative data derived from the measurement of changes at different levels of biological complexity during the post-genomic era. However, there are a number of issues associated with the use of computational tools employed for the analysis of such data. For example, computational tools such as R and MATLAB require prior knowledge of their programming languages in order to implement statistical analyses on data. Combining two or more tools in an analysis may also be problematic since data may have to be manually copied and pasted between separate user interfaces for each tool. Furthermore, this transfer of data may require a reconciliation step in order for there to be interoperability between computational tools.

**Results:**

Developments in the Taverna workflow system have enabled pipelines to be constructed and enacted for generic and *ad hoc *analyses of quantitative data. Here, we present an example of such a workflow involving the statistical identification of differentially-expressed genes from microarray data followed by the annotation of their relationships to cellular processes. This workflow makes use of customised maxdBrowse web services, a system that allows Taverna to query and retrieve gene expression data from the maxdLoad2 microarray database. These data are then analysed by R to identify differentially-expressed genes using the Taverna RShell processor which has been developed for invoking this tool when it has been deployed as a service using the RServe library. In addition, the workflow uses Beanshell scripts to reconcile mismatches of data between services as well as to implement a form of user interaction for selecting subsets of microarray data for analysis as part of the workflow execution. A new plugin system in the Taverna software architecture is demonstrated by the use of renderers for displaying PDF files and CSV formatted data within the Taverna workbench.

**Conclusion:**

Taverna can be used by data analysis experts as a generic tool for composing *ad hoc *analyses of quantitative data by combining the use of scripts written in the R programming language with tools exposed as services in workflows. When these workflows are shared with colleagues and the wider scientific community, they provide an approach for other scientists wanting to use tools such as R without having to learn the corresponding programming language to analyse their own data.

## Background

The advent of the post-genomic era in biology has led to a dramatic increase in the amount of multi-dimensional, quantitative data that must be analysed by the bioinformatician. This is especially true in the case of genome-scale analyses of the transcriptome, proteome and metabolome, particularly when such measurements have been made in parallel using high throughput technologies involving microarrays and mass spectrometry techniques [1,2]. Analyses of these data rely on the performance of *in silico *experiments, involving the inductive detection of patterns in the data to which some phenotypic significance can be attributed [3]. Such analyses usually rely on statistical testing and linking the results of these tests with information stored in biological databases to summarise and develop conclusions. For example, the analysis of gene expression data generated from microarray experiments consists of a number of steps. The process begins with the normalization and standardization of transcript data, followed by statistical evaluation, and finally, interpretation of the statistical results via the annotation of genes with information relating to their biological function [4].

There are a number of issues associated with the use of computational tools in the analysis of quantitative data. Firstly, learning how to use such tools for statistical analyses can require significant time and effort. This is especially true for mathematical tools such as MATLAB [5] and R [6] which require prior knowledge of their programming languages and the functions within them in order to implement statistical algorithms. Secondly, there is the overhead of transferring data between computational resources during each step of a data analysis pipeline which is made more difficult due to the inconsistent nature of the user interface to the tools. For example, a user may access R from the command line whilst the querying of online sequence databases is made through the use of a web browser. Piping the output of one resource to another will therefore require intermediate staging of the data so that they may be passed manually amongst multiple tools [7]. Thirdly, the interoperability of computational tools can be awkward due to the heterogeneity of data in bioinformatics. The output data provided by a database service may be incompatible as input to the next analysis service both in terms of its structure and its semantics. In these cases, data have to be reconciled by a transformation step in order for them to be consumable by the next service.

*In silico *experiments on bioinformatics data may be realised as workflows consisting of a pre-defined series of tasks that are related to one another by the flow of data between them. Such workflows can be constructed and enacted using applications such as Kepler [8], Triana [9] and Pegasus [10] that automatically direct the flow of data between the information repositories and computational tools responsible for performing the tasks within an *in silico *experiment. These workflow systems enable the use of distributed resources which have been deployed using web services, a distributed computing architecture that uses existing Internet communication and data exchange standards to support interoperable application-to-application interaction over a network [11]. Web service-enabled resources provide a web-based application programming interface (API) that is published in a machine-processable format such as Web Services Description Language (WSDL) [12]. Interaction of client applications with the web service is independent of the computing platform used to host the service resource. Other systems interact with the web service in a manner prescribed by its interface using messages which may be enclosed in a SOAP envelope and are typically conveyed on the web in the form of XML.

The ^my^Grid project has developed a workflow system called Taverna [13] which is capable of invoking several types of local and online tools that can perform the various tasks of a constructed workflow [14]. Different processor implementations are used to invoke applications depending on the type of invocation mechanism including web services which are described in WSDL documents as well as those deployed using the Soaplab [15] and BioMoby [16] frameworks. Workflows consisting of these and other types of processors are composed in the Scufl workflow language using the Taverna workbench, typically by an expert user of analysis and data services [14]. In this paper, we report on how the Taverna workflow system can be used for the statistical analysis of quantitative, post-genomic data. Using an example from the transcriptomics domain, we show a workflow which retrieves data using customised maxdBrowse web services from the maxdLoad2 microarray database [17]. This workflow then performs statistical analysis on the gene expression data using R to generate a list of differentially-expressed genes which is followed by the annotation of the genes with information stored in biological databases. Furthermore, we show how extra functionality can be incorporated into Taverna using a plugin mechanism that has been developed into its new software architecture, thereby enabling it to be tailored for use in different scientific domains including transcriptomics.

## Implementation

### Microarray data analysis workflow

**Figure 1:**
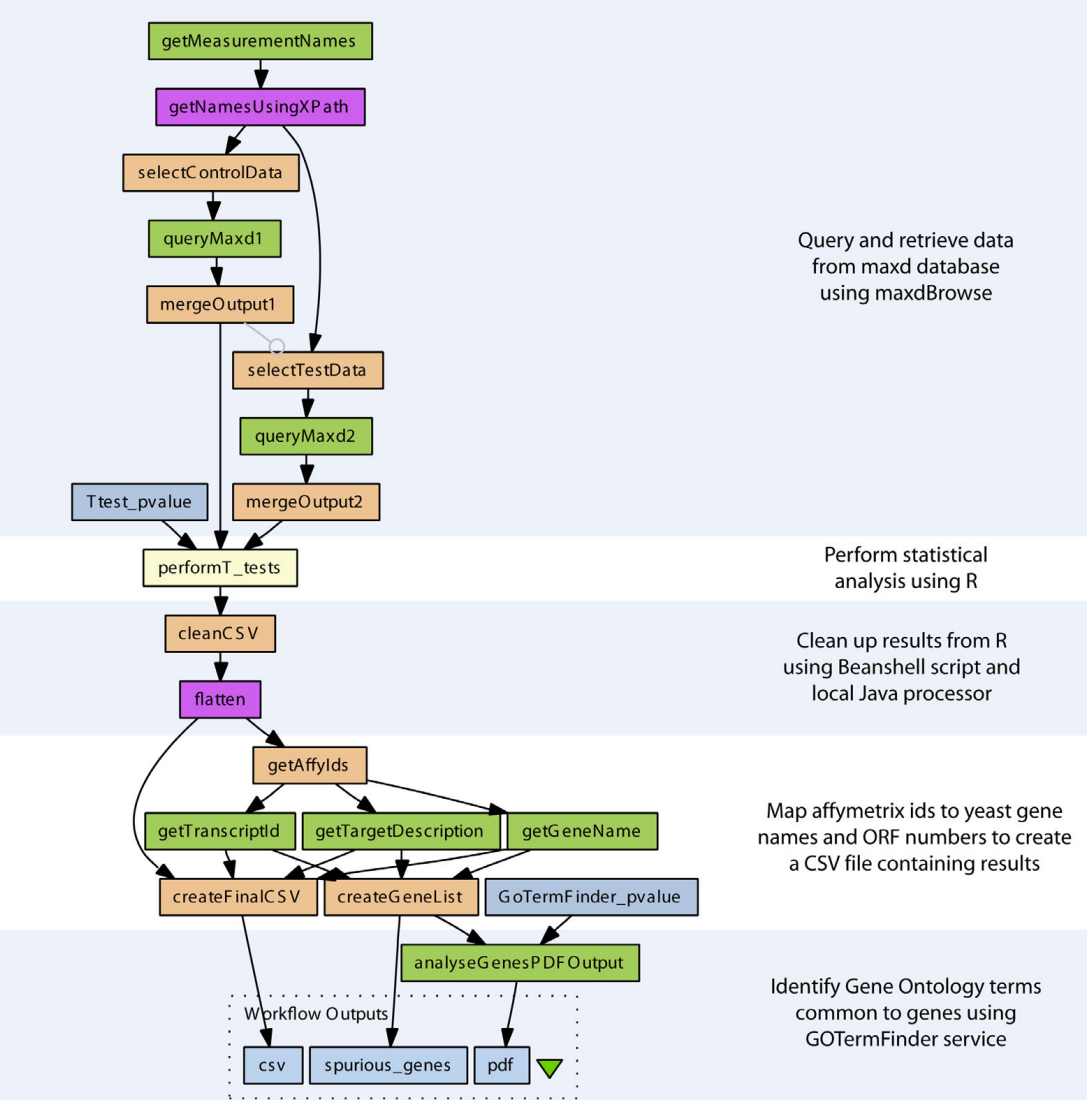
**A diagram of the microarray data analysis workflow**. Data is retrieved from the maxdLoad2 database via the maxdBrowse web service based on user selection criteria implemented using Beanshell scripts. These data are merged and then analysed by R to identify differentially-expressed genes. These genes are then annotated with common terms from the Gene Ontology using the GOTermFinder tool.

The Taverna workflow system is a Java application which is composed of a number of software components including an enactment engine for the execution of workflows. In addition, Taverna provides a workbench which acts as a graphical user interface for the construction of data analysis pipelines and this was used to compose the microarray data analysis workflow shown in Figure 1 (see Additional file 1: workflow.xml). The purpose of this workflow was to discover differentially-expressed genes by *t*-test analyses between two sets of microarray data followed by the identification of common terms from the Gene Ontology (GO) associated with these genes [1,18] (Fig. 1). These terms were identified from the biological process, cellular component and molecular function sub-ontologies of the GO using a web service wrapping of the GOTermFinder tool [19] (Fig. 1).

### Web service access to maxdBrowse

**Figure 2:**
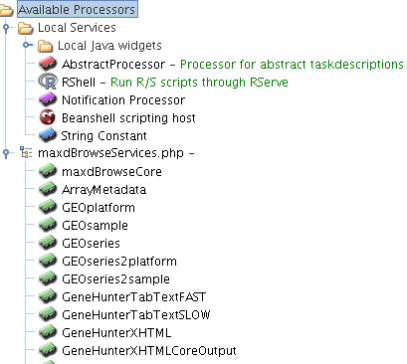
**A screenshot of the Taverna service palette**. The palette shows the RShell processor and the service operations available from the maxdBrowse web service interface to the maxdLoad2 database.

Prior to the analysis of the microarray data, the workflow was also responsible for the selection and retrieval of the gene expression data stored in a maxdLoad2 database, part of the maxd software suite for microarray data storage and visualisation (Figs. 1 and 2) [17]. Microarray data and their associated metadata as defined by MIAME [20] can be loaded into the database using the maxdLoad2 desktop application, whilst data can be queried and retrieved from a web browser using a PHP web application called maxdBrowse [17]. To enable data in maxdLoad2 to be accessed from Taverna workflows, maxdBrowse now provides programmatic access to the database using web services [11]. A default set of operations is provided by the maxdBrowse web service, where each operation performs distinct queries providing access to different metadata about the microarray experiments held within maxdLoad2 as well as the actual gene expression level measurements (Fig. 2). Further operations can be added to maxdBrowse, by a site maintainer, for constructing new queries and customising output formats via a plugin interface. WSDL documents describing the operations for each web service are available and can be imported into the service palette in the Taverna workbench from where they can be selected for inclusion into workflows (Fig. 2).

### Use of Beanshell scripts for reconciling mismatches in data interoperability between services and implementing user interaction during workflow execution

It is often necessary to transform data during their transfer between services within workflows due to incompatible mismatches in their syntactic format. In such cases, manual intervention is required to convert the data into the correct form prior to their consumption by a service in order for the workflow to enact successfully. To this end, the ability to execute scripts written in the Beanshell programming language [21], an interpreted form of Java, was incorporated into a Taverna processor [22]. Beanshell scripts can be written to provide a mechanism of transforming data so that the data are consumable to services within a workflow. Within the example microarray data analysis workflow, Beanshell scripts have been used to merge multiple sets of microarray data retrieved from the maxdLoad2 database prior to their analysis using R. A Beanshell script was also used to generate one of the final outputs of the workflow which was in the form of a text file of comma-separated values (CSV) containing a list of the differentially-expressed genes identified from *t*-tests combined with functional information obtained from public databases (Fig. 1).

An *ad hoc*, albeit basic, form of user interaction can also be implemented using Beanshell scripts through the use of Java Swing classes as part of a workflow enactment [23]. This was used by the example workflow for the multiple selection of two sets of microarray data for *t*-test analysis by R (Fig. 1).

### Invocation of R from Taverna workflows

**Figure 3:**
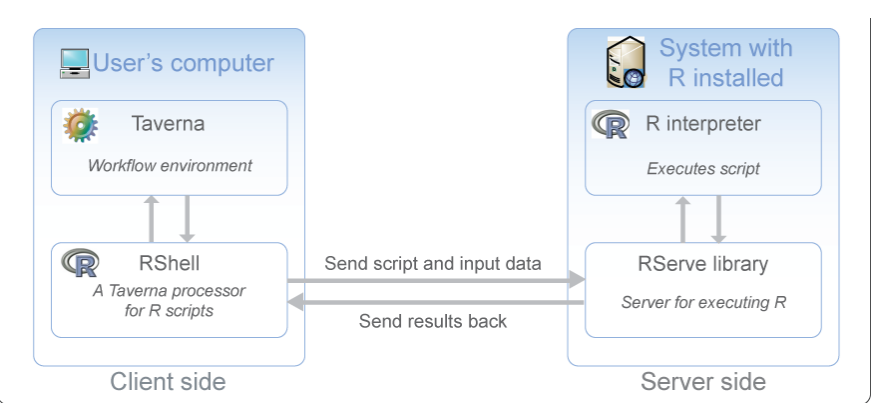
A schematic diagram showing the relationship between Taverna, the RShell processor, RServe and the R tool.

**Figure 4:**
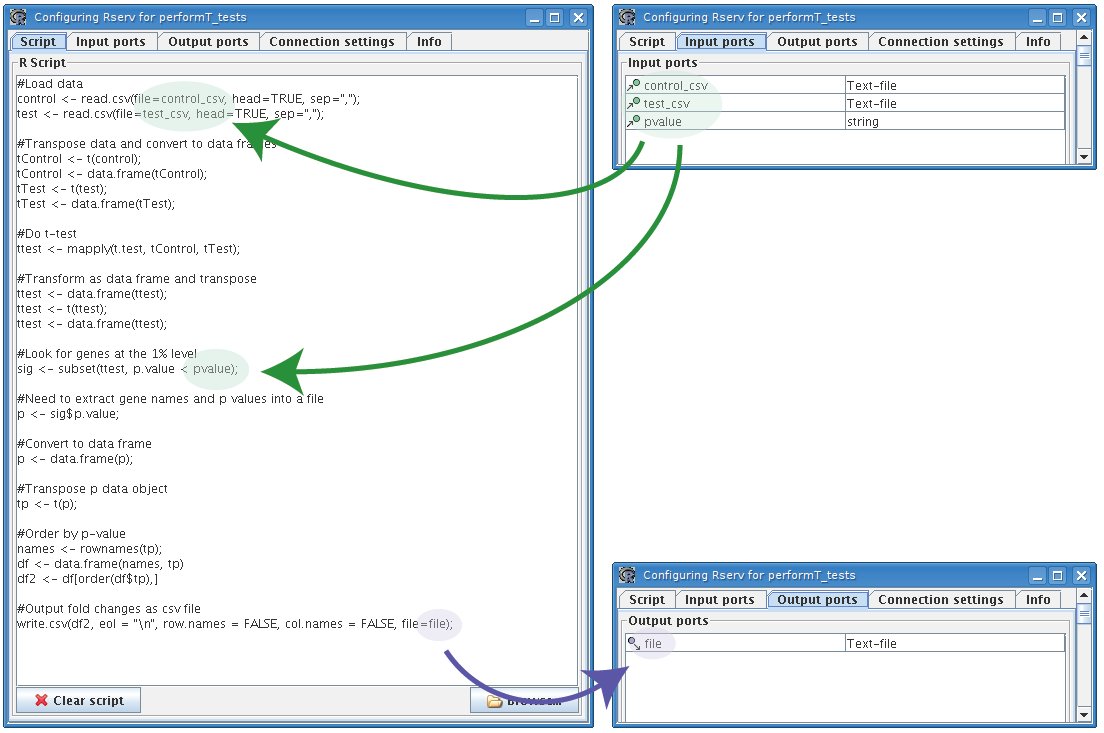
**Relationship between input and output ports with variables in R scripts**. All data items required for analysis and parameterisation are passed into the R script by declaring them as variables named after the input ports. Three input variables have been defined for the RShell processor in example workflow, the 2 sets of microarray data, 'control_csv' and 'test_csv', undergoing *t*-test analysis and 'pvalue' which defines the threshold used for the *t*-tests. The results of the R analysis can be passed out into the remainder of the workflow by declaring them as variables named after the output ports. The example RShell processor in the workflow contains only one output, a CSV file containing the differentially-expressed genes identified by the *t*-tests.

The next step in the workflow is the statistical analysis of the two sets of gene expression data once they have been selected and retrieved from maxdLoad2. A Taverna processor called RShell has been developed to invoke scripts in the R computing environment, and was used to perform the statistical calculations for the *t*-test analyses [6] (Figs. 1 and 2). This processor acts as a client to R when it has been deployed as a TCP/IP server using the RServe library [24], relaying the script and its inputs to be executed in the R environment (Fig. 3). The script contains statements in the R programming language to implement the statistical calculations. In the example workflow, the RShell processor invokes the R server with a script to perform a *t*-test for every gene expression level, identifying those genes which are differentially-expressed from the data retrieved from maxdLoad2 (Fig. 1). The location of the R server to be used for analysis is defined by a hostname and port number declared when configuring the processor. Data are fed to the RShell processor via its input ports and are made available to the R script as variables named after the ports (Fig. 4). In a similar fashion, the results generated after the execution of the R script are available from the RShell processor via its output ports by reading the last value assigned to the variable named after them (Fig. 4). In addition, graphs generated by R can also be returned to Taverna in the form of images as outputs by the workflow.

### Use of plugins for extending Taverna functionality

Both the Beanshell and RShell processors provide Taverna with access to generic tools for analysing different types of quantitative data. This is accomplished by these two processors without the need to develop and deploy web services, thereby enabling rapid prototyping without a web services infrastructure. Taverna can also be tailored for use in specific scientific domains by extending its functionality with additional source code through the use of software plugins. A mechanism for plugins has been incorporated into Taverna following a refactoring of its software architecture effected by the development of a tool called Raven. Originally inspired by Maven [25], Raven enables Taverna to be run according to information about the JAR libraries that Taverna components are dependent upon which reside in an XML file called the Project Object Model (POM).

An organisation can deploy a plugin site for Taverna using two types of XML files. Firstly, an XML file is required which lists the available plugins. Secondly, each listed plugin requires an XML file containing information about its version, functionality and from where the POM file for the plugin can be downloaded. Based on the information in this POM, Raven will then download and install the associated and dependent JAR library files for the plugin for use in Taverna workflows. Further information about the development and deployment of plugins is available online in the developers guide on the Taverna web site .

**Figure 5:**
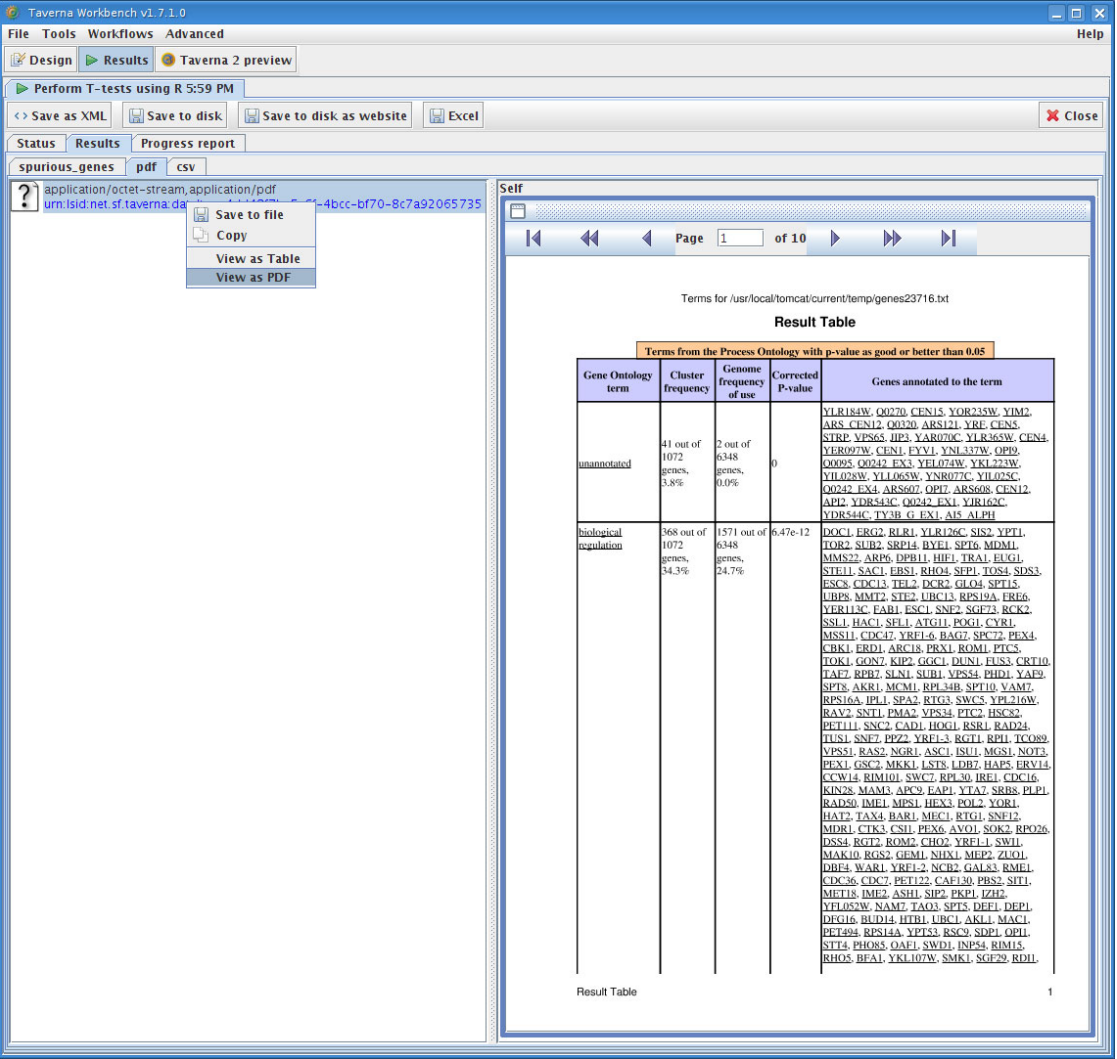
**A screenshot showing the PDF report generated by the GoTermFinder web service during the execution of the microarray data analysis workflow**. The PDF report is displayed using the PDFRenderer plugin for Taverna by right-clicking on the PDF file object and selecting View as PDF.

There are interfaces specified in the API of Taverna [26] for extending different aspects of its workflow system with plugins including renderers for visualising different types of data which result from workflow outputs, and processors for invoking services with specialised programming interfaces. Plugins and their dependencies are shared with the scientific community by their deployment to Maven repositories which can be made available on a web server. Plugins are installed in the Taverna workbench through its Plugin Manager by providing a URL which points to the XML file that lists the available plugins; those required by the user can then be selected for installation and use. In the microarray data analysis workflow, plugins were developed for displaying PDF documents (Fig. 5) as well as for visualising quantitative data when formatted as CSV as a table. These two renderer plugins can be downloaded and installed for use in Taverna 1.7.1 from  using the Plugin manager on the Taverna workbench.

## Results

The example workflow shown in Figure 1 was evaluated using microarray data originating from a study into the effects of growth rate on the transcriptome of the yeast, *Saccharomyces cerevisiae*, when grown under different nutrient-limiting conditions (see Additional file 1: workflow.xml) [1]. The data from this study were normalized by GC Robust Multi-array Average background adjustment and then stored in the maxdLoad2 database [17]. The enactment of the workflow started with the selection and retrieval of data from the microarray database using the maxdBrowse web service interface and Beanshell scripts. This was followed by the invocation of R with a script to perform a series of *t*-tests to identify the genes that are differentially-expressed between growth rates and culture conditions (Figs. 1 and 4). Common terms from the GO associated with these genes were then identified using the GOTermFinder web service. The GOTermFinder service returns PDF reports of these common GO terms which were browsed using the Taverna workbench with the PDFRenderer plugin (Fig. 5).

Using the example workflow, the gene expression levels of yeast cultures growing under carbon limitation were selected for comparison between two different growth rates; those genes whose expression differs at 0.07 h^-1^compared with 0.1 h^-1^. When the genes identified from *t*-test analyses at a p-value of less than 0.05 were subjected to GO analysis by the GOTermFinder tool with a p-value at the 1% level, only broad GO categories relating to the regulation of metabolic processes were identified (see Additional file 2: carbon-ttest-0_01.zip). However, when the same set of genes was analysed by the GO tool with a less stringent threshold (p-value < 0.05), more GO terms appeared relevant including those associated with gene expression (see Additional file 2: carbon-ttest-0_01.zip). The corresponding analysis of GO molecular functions for the same set of genes showed that some were involved in transcription regulation as well as encoding for a number of protein kinases.

The same analysis was applied to *S. cerevisiae *cultures growing at rates of 0.07 h^-1 ^and at 0.10 h^-1 ^under nitrogen limitation by selecting the appropriate data stored in the maxdLoad2 database during the enactment of the example workflow. A different pattern from that of the carbon limitation was observed since *t*-test analysis (p-value < 0.05) and GO analyses (p-value < 0.05) found a higher number of genes whose products are associated with the metabolism of nitrogen compounds (see Additional file 3: nitrogen-ttest-0_05.zip). The most over-represented GO molecular function categories for this gene set corresponded to a variety of catalytic activities such as oxidoreductases, transmembrane transporter activities, and transferases.

The full set of results from the above analyses can be downloaded from .

## Discussion

Statistical analyses of quantitative data can be constructed and performed in a generic fashion using the Taverna workflow system. This was demonstrated by the *t*-test identification of differentially-expressed genes from microarray data using the workflow shown in Figure 1. Such generic analyses were made possible by a number of new features present in Taverna and in particular the development of the RShell processor which acts as a client to R when deployed as a service using the RServe library. Standalone web services can offer specific types of statistical analysis such as clustering for data analysis. However, the development of the R processor means that any type of statistical analysis can be performed on data when implemented as a script in the R programming language within a Taverna workflow [6]. This is of particular benefit to the transcriptomics domain since this processor provides Taverna with access to further tools based on R for analysing microarray data such as Bioconductor [27] which was used by Fisher and colleagues for the identification of candidate genes associated with Trypanosomiasis in workflows [28]. A number of tools such as oneChannelGUI [29] and GenePublisher [30] already provide users with access to R and Bioconductor from a graphical user interface. However, the advantage of coupling the R tool with an application like Taverna is that the workflow system provides an interoperability platform for data to be fetched from distributed services for subsequent analysis using R in a workflow. Conversely, the results of an R analysis can be sent for further processing by services downstream in the workflow. The use of distributed data and analysis services in conjunction with R were demonstrated in the example workflow (Fig. 1). Data analysed by R were provided by the maxdLoad2 microarray database using its web service interface provided by maxdBrowse and the results of this were further analysed by the GOTermFinder tool.

The current work on analysing quantitative data extends that by Stevens and coworkers who investigated the implementation of gene annotation pipelines as workflows in a previous version of Taverna [31]. Since then, *ad hoc *functionality can be incorporated into Taverna workflows through the use of a processor which executes scripts written in the Beanshell language Java [21]. Such scripts provide a method for implementing shim services [32] which, in the example workflow, were used to reconcile data mismatches between services (Fig. 1). Beanshell scripts were also used to implement a basic form of user interaction using Java Swing classes to steer workflows during their enactment. In the example workflow, this technique was used to select different pair-wise combinations of classes for *t*-test analysis. This type of interaction is used by the workflow user to direct the workflow to analyse specific subsets of data which are not known at runtime.

Scripts written in the Beanshell and R languages provide generic functionality which can be used as tools to analyse specific types of quantitative data from different scientific domains. In addition, domain-specific functionality can be provided for use in scientific workflows through the plugin mechanism now present in Taverna's software architecture. The ability to incorporate plugins makes it easier for the scientific community to contribute and share functionality in Taverna without the need for their source code to be tightly coupled with the core. In the example workflow (Fig. 1), the plugin mechanism was demonstrated by the implementation of renderers for browsing PDF documents (Fig. 5) and the display of text files containing quantitative data in CSV format as tables. In the context of the current work, an opportunity for developing other plugins for analysing quantitative data might include the ability to invoke analyses in other applications such as MATLAB [5] and Mathematica [33] for those users who prefer these tools to perform their calculations.

An initial investment in time and effort is required to construct data analysis workflows. This was true in the case of the example microarray data analysis workflow since a working knowledge of the R programming language is required to devise the *t*-test analyses, as well as experience of Java programming for writing Beanshell scripts to implement user interaction and shim services. A lack of experience in these two languages can therefore prohibit the construction of complex data analysis workflows by entry-level users of Taverna. However, the fact that workflows can be saved as XML files in the Scufl language allows these analyses to be shared with colleagues and with the wider life sciences community. This is especially pertinent to multi-disciplinary research groups whose expert data analysts use R and other similar tools to develop data analysis protocols, which are then employed by colleagues who are laboratory scientists. This laboratory group of scientists understand the conceptual basis of the analysis performed by the R script but may not have the inclination (or the need) to learn the R programming language in order to understand how such scripts have implemented their analyses. Since it is often the case that there are many more producers of data than there are experts in data analysis within a research group, it is desirable for the laboratory scientists themselves to perform analyses of their own data by re-using R scripts, that may have been incorporated into workflows, written by the data analysis experts. These workflows can therefore be regarded as standard operating procedures providing a best-practice solution for analysing specific types of data. The automation of analyses afforded by Taverna and other workflow software such as Kepler [8] enables laboratory scientists to quickly test hypotheses on their data by guiding them through complex statistical analyses, especially when users can steer and interact with the enactment of a workflow. This enables a user to run multiple analyses with different parameterizations which was required for the analysis of the microarray data in this study in order to extract information about what was biologically significant. This benefit provided by a workflow approach to users has been implemented by providers of commercial microarray data analysis software such as GeneSpring [34].

There are still a number of issues highlighted by the current microarray data analysis study associated with the use of web services and workflows that still need to be addressed. Firstly, there is the problem of securing analysis services such as that provided by an R server used in the example workflow. Such services may have been deployed on powerful compute clusters and so their use by unauthorised users within workflows is undesirable. Using the RServe library, the R server can be configured to allow access for those users providing the appropriate username and password combination. However, this information is embedded within the Scufl file and so it is not suitable to share this file with anyone other than colleagues. Future work on the development of Taverna 2, involving the use of distributed agents to handle the security of services during workflow enactment will address this problem. The sharing of workflows within the life sciences community is also an issue that is currently being addressed by the ^my^Experiment project [35]. It introduces the concept of social networking web sites for workflows and aims to provide a collaborative environment for scientists to safely publish their workflows with supporting documentation, and share them with a wider group of people as well as addressing concerns relating to their attribution, credit and intellectual property on the web.

## Conclusion

Statistical calculations implemented in the R programming language can be combined with the use of other distributed computational tools and databases in Taverna workflows for performing bespoke analyses on post-genomic data. In this fashion, the Taverna workflow system provides a generic tool for the analysis of quantitative data. Together with Beanshell scripts and specially designed plugins, workflows can be written for the analysis of data from different scientific domains by data analysis experts. These workflows act as standard operating procedures which guide users in the analysis of the data they have generated, enabling them to quickly test hypotheses against data.

## Availability and requirements

Taverna (version 1.7.1) and its supporting documentation are available from . Supplementary material including the example workflow, its accompanying documentation, and the results of the analyses described in this paper are available from . The example microarray data analysis workflow is also available from the ^my^Experiment web site: .

## Authors' contributions

PL was responsible for writing the manuscript with the help of DBK. PL also developed the microarray data analysis workflow including the embedded R and Beanshell scripts, and the plugins for displaying the results of the workflow in the Taverna workbench. JIC and SGO interpreted the results from the workflow analyses and revised the manuscript. GV developed the MaxdBrowse interface to the maxdLoad2 database. TO, MRP, SS–R, DW and SO are developers on the Taverna and ^my^Grid projects which are led by CAG. IW and SS–R developed the RShell processor. All of the authors have read and approved the final manuscript.

## Supplementary Material

Additional file 1 Workflow. Click here for file

Additional file 2 Carbon t-test. Click here for file

Additional file 3 Nitrogen t-test. Click here for file
