# Supplementary material for: Performing statistical analyses on quantitative data in Taverna workflows: An example using R and maxdBrowse to identify differentially-expressed genes from microarray data
Source: BMC Bioinformatics. 2008 Aug 7;9:334. doi: 10.1186/1471-2105-9-334 (PMC2528018; doi:10.1186/1471-2105-9-334)
Supplement: Additional file 2 — Carbon t-test. [file 1471-2105-9-334-S2.zip › 0.01ttest/0.01Go/cellcomp.pdf]

Terms for /usr/local/tomcat/current/temp/genes40239.txt

Result Table

Terms from the Component Ontology with p-value as good or better than 0.01				
**Gene Ontology term**	**Cluster frequency**	**Genome frequency of use**	**Corrected P-value**	**Genes annotated to the term**
unannotated	17 out of 353 genes, 4.8%	out of 6348 genes, 0.0%	0	YAR070C, API2, YER097W, CEN4, YLL065W, CEN1, YNL337W, ARS607, Q0242_EX4, Q0095, ARS_CEN12, ARS121, YRF, STRP, JIP3, CEN12, AI5_ALPH
