# Supplementary material for: Performing statistical analyses on quantitative data in Taverna workflows: An example using R and maxdBrowse to identify differentially-expressed genes from microarray data
Source: BMC Bioinformatics. 2008 Aug 7;9:334. doi: 10.1186/1471-2105-9-334 (PMC2528018; doi:10.1186/1471-2105-9-334)
Supplement: Additional file 2 — Carbon t-test. [file 1471-2105-9-334-S2.zip › 0.01ttest/0.05Go/biolproc.pdf]

Result Table

Terms from the Process Ontology with p-value as good or better than 0.05

Gene Ontology term	Cluster frequency	Genome frequency of use	Corrected P-value	Genes annotated to the term
unannotated	17 out of 353 genes, 4.8%	2 out of 6348 genes, 0.0%	0	YAR070C, API2, YER097W, CEN4, YLL065W, CEN1, YNL337W, ARS607, Q0242_EX4, Q0095, ARS_CEN12, ARS121, YRF, STRP, JIP3, CEN12, AI5_ALPH
biological regulation	125 out of 353 genes, 35.4%	1571 out of 6348 genes, 24.7%	0.00174	SNF6, HSC82, ERG2, KAR4, SUB2, GCN1, BYE1, PHO85, RAD24, SPT6, RSR1, PRP45, MMS22, ARP6, TUS1, BFA1, SNF7, HIF1, RGT1, TRA1, YKL107W, TCO89, RPI1, NGR1, RAS2, ISU1, ASC1, ARG5,6, MGS1, NOT3, CEG1, RPS16B, CBF1, PDR3, SFP1, RIM101, CCW14, KIN28, GTR2, MSS116, EST1, SSK2, IRA2, XBP1, POL2, CDC39, SLH1, TAX4, HAT2, RRN5, STE2, MEC1, DIG1, RTG1, YPD1, FRE6, MET4, CTK3, YER113C, SNF2, SOK2, RGR1, TAF14, FYV4, GLC7, ROM2, YRF1-1, CYR1, UAF30, MSS11, GEM1, RGS2, HBS1, DBF4, CBK1, ARC18, YPL017C, PTP2, YRF1-2, WAR1, COS3, NCB2, RME1, PTC5, TRX3, CDC7, YGR111W, APC1, SLF1, SPP1, CAF130, PBS2, HBT1, VID28, PIG1, SAS5, IME2, HAP1, TAH11, DUN1, TOM1, ISW1, TAF7, RPB7, ELA1, YMR1, IZH2, NUT2, TAO3, ARG80, DEF1, SPT10, VAM7, STB4, RPS16A, SPA2, RTG3, HTB1, SWC5, YKU80, MAC1, GET3, AGE1, SPS100, RPS14A
regulation of nucleobase, nucleoside, nucleotide and nucleic acid metabolic process	66 out of 353 genes, 18.7%	702 out of 6348 genes, 11.1%	0.00545	SNF6, KAR4, SUB2, BYE1, PHO85, SPT6, PRP45, MMS22, ARP6, HIF1, RGT1, TRA1, RAS2, ASC1, ARG5,6, MGS1, NOT3, CEG1, CBF1, PDR3, RIM101, KIN28, GTR2, SSK2, IRA2, XBP1, POL2, CDC39, HAT2, RRN5, MEC1, RTG1, MET4, CTK3, SNF2, SOK2, TAF14, RGR1, MSS11, UAF30, WAR1, NCB2, RME1, CDC7, CAF130, SPP1, VID28, PBS2, SAS5, HAP1, TOM1, DUN1, TAH11, ISW1, RPB7, TAF7, ELA1, NUT2, ARG80, SPT10, STB4, RTG3, HTB1, SWC5, MAC1, YKU80
regulation of metabolic process	75 out of 353 genes, 21.2%	832 out of 6348 genes, 13.1%	0.00548	SNF6, KAR4, SUB2, GCN1, BYE1, PHO85, SPT6, PRP45, MMS22, ARP6, HIF1, RGT1, TRA1, YKL107W, RAS2, ASC1, ARG5,6, MGS1, NOT3, CEG1, CBF1, PDR3, RIM101, KIN28, GTR2, MSS116, SSK2, IRA2, XBP1, POL2, CDC39, SLH1, HAT2, RRN5, MEC1, RTG1, MET4, CTK3, SNF2, SOK2, RGR1, TAF14, GLC7, MSS11, UAF30, HBS1, ARC18, WAR1, NCB2, RME1, CDC7, SLF1,

				SPP1, CAF130, PBS2, PIG1, VID28, SAS5, HAP1, DUN1, TOM1, TAH11, ISW1, TAF7, RPB7, ELA1, NUT2, ARG80, SPT10, STB4, RTG3, HTB1, SWC5, MAC1, YKU80
regulation of cellular metabolic process	73 out of 353 genes, 20.7%	804 out of 6348 genes, 12.7%	0.00577	SNF6, KAR4, SUB2, GCN1, BYE1, PHO85, SPT6, PRP45, MMS22, ARP6, HIF1, RGT1, TRA1, RAS2, ASC1, ARG5,6, MGS1, NOT3, CEG1, CBF1, PDR3, RIM101, KIN28, GTR2, MSS116, SSK2, IRA2, XBP1, POL2, CDC39, SLH1, HAT2, RRN5, MEC1, RTG1, MET4, CTK3, SNF2, SOK2, RGR1, TAF14, MSS11, UAF30, HBS1, ARC18, WAR1, NCB2, RME1, CDC7, SLF1, CAF130, SPP1, PBS2, PIG1, VID28, SAS5, HAP1, TOM1, DUN1, TAH11, ISW1, TAF7, RPB7, ELA1, NUT2, ARG80, SPT10, STB4, RTG3, HTB1, SWC5, MAC1, YKU80
positive regulation of RNA metabolic process	19 out of 353 genes, 5.4%	118 out of 6348 genes, 1.9%	0.01487	SNF6, CEG1, VID28, KAR4, CBF1, CTK3, MET4, SNF2, PDR3, HAP1, PRP45, RPB7, MSS11, KIN28, GTR2, ARG80, RAS2, NCB2, MAC1
regulation of biological process	98 out of 353 genes, 27.8%	1214 out of 6348 genes, 19.1%	0.02019	SNF6, KAR4, SUB2, GCN1, BYE1, PHO85, RAD24, SPT6, RSR1, PRP45, MMS22, ARP6, TUS1, BFA1, HIF1, RGT1, TRA1, YKL107W, TCO89, RPI1, NGR1, RAS2, ASC1, ARG5,6, MGS1, NOT3, CEG1, CBF1, PDR3, RIM101, KIN28, GTR2, MSS116, SSK2, IRA2, XBP1, POL2, CDC39, SLH1, TAX4, HAT2, RRN5, STE2, MEC1, RTG1, YPD1, MET4, CTK3, SNF2, SOK2, RGR1, TAF14, GLC7, ROM2, CYR1, MSS11, UAF30, GEM1, RGS2, HBS1, CBK1, ARC18, PTP2, WAR1, NCB2, RME1, CDC7, APC1, SLF1, SPP1, CAF130, PBS2, VID28, HBT1, PIG1, SAS5, IME2, HAP1, DUN1, TOM1, TAH11, ISW1, TAF7, RPB7, ELA1, YMR1, NUT2, ARG80, SPT10, STB4, SPA2, RTG3, HTB1, SWC5, YKU80, MAC1, GET3, AGE1
regulation of cellular process	96 out of 353 genes, 27.2%	1192 out of 6348 genes, 18.8%	0.02778	SNF6, KAR4, SUB2, GCN1, BYE1, PHO85, RAD24, SPT6, RSR1, PRP45, MMS22, ARP6, TUS1, BFA1, HIF1, RGT1, TRA1, TCO89, RPI1, RAS2, ASC1, ARG5,6, MGS1, NOT3, CEG1, CBF1, PDR3, RIM101, KIN28, GTR2, MSS116, SSK2, IRA2, XBP1, POL2, CDC39, SLH1, TAX4, HAT2, RRN5, STE2, MEC1, RTG1, YPD1, MET4, CTK3, SNF2, SOK2, RGR1, TAF14, GLC7, ROM2, CYR1, MSS11, UAF30, GEM1, RGS2, HBS1, CBK1, ARC18, WAR1, PTP2, NCB2, RME1, CDC7, APC1, SLF1, SPP1, CAF130, PBS2, VID28, HBT1, PIG1, SAS5, IME2, HAP1, DUN1, TOM1, TAH11, ISW1, TAF7, RPB7, ELA1, YMR1, NUT2, ARG80, SPT10, STB4, SPA2, RTG3, HTB1, SWC5, YKU80, MAC1,

				GET3, AGE1
