# Supplementary figures and images for: Performing statistical analyses on quantitative data in Taverna workflows: An example using R and maxdBrowse to identify differentially-expressed genes from microarray data
Source: BMC Bioinformatics. 2008 Aug 7;9:334. doi: 10.1186/1471-2105-9-334 (PMC2528018; doi:10.1186/1471-2105-9-334)

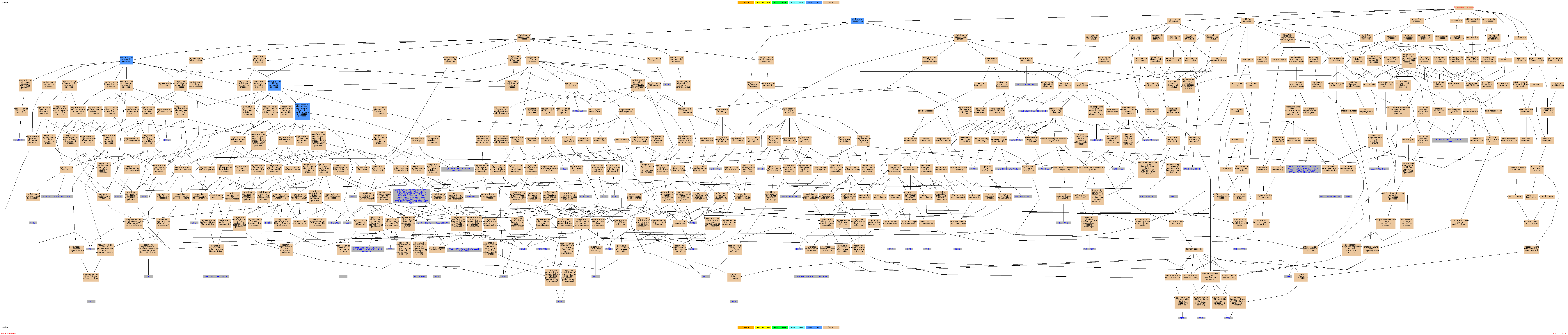

Supplement: Additional file 2 — Carbon t-test. [file 1471-2105-9-334-S2.zip › 0.01ttest/0.01Go/biolproc.png]

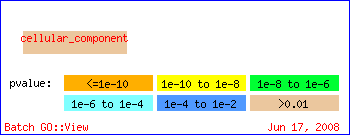

Supplement: Additional file 2 — Carbon t-test. [file 1471-2105-9-334-S2.zip › 0.01ttest/0.01Go/cellcomp.png]

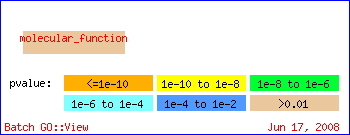

Supplement: Additional file 2 — Carbon t-test. [file 1471-2105-9-334-S2.zip › 0.01ttest/0.01Go/molfunc.png]
