# Supplementary material for: Performing statistical analyses on quantitative data in Taverna workflows: An example using R and maxdBrowse to identify differentially-expressed genes from microarray data
Source: BMC Bioinformatics. 2008 Aug 7;9:334. doi: 10.1186/1471-2105-9-334 (PMC2528018; doi:10.1186/1471-2105-9-334)
Supplement: Additional file 3 — Nitrogen t-test. [file 1471-2105-9-334-S3.zip › 0.05ttest/0.01Go/molfunc.pdf]

Result Table

Terms from the Function Ontology with p-value as good or better than 0.01

Gene Ontology term	Cluster frequency	Genome frequency of use	Corrected P-value	Genes annotated to the term
unannotated	24 out of 1943 genes, 1.2%	2 out of 6348 genes, 0.0%	0	YDL228C, ARS605, YFL015C, YKL033W-A_ALT, Q0270, YKR106W_1, YFL031W_EX2_ALT, PSY1, 2MIC_REP2, Q0155, TLC1_0, OPI6, YNL203C, Q0320, YRF, BUD30, MBB1, CEN13, YLR426W_EX2, Q0167, SNR17A_EX2, OPI8, TY1B_A_LR4, YEL074W
catalytic activity	860 out of 1943 genes, 44.3%	2371 out of 6348 genes, 37.4%	2.37e-11	ERG2, WRS1, ERG11, YLR126C, EEB1, YCF1, TUF1, RPT3, YPT1, IST3, RFS1, CRH1, BNA4, MSH2, IPP1, YIR035C, AST1, DFG5, DYS1, DPL1, AGX1, YNL217W, DNF1, CMP2, GCD10, ECM31, MSD1, TPK2, MKT1, PUS4, TGL2, MNT2, DED1, DMA1, ATF2, YFR006W, YPT32, TYW1, SDS3, QCR8, NTO1, TOS3, MET14, SME1, LYS1, COQ1, SMM1, CYM1, PUS7, CDC42, IMP1, YOR286W, CCP1, YNR071C, POL5, GLO4, NAT1, SEN15, SAM4, CDA1, YGR207C, YLR419W, ERG5, SKI2, SSC1, UTR1, CDC55, HSP78, FAB1, HIS3, DOG2, TRM3, CYR1, TPS3, UGA2, ADE6, VIP1, TRM12, ATP8, KAR2, LSG1, YPL236C, YTA6, GAL80, CMK1, HMS1, ALG14, ATP2, RIB4, PRS2, SWD3, SGS1, RKI1, DOA4, SOL3, GPM3, NDI1, SUV3, HOM3, BET4, BGL2, MSW1, MDL1, YIL165C, ENA5, MTQ1, RRD1, CWC27, PHO11, SAR1, GSH2, HDA2, ERG3, ALG6, AAD6, RET1, HOG1, YNL045W, RPA135, ENA1, LSM4, GLO1, IMD2, GLG1, YRF1-3, GPI18, CCA1, SUP35, RAS2, YJR142W, HSP104, PCK1, AI5_BETA, HEM13, RER2, PYK2, MDH2,

				ERG6, FAA4, CBR1, POP8, ADH4, ERV2, CET1, PTC6, EHT1, YOR285W, GPI11, IRE1, FSP2, ARG8, CTI6, YHB1, IDP1, SNF4, INO1, TEP1, MRPL3, CSL4, HNT1, HAT2, FAA3, RPC37, RPT4, YBR204C, FRE3, RAD16, LSM5, UTR2, MNN1, LEU1, PMT5, TKL1, TRP5, ILV5, MMS2, CCE1, NIT1, AXL1, KTR7, DIN7, PPA2, STE7, ZTA1, YRF1-2, TEL1, RSC30, SER33, RLI1, CDC36, CDC7, YFR007W, PRP5, UBP16, POT1, KTR1, FPR2, MAK31, DUR1,2, DBP5, BDS1, TUB3, YLR247C, ERG25, PGS1, HEM3, SCO1, GPI19, ATG26, BRR2, EFT2, PRS4, SET5, PRD1, ULP1, RHO3, MAK3, RSC9, KTR3, NOP1, UBR1, MCK1, NFT1, DAL3, ARO4, PHO85, YAL061W, NTG2, RHO5, YGL039W, CPR2, MNT3, YKR070W, TKL2, SWP1, SCEI, PRP28, ACO2, YDR307W, BIO3, SSL2, YPC1, RNH203, IPT1, HAT1, RPO31, ECM32, HEM4, UBA1, EXG2, ERG9, SMB1, PRI2, CDC1, YMR291W, RAD1, MSS116, SHR5, ADE12, IDH2, DIT2, PRP8, URA7, YRF1-7, LPP1, YPD1, GTO1, SNM1, YKL161C, GCD6, YKR104W, LAG1, TAF14, LAS21, PAN2, TAL1, RNT1, DLD2, IMD4, LEU4, AAD4, ERG7, SNT309, KRE6, UBP3, ATG5, IDI1, MOD5, RNR2, HPA3, MTG2, ACF2, ILV2, SER3, ADH7, HSL7, YMR130W, ARO2, PMT2, CDC21, APN1, ERF2, GRX3, PGI1, CYC7, ROT2, GRX5, FRE8, YBR025C, PRO2, YKU80, HPR5, DSF1, TOP3, INO80, YBR284W, SEN54, RNY1, PDB1, HSP31, CDS1, CTL1, RML2, QCR9, SAD1, HMX1, DGA1, YGR043C, SCW4, SKI6, ARO1, PMC1, VPS15, YMR085W, IDH1, SEN1, YHR020W, SAP155, COX17, PHS1, ALD2, TRM2, TGL3, HIS5, PRB1, SEC18, YJR149W, BST1, GIS1, MDM20, MNN9, UBP14,

				SNZ3, RCE1, HRT3, ADE5,7, CTM1, DSS1, SER1, VMA10, CPR3, GAD1, KCC4, FDH1, ESP1, SOD1, PDX3, PFS2, LPD1, GDA1, SSA2, RRI1, SHM1, OMS1, MXR1, GAS4, OMA1, ADE3, YNK1, YAH1, SNR6, ARD1, TSC13, FET5, LCB4, YNL274C, PTH1, FMP46, LRO1, MET7, YNL168C, KRE5, YNL024C, DUS3, NPR1, ISM1, PDA1, RPT6, SEC59, KAE1, CSH1, PMT6, TSA2, FSH2, MSS1, PRP6, TMT1, YOR059C, PRS3, ICL1, SNU114, GCV2, ARG1, DMA2, YLL056C, URA1, MTG1, ALD3, GET3, NCP1, SNF1, TDP1, DAP2, ADH3, HAS1, SIS2, SSU72, PDR5, GCN20, TRM10, AI4, PDE2, PMT1, GRE3, RPA190, CAR1, DAL2, YPS6, THR1, LEU2, AUS1, ILS1, TRA1, PIM1, MIS1, YPR004C, MNS1, YPT52, GRX4, RPB9, YPR127W, DBF20, ALG7, RPC82, DSE2, PSK2, MNN2, TYR1, KIN2, RHO4, RNR1, PFK2, GAR1, YPS3, HST2, COX15, MST1, CHS3, PTH2, YLR345W, NUP145, ARX1, DDI1, HOS1, TAT2, PPM1, PSD2, UBC13, DCS1, YGL157W, PRR1, NEO1, FMT1, SEC11, ADO1, MHT1, COX9, YSP3, RNR4, HEF3, RHR2, YRF1-6, KEX2, COR1, NPT1, MAG1, SDH4, FBP26, PRX1, ATP3, TAZ1, DLD1, CDC14, RAD57, SEC53, URA8, CDC4, NPL6, DBP2, KIN82, FPR4, GCD1, GPI16, SWF1, ERG12, ECM18, NTH1, RPB7, YNL247W, ILV3, OST3, CWH41, PDI1, SPT10, SOL4, URA2, BCS1, POP6, HEM1, GPD1, ELP3, TYS1, PAN5, AAT2, GUT2, LEU9, EST2, EPS1, PKH1, DFG10, PUS6, CDC50, MTF1, ATH1, WBP1, MSF1, YBR242W, YJL213W, CAR2, RSR1, HAM1, UBC8, ASN2, YPR118W, YCL074W, SIW14, CDC5, RSB1, CDC6, DPM1, NIT3, AST2, SIZ1, SLC1, ALD4, GSC2, LSC2, TRF5, AAP1, MKK1, AIR1, DRS2, RHO2,

				YHR044C, ACO1, PPG1, ARO3, NAM2, DRS1, YFR018C, GPH1, GLT1, FUN12, SMD3, YTA7, YAL049C, MGM1, RNR3, MIH1, RAD50, SCT1, DAL7, ALG9, YPL141C, FMS1, IMP2, PMT3, YOR283W, YOR1, GUK1, PGA3, PPH3, FAS2, HMG2, MEC1, PBN1, SUR2, GUS1, SLT2, DCS2, TUB1, MET10, DPS1, SSD1, CDC10, APT1, HIS2, RPN1, YRF1-1, UBP11, HFD1, MAK10, YGK3, ILV1, SNZ2, DEG1, PFA3, GAL83, MET2, DBP8, ICL2, NMA1, SLM5, PBS2, PPT1, ABP140, ECM38, RAD26, ASH1, RIB5, CDC26, HOM2, DSE4, LIP1, YPS1, RFC3, EFT1, SSA4, DIB1, ESS1, HXK1, UBC1, GPI14, YCK2, ZWF1, MKC7, ENA2, PHR1, REV7, AI2, POS5, MSE1, SKM1, ECI1, ROG1, ULA1, ALG2, GAL1, EHD3, SWD1, DAK2, RDH54, PRO1, DIA4, FRE2, PIB1, NAT2, HEM12, CTF18, AAH1, SIP1, RIB7, ARG5,6, KTR2, UBC6, APA1, STT3, MPD1, SFA1, PTC7, NDE1, ARO7, SDL1, ALG3, FAT1, PRK1, PDH1, YMR226C, HRR25, YPS5, ASP1, HSP82, CDC2, CHL1, DPB2, SMX2, HIS4, AYT1, YHR113W, CPR5, SLX8, SUR4, PFK1, PRE5, SLH1, PXA2, ALG5, DNF2, COX2, CHD1, YPR172W, MSP1, RKM2, SAP4, YBR139W, POP3, FKS1, ARG2, YIL064W, HOM6, GND1, STR3, YDR341C, RAD3, GDH1, PNG1, THR4, YBR014C, RIB3, YFR055W, ALG12, RPA43, YDR541C, AAD3, SWR1, MEF1, XYL2, ARF3, TOM1, SAK1, RPO21, PCM1, DUT1, YMR118C, ALG1, PAN6, PUS1, MRK1, CDC43, PHO12, AAT1, NDE2, PGM2, YMR041C, MCD4, PRP18, FAA2, HYS2, YDR061W, SCH9, PHO8, RPA49, SGA1, CAK1, VHS1, KRS1, HST4, TFG1, OYE2, PFK27, STV1, GLO2, SEN2, YKL071W, LEM3, FPR3,

				YKL033W-A, PET112, COX1, AAD15, AMD1, YJR107W, TRR2, COQ3, PCA1, ATG1, PSK1, ARH1, SNR19, CCC2, ATG3, ABD1, CDC39, YDR415C, RPC40, FRE4, VMA6, HPT1, VAS1, GAS2, RPA14, ISY1, SNO1, COX12, GPX1, MSM1, UBC9, URA6, DTD1, YKT6, YNL134C, ADE8, TAD3, LYS2, TOR1, COX7, SMX3, LYS12, ADH2, PPZ1, TSC10, SHM2, PTP2, NUS1, DIM1, CTK2, APC1, HOR2, YDL124W, YOR246C, YJL045W, GTO3, ATG7, NPY1, TPP1, INM1, ARG3, BDH1, GLY1, GPI12, MRM1, TPA1, PCS60, KTR4, SUC2, THS1, LIP5, FRS2, KTR6, GAL3
oxidoreductase activity	156 out of 1943 genes, 8.0%	335 out of 6348 genes, 5.3%	1.51e-07	ADH3, AI2, ERG11, RFS1, BNA4, YAL061W, YIR035C, AI4, AST1, GRE3, YGL039W, FRE2, LEU2, YPR004C, MIS1, GRX4, RIB7, ARG5,6, YPR127W, MPD1, SFA1, NDE1, TYR1, RNR1, YMR226C, TYW1, QCR8, ERG9, COX15, LYS1, SMM1, HIS4, IDH2, CCP1, DIT2, YGR207C, COX2, YPR172W, YGL157W, ERG5, COX9, RNR4, DLD2, IMD4, UGA2, HOM6, COR1, AAD4, GND1, SDH4, GDH1, PRX1, YBR014C, DLD1, RNR2, GAL80, YDR541C, AAD3, XYL2, SER3, ADH7, ARO2, NDI1, GRX3, PDI1, CYC7, YMR118C, GRX5, FRE8, PRO2, GPD1, DSF1, GUT2, PAN5, NDE2, PDB1, DFG10, YMR041C, HMX1, QCR9, ERG3, AAD6, ARO1, OYE2, IMD2, IDH1, YKL071W, COX17, ALD2, AI5_BETA, PCK1, AST2, HEM13, ALD4, COX1, YJR149W, AAD15, GIS1, MDH2, CBR1, ADH4, ERV2, TRR2, FDH1, SOD1, GLT1, ARH1, PDX3, LPD1, RNR3, YHB1, IDP1, FMS1, MXR1, PGA3, HMG2, FAS2, FRE4, FRE3, ADE3, YAH1, SUR2, TSC13, FET5, YNL274C, MET10, COX12, ILV5, GPX1, FMP46, HFD1, YNL134C, DUS3, LYS2, COX7, LYS12, PDA1,

				ADH2, ZTA1, TSC10, SER33, RLI1, TSA2, YDL124W, YOR246C, YJL045W, HOM2, BDH1, ERG25, GCV2, SCO1, TPA1, URA1, ZWF1, ALD3, NCP1
oxidoreductase activity, acting on CH-OH group of donors	47 out of 1943 genes, 2.4%	85 out of 6348 genes, 1.3%	0.00119	YMR041C, YGL157W, ADH3, AAD6, YNL274C, ILV5, YAL061W, ARO1, DLD2, GRE3, IMD4, YGL039W, YNL134C, IMD2, HOM6, IDH1, AAD4, GND1, LEU2, LYS12, ADH2, TSC10, RIB7, DLD1, YPR127W, AAD15, SER33, MDH2, SFA1, YDR541C, AAD3, YDL124W, ADH4, XYL2, ADH7, SER3, FDH1, BDH1, HIS4, IDH2, IDP1, GPD1, ZWF1, PAN5, GUT2, FAS2, HMG2
oxidoreductase activity, acting on the CH-OH group of donors, NAD or NADP as acceptor	44 out of 1943 genes, 2.3%	78 out of 6348 genes, 1.2%	0.00123	YMR041C, YGL157W, ADH3, AAD6, YNL274C, ILV5, YAL061W, ARO1, GRE3, IMD4, YGL039W, YNL134C, IMD2, HOM6, IDH1, AAD4, GND1, LEU2, LYS12, ADH2, TSC10, RIB7, YPR127W, AAD15, SER33, MDH2, SFA1, YDR541C, AAD3, YDL124W, ADH4, XYL2, ADH7, SER3, FDH1, BDH1, HIS4, IDH2, IDP1, GPD1, ZWF1, PAN5, FAS2, HMG2
transporter activity	175 out of 1943 genes, 9.0%	435 out of 6348 genes, 6.9%	0.00446	MTM1, CRC1, YCF1, PDR5, PSE1, NFT1, YRO2, HOL1, ITR1, SSU1, AUS1, DNF1, YKE4, USO1, ZRC1, HXT5, YDL119C, MRH1, TPC1, ENB1, HXT11, ARN1, NHA1, FLC1, FAT1, OPT1, ODC1, SNF3, QCR8, KAP95, AGP1, ITR2, SEC14, HUT1, FPS1, PXA2, TAT2, TPO3, DNF2, COX2, YOR271C, YPR011C, ATO3, NEO1, RIM2, COX9, YKR104W, SEC28, KAP122, ACB1, SLY41, COR1, ALP1, ATP8, AVT1, TOM20, ATP3, AVT7, SEC61, HXT16, YOR071C, TOM71, ATP2, HXT9, KAP120, SGE1, MUP1, AVT4, CAN1, YMC2, YVC1, DAL5, MDL1, YIL171W, KAP123, ENA5, TOM6, PHO84, COP1, TAT1, CDC50, QCR9, NMD5, OAC1,

				ENA1, PMC1, FCY22, AZR1, AQY2, MID1, CLC1, YIA6, STV1, LST7, SXM1, ADY2, LEM3, RSB1, YOL163W, YCR023C, RET2, COX1, APL3, DRS2, GAP1, BAP3, PTR2, VMA10, ZRG17, TIM9, TOM70, SUL1, PCA1, DTR1, QDR2, SMF1, VHT1, CCC2, TPO2, DAL4, VBA1, PHO89, YPR003C, AVT2, JEN1, HXT15, YOR1, LST4, TOM40, ODC2, COT1, SEC66, VPS73, VRG4, MCH1, VMA6, YOR378W, COX12, BAP2, RGT2, MDJ2, YIL166C, MCH5, MEP2, GNP1, COX7, YLL053C, FUI1, HXT13, FUR4, HXT12, MSN5, CTP1, HXT2, YEA6, SIT1, SEC21, SEC27, CTR3, YLR004C, FEN2, FCY2, ARN2, PIC2, VCX1, GAL2, YDR338C, DIC1, COX18, MAL11, YFL054C, AAC3, GET3, ENA2, SAM3
