# Supplementary material for: Performing statistical analyses on quantitative data in Taverna workflows: An example using R and maxdBrowse to identify differentially-expressed genes from microarray data
Source: BMC Bioinformatics. 2008 Aug 7;9:334. doi: 10.1186/1471-2105-9-334 (PMC2528018; doi:10.1186/1471-2105-9-334)
Supplement: Additional file 3 — Nitrogen t-test. [file 1471-2105-9-334-S3.zip › 0.05ttest/0.05Go/biolproc.pdf]

Result Table

Terms from the Process Ontology with p-value as good or better than 0.05				
**Gene Ontology term**	**Cluster frequency**	**Genome frequency of use**	**Corrected P-value**	**Genes annotated to the term**
unannotated	24 out of 1943 genes, 1.2%	2 out of 6348 genes, 0.0%	0	YDL228C, ARS605, YFL015C, YKL033W-A_ALT, Q0270, YKR106W_1, YFL031W_EX2_ALT, PSY1, 2MIC_REP2, Q0155, TLC1_0, OPI6, YNL203C, Q0320, YRF, BUD30, MBB1, CEN13, YLR426W_EX2, Q0167, SNR17A_EX2, OPI8, TY1B_A_LR4, YEL074W
cellular process	1611 out of 1943 genes, 82.9%	4831 out of 6348 genes, 76.1%	5.48e-15	SNX3, SDC25, SOH1, EEB1, TR(UCU)E, TUF1, SPC19, GCN4, DID2, URM1, BCH1, LOS1, IPP1, MUM2, SFI1, DFG5, DYS1, AGX1, HIT1, QRI5, DNF1, RTT102, ABF2, CMP2, AGA1, GCD10, MTO1, TAH1, MDM31, ISC10, PUS4, CLU1, MNT2, RPL13B, CBP6, DMA1, ATF2, STD1, IRS4, YPT32, TYW1, NTO1, TOS3, TK(CUU)J, MET14, TG(UCC)N, LYS1, TS(AGA)D2, CWP1, CYM1, CDC42, SEC14, CCP1, NAT1, SEN15, CDA1, SSC1, HSP78, FAB1, TRM3, CYR1, ATG11, BUD13, NET1, RMD6, CSG2, LSG1, ERD1, YPL236C, BDF1, URB1, GAL80, GCD7, CMK1, TE(UUC)J, ATP2, RIB4, GRC3, SGS1, GID7, RKI1, DOA4, SOL3, GPM3, CKS1, RNA1, SUV3, YDR161W, HOM3, MSW1, LSP1, TIF4632, YOL019W, PHO84, PET309, TQ(UUG)D3, RRD1, CWC27, SAR1, CCL1, HDA2, CAD1, RET1, YNL045W, ERP2, ENA1, VHR1, GLO1, IMD2, GLG1, SNF7, YIA6, MRP2, SNR51, GPI18, SPT20, SUP35, SWI6, RAS2, HSP104, PCK1, AI5_BETA, HEM13, RIM20, RER2, TR(ACG)K, TG(GCC)B, PYK2, MDH2, SEC31, ERG6, FAA4, LST8, ARC15, TFB3, POP8, REF2, ERV2, NDD1, TIM9, TOM70, CET1, PTC6, URE2, EHT1, GPI11, TI(AAU)L1, DSN1, ARG8, ZAP1, TH(GUG)M, YHB1, FOB1, IDP1, SNF4, INO1, STE12, MRPL3, CSL4, HAT2, ODC2, COT1, RPC37, SEC66, FRE3, RAD16, GLC8, UTR2, PMT5, PET10, RMI1, GOT1, TRP5, ILV5, MRP20, YTH1, NAB2, RLM1, SRB2, KTR7, DIN7, UBX6, KAP104, RLI1, MAM1, CDC7, MSN5, YFR007W, PRP5, MST27, POM152, UBP16, POT1, SNR11, KTR1, FPR2, MAK31, DUR1,2, FEN2, FMC1, BDS1, SSK1, ERG25, ATP11, RFM1, PIH1, HEM3, GPI19, ATG8, HUG1, ATG26, HTB1, PRS4, RHO3, RSC9, IVY1, PUB1, SUT2, UBR1, DAL3, ARO4, ISA1, RHO5, ECM9, YGL039W, RTT106, CPR2, SWI4, VTH2, SMC6, TD(GUC)J1, SEC39, TG(CCC)D, NCA3, BUL1, EMP70, MYO1, SGF29, BIO3, MOT3, YLH47, MNN4,

				RNH203, PSY4, MTR2, MFA2, IPT1, VPS38, EXG2, GOS1, SMB1, STF2, ERG9, YMR291W, HCR1, HEK2, MFT1, SHR5, RPS9B, ADE12, DIT2, TS(AGA)B, LPP1, YPD1, PEX12, RPL9A, LAS21, PAN2, TA(UGC)L, SLY41, TR(UCU)B, LEU4, ERG7, SNT309, KRE6, YTM1, TQ(UUG)D1, MRPL44, RNR2, ECM21, DCC1, FUS2, NOP16, ACF2, HTZ1, ILV2, SER3, SSP120, ADH7, HSL7, TIR4, TS(AGA)A, PMT2, STE4, ERF2, RRI2, GRX3, PGI1, SRM1, IMG2, RRN6, OSW2, FRE8, PRO2, YKU80, HPR5, EAF5, YBR284W, PDB1, SAC3, RAX1, CDS1, CTL1, YBP2, SAD1, YGR043C, TAF13, ARO1, YMR085W, ERV15, TS(AGA)L, CLN2, EAF7, IDH1, SKI3, SEN1, SXM1, YHR020W, GCR1, SNR56, SAP155, COX17, ERG28, MNP1, TRM2, TGL3, PRB1, SEC18, BST1, TE(CUC)D, NUP84, MNN9, ADE5,7, CTM1, DSS1, SER1, GIP3, SSE2, YOL054W, GAD1, NAS6, ESP1, PRP42, NIP1, PDX3, YHC1, DTR1, GDA1, SLM2, BRF1, SSA2, RRI1, HKR1, SHM1, BUD20, MAM33, BUR6, TOM40, MIA40, VID24, YAH1, SNR6, ARD1, TSC13, LCB4, MIF2, HSE1, KRE1, MDJ2, LRO1, TAF4, KRE5, NOP53, TT(AGU)J, CCT2, STF1, PDA1, BPH1, TM(CAU)J1, ECM11, RPS0A, NUP85, YLR278C, KAE1, POM34, GIS4, SEC21, CYK3, PRP6, RPL31B, SLX9, NUT2, ERJ5, TL(UAA)J, BUD8, ARG1, SPO12, COX18, DMA2, YLL056C, URA1, CNE1, AFT1, GET3, NCP1, SNF1, STE50, PAI3, DOC1, TDP1, SIS2, SSU72, MRP10, TRM10, PDE2, PMT1, TAP42, YPS6, ERV29, TRA1, ILS1, LEU2, ERP1, SLD5, ARP2, PCI8, USO1, MRPL33, BUD7, NRG2, RPB9, RPC82, RMD11, UTP13, UGA3, PSK2, MNN2, OPT1, TOS4, ESC8, PEX22, GAR1, HST2, COX15, MST1, CHS3, NOP14, KRE33, HOS1, TQ(UUG)D2, STE18, PDR8, TAT2, ORC5, PPM1, UBC13, PDS1, YGL157W, NEO1, SEC11, OSH6, NDC1, MSS18, ADO1, MHT1, COX9, CTF13, HAC1, RNR4, KAP122, HEF3, YRF1-6, AFG1, KEX2, COR1, MSH5, NSG1, TS(AGA)E, TOM20, SDH4, FBP26, ATP3, SEY1, DLD1, ZIP1, CDC14, LOC1, URA8, ENT1, DBP2, SDA1, KIN82, KAP120, HUR1, MED11, ERG12, YOR287C, MGR1, ILV3, YER184C, CWH41, PDI1, PHD1, YMC2, SPT8, URA2, BCS1, COY1, POP6, GPD1, HEM1, GUT2, TYS1, LEU9, EST2, VAM6, COP1, DFG10, PUS6, MBF1, WBP1, MSF1, TE(UUC)L, NSG2, GIP2, CAR2, YIF1, RRP1, RSR1, CLC1, YCL074W, SIW14, VPS20, CDC5, PCL1, RSB1, NNF2, NAB6, SNR61, MKK1, AIR1, DRS2, ACO1, PPG1, ARO3, DRS1, YFR018C, DAL81, KRI1, GPH1, GLT1, CUS2, FUN12, MAM3, TIF5, RPS30A, MCM16, SRB8, RNR3, MIH1, RAD50, SCT1, FMS1,

				YPL141C, PMT3, NUT1, IML3, YOR1, ERV41, JSN1, HMG2, MEC1, SPH1, PBN1, IKI1, SUR2, GUS1, SLT2, CSI1, DCS2, SSD1, CDC10, RPN1, YRF1-1, YRB2, UBP11, SUP45, HFD1, YGK3, TR(UCU)J1, HSM3, CUP9, GAL83, DBP8, TC(GCA)P1, ICL2, NMA1, CAF130, HPR1, PBS2, PPT1, MET18, MBA1, SCP160, MF(ALPHA)1, ECM38, RAD26, KAR1, YPS1, CWH43, HSP60, SSA4, DIB1, VTH1, BRE4, SWI5, AAC3, GTT3, TM(CAU)J3, PHR1, REV7, MSE1, ALG2, RPL13A, GAL1, DAK2, SFK1, CLB1, DIA4, RDH54, TIM21, PIB1, HEM12, ZRC1, CAC2, AAH1, SIP1, RIB7, ARG5,6, UBC6, RPS24B, STT3, APA1, SFA1, DOA1, NDE1, TQ(UUG)B, ARO7, SDL1, TE(UUC)C, EMP24, NHA1, PDR3, FAT1, PDH1, SYS1, HRR25, HSP82, CHL1, CLB2, SMX2, ATG19, ELP2, CPR5, SUR4, PRE5, AAR2, GAT1, CHD1, YPR172W, RPL16B, SEF1, CSF1, RIM2, IDS2, RKM2, SAP4, BIG1, SEC28, POP3, FKS1, ARG2, APS3, MRPS18, CSR2, RPL35A, RAD3, GDH1, PNG1, BOI1, VPS24, ALG12, AAD3, RGD2, MEF1, XYL2, TOM1, USA1, SAK1, RPO21, SED4, PCM1, CLN1, GIM3, YMR118C, PAN6, YBR033W, PUS1, MRK1, CDC43, HUB1, PGM2, GBP2, MCD4, PRP18, FAA2, ADD37, ISU2, IXR1, HYS2, PHO8, RPA49, RPL8A, CAK1, KRS1, TFG1, ATG18, RPS0B, STV1, GLO2, SEN2, LEM3, SEC17, GAT2, FPR3, PET112, BUD21, COX1, THI4, AAD15, AMD1, SEC13, HSP12, MBR1, TRR2, MET28, COQ3, ATG17, APQ12, ATG1, PCA1, SWA2, PSK1, ECM8, CCC2, SNR19, ATG3, RPL6B, AIP1, YHC3, BNR1, CDC39, YBL054W, TR(UCU)K, YOX1, YIP1, RPC40, VMA6, VAS1, HPT1, SSP1, ISY1, GEA2, MSM1, TH(GUG)E1, YKT6, TAD3, ADE8, LYS2, COX23, SMX3, LYS12, PPZ1, TN(GUU)K, PTP2, DOT6, YSC84, CTK2, APC1, UBX3, HOR2, YJL045W, INO4, LSM3, YDR089W, CDC31, ATG7, TG(GCC)M, INM1, CCT4, MLP1, TR(CCG)L, GLY1, GPI12, TP(AGG)C, STU1, TPA1, KTR4, SUC2, MAL11, ORC4, GAL3, WRS1, HOF1, ERG2, CRN1, ATG2, CRC1, YLR126C, ERG11, IST3, YPT1, RPG1, RPT3, TOS8, MAL33, MSH2, BNA4, CRH1, GET1, AST1, RPL5, DPL1, MDY2, ECM31, MSD1, TPK2, MKT1, MTL1, ENB1, TLG2, TS(AGA)J, DED1, ARN1, FLC1, FCF2, YFR006W, MET31, TG(GCC)C, MRPL25, SDS3, SNF3, QCR8, SME1, TEL2, COQ1, SMM1, PUS7, IMP1, GLO4, POL5, NHP10, TR(UCU)J2, SAM4, VIK1, ERG5, SKI2, PAC10, UTR1, CDC55, YPL144W, SEC16, HIS3, EFR3, DOG2, SSL1, ERP3, STB5, TG(UCC)O, TT(UGU)P, UGA2, TPS3, NNF1, SRL2, ATC1, ADE6, VIP1, TRM12, KAR2, ATP8,

				AVT1, SUT1, PEX11, YBR238C, SSN2, SEC61, UTP5, PCL5, HMS1, RPL9B, ALG14, ARG81, SEC24, UTP21, PRS2, SWD3, AME1, NDI1, YSF3, DAL80, JEM1, BET4, BGL2, VAM7, SPA2, RPS24A, KAP123, MTQ1, CLB4, PHO11, PEX10, GSH2, ERG3, NMD5, ALG6, RDS3, AAD6, MSC1, MSB3, HOG1, SNF11, RPL7B, SLS1, RPA135, LSM4, CFT1, YRF1-3, RGT1, RPL22B, CCA1, CWC23, RET2, EDC2, NRM1, ADH4, SIR1, IRE1, FSP2, IZH1, CTI6, SPT7, KIP1, UPC2, TEP1, HNT1, LST4, FAA3, RPT4, VRG4, LSM5, MNN1, MRPL20, LEU1, TKL1, MRPL40, MST28, RGT2, SHU2, MMS2, DAD4, CCE1, TSC3, TQ(UUG)E1, MEP2, AXL1, DBF4, STE7, PPA2, YRF1-2, PEP7, ILM1, TEL1, RSC30, SER33, CDC36, PET122, ENT5, CLB6, SEC27, WSC2, DER1, DBP5, PAC2, TUB3, NAS2, TR(UCU)M2, NOC4, RRP14, PGS1, SCO1, BRR2, EFT2, CHS6, TMA20, PRD1, ULP1, TG(GCC)P2, TR(ACG)D, GPG1, IOC4, MAK3, FIP1, MRPL37, RPL35B, DAD2, RSC58, NOP1, KTR3, NUP170, MCK1, PHO85, SNX41, NTG2, MNT3, TKL2, SWP1, SCEI, CCT6, PRP28, TE(UUC)P, ACO2, RRP5, SSL2, VTA1, TS(AGA)M, MRPL27, YPC1, DAN1, RPO31, HAT1, BEM4, HEM4, ODC1, YRM1, UBA1, SRB7, PRI2, PER1, CDC1, KAP95, RAD1, MF(ALPHA)2, MSS116, TV(CAC)D, SEC15, IDH2, HMRA1, PRP8, URA7, YNR063W, FPS1, MVB12, YRF1-7, RPS27B, TA(AGC)K2, NOP13, GTO1, SNM1, PHB2, MND2, YKL161C, TA(UGC)A, GCD6, PEX13, LAG1, CBC2, TAF14, TAL1, RNT1, DLD2, IMD4, AAD4, UBP3, ATG5, UTP15, IDI1, STO1, MOD5, DFM1, VMA22, SIP5, GIP4, HPA3, MSS51, SHC1, MTG2, ORC2, GCS1, HAL9, KNH1, BSP1, ARO2, CDC21, APN1, CYC7, ROT2, GRX5, RRB1, TOP3, INO80, SPT21, SEN54, RNY1, RGD1, SPT3, RML2, HMX1, QCR9, DGA1, HHT1, SCW4, SKI6, PMC1, VPS15, ECM7, TAF2, FYV6, ALD2, PHS1, HIS5, APL3, MDM20, GIS1, UBP14, VPS25, HRT3, RCE1, SNZ3, SEC12, VMA10, CPR3, YAP3, KCC4, FDH1, SOD1, SPT4, PFS2, LPD1, HSP26, URB2, SEC23, DAL4, MRPL39, VPS52, PBP1, OSH3, GAS4, CIN5, TS(AGA)D3, OMA1, YDR520C, SWH1, SEC9, ADE3, VPS73, YNK1, YNL274C, PTH1, THP1, PEA2, MET7, NUP157, MAF1, SUI3, OPY2, BOI2, SUA7, NBP1, DUS3, NPR1, ISM1, SBE22, RPT6, SEC59, LTE1, CSH1, PMT6, SLF1, CTP1, MSS1, GIS2, ICL1, PRS3, SNU114, VCX1, AFR1, WTM2, GCV2, LSB3, BUD25, ACN9, RSM10, MTG1, ERB1, ALD3, SUI1, PBI2, DAP2, PCL6, ADH3, MRPL8, HAS1, PSE1, GAL4, AI4, GRE3, CAR1, RPA190, DAL2, THR1, PCL8, RPS29A, MIS1, PIM1, MNS1,

				GIM4, YPT52, GRX4, VAM3, YJL103C, MFA1, DBF20, ALG7, DSE2, AGA2, TYR1, KIN2, TV(UAC)B, RHO4, RNR1, PFK2, YPS3, HHT2, THP2, PTH2, MOB1, YLR345W, NUP145, DDI1, ARX1, MDM35, PDS5, PSD2, DCS1, PRR1, RUB1, FMT1, YOR338W, YSP3, RHR2, TRE1, NPT1, MAG1, TAF10, TAZ1, YNL194C, PUF2, ADY3, TQ(UUG)C, RAD57, HYP2, TN(GUU)C, SEC53, CDC4, SGN1, NPL6, LGE1, FPR4, GCD1, ADD66, HSP10, GYP6, GPI16, SWF1, ECM18, NTH1, YNL247W, IML1, RPB7, UTP22, FZF1, COX20, OST3, MID2, YRR1, SPT10, SOL4, RIM8, ELP3, AAT2, PAN5, TG(GCC)J2, SHY1, TG(GCC)O2, EPS1, PKH1, PET111, CIN2, CDC50, MTF1, ATH1, YJL213W, TE(UUC)E1, PEP12, HAM1, UBC8, CCT5, SSF2, NUP192, ASN2, YPR118W, DLS1, MSI1, LST7, YLL054C, RPI1, CDC6, DPM1, NCE101, SIZ1, SLC1, RAD59, ALD4, DAL82, TE(UUC)M, SNR58, GSC2, LSC2, TRF5, AAP1, RHO2, YHR044C, NOG1, TR(UCU)D, NAM2, RPF1, MDM30, CSM4, RAD34, SMD3, YTA7, MGM1, RPL24B, BRR1, DAL7, TS(AGA)D1, ALG9, IMP2, MED2, SFB3, YOR283W, GUK1, PGA3, PPH3, FAS2, YOR008C-A, TUB1, TE(UUC)B, DPS1, MET10, TS(UGA)P, SFB2, APT1, HIS2, VAC8, UMP1, SWI1, MAK10, BFR1, ILV1, SNZ2, DEG1, PFA3, MIG3, RME1, MET2, SLM5, AZF1, ARP10, SIT1, ABP140, ASH1, YDJ1, RIB5, ACA1, CDC26, HOM2, DSE4, CNS1, AXL2, LIP1, GAL2, RFC3, ECM27, WSS1, EFT1, TG(CCC)O, ESS1, HXK1, DCN1, UBC1, HAP2, GPI14, YCK2, PRT1, ZWF1, MKC7, NUP100, MUC1, AI2, TR(UCU)M1, POS5, SKM1, ECI1, ULA1, YLR281C, EHD3, HSP42, ESF1, SWD1, PRO1, SSO2, STB2, NAT2, CTF18, RRF1, KTR2, CSE4, MPD1, MRPL11, TQ(UUG)E2, COG8, ALG3, PRK1, TA(UGC)O, MRP51, AHA1, YPS5, ASP1, CDC2, REC107, GTR2, DPB2, HIS4, PCL7, TIR3, VPS74, YHR113W, NRD1, SLX8, USE1, XBP1, PFK1, MSS2, TH(GUG)K, ALG5, DNF2, FAR1, COX2, SLM6, TAF6, MSP1, YBR139W, AEP2, NCS2, ACB1, VTC4, TIF6, YIL064W, HOM6, GND1, SPC97, STR3, YDR341C, EMG1, SGF11, THR4, YBR014C, RIB3, ASK10, PIL1, RPL27A, TFB1, YFR055W, EMI1, RPA43, IRR1, YDR541C, TL(GAG)G, CAT8, SWR1, ARF3, HAP1, NRG1, BUD2, ENT4, TRS120, AVT4, TCM62, DUT1, ALG1, YVC1, SOM1, NOC2, KOG1, TOM6, AAT1, NDE2, YMR041C, TS(UGA)E, RPL8B, RPL18A, HHO1, SCH9, RET3, OAC1, SGA1, VHS1, HST4, PFK27, RDS1, CLB5, YCS4, YMR31, SDS24, RPN4, TRS130, MRL1, RPS16B, RAD28, ARH1, TQ(UUG)L,

				MRPL50, RPS30B, ABD1, MSH4, IRA2, YDR415C, NMD2, SLI15, FRE4, NCA2, IST1, SPS4, GAS2, RFA2, RPA14, SNO1, COX12, RGR1, HCH1, UBC9, URA6, SUA5, DTD1, SRL3, TOR1, RRP9, COX7, ADH2, TSC10, SHM2, NUS1, DIM1, CAP2, SED1, HBT1, COS10, LRP1, SRN2, UTP20, GTO3, RAP1, NPY1, TPP1, ARG3, YFH1, BDH1, ARN2, TE(UUC)K, ARG80, MRM1, MRS1, ASF2, FLO8, IPI3, THS1, LIP5, SRP72, KTR6, FRS2
metabolic process	1339 out of 1943 genes, 68.9%	3912 out of 6348 genes, 61.6%	1.16e-12	SOH1, EEB1, TR(UCU)E, TUF1, GCN4, URM1, BCH1, LOS1, IPP1, MUM2, YIR035C, DFG5, DYS1, AGX1, QRI5, RTT102, GCD10, MTO1, TAH1, PUS4, CLU1, MNT2, RPL13B, CBP6, DMA1, ATF2, STD1, IRS4, TYW1, NTO1, TOS3, TK(CUU)J, MET14, TG(UCC)N, LYS1, TS(AGA)D2, CYM1, SEC14, CCP1, NAT1, SEN15, CDA1, YGR207C, ATO3, SSC1, HSP78, FAB1, TRM3, CYR1, BUD13, CSG2, ERD1, YPL236C, BDF1, URB1, GAL80, GCD7, CMK1, TE(UUC)J, ATP2, RIB4, GRC3, SGS1, GID7, RKI1, DOA4, SOL3, GPM3, CKS1, RNA1, SUV3, HOM3, MSW1, TIF4632, ENA5, YOL019W, PHO84, PET309, TQ(UUG)D3, RRD1, CWC27, CCL1, HDA2, CAD1, RET1, YNL045W, ENA1, VHR1, GLO1, IMD2, GLG1, SNF7, MRP2, SNR51, GPI18, SPT20, SUP35, SWI6, HSP104, PCK1, AI5_BETA, HEM13, RIM20, RER2, TR(ACG)K, TG(GCC)B, PYK2, MDH2, ERG6, FAA4, TFB3, POP8, REF2, ERV2, NDD1, CET1, PTC6, URE2, EHT1, GPI11, TI(AAU)L1, ARG8, ZAP1, TH(GUG)M, YHB1, FOB1, IDP1, SNF4, INO1, STE12, MRPL3, CSL4, HAT2, RPC37, YBR204C, FRE3, RAD16, GLC8, UTR2, PMT5, PET10, TRP5, ILV5, MRP20, YTH1, NAB2, NIT1, RLM1, SRB2, KTR7, DIN7, UBX6, RLI1, CDC7, YFR007W, PRP5, UBP16, POT1, SNR11, KTR1, FPR2, MAK31, DUR1,2, FMC1, BDS1, ERG25, ATP11, PIH1, HEM3, GPI19, ATG8, HUG1, ATG26, HTB1, PRS4, RSC9, PUB1, SUT2, UBR1, DAL3, ARO4, YAL061W, ISA1, YGL039W, RTT106, CPR2, SWI4, SMC6, TD(GUC)J1, TG(CCC)D, BUL1, SGF29, BIO3, MOT3, MNN4, RNH203, PSY4, IPT1, EXG2, SMB1, ERG9, STF2, YMR291W, HCR1, HEK2, MFT1, SHR5, RPS9B, ADE12, DIT2, TS(AGA)B, LPP1, RPL9A, LAS21, PAN2, TA(UGC)L, TR(UCU)B, LEU4, ERG7, SNT309, KRE6, TQ(UUG)D1, MRPL44, RNR2, DCC1, NOP16, ACF2, HTZ1, ILV2, SER3, ADH7, TS(AGA)A, PMT2, ERF2, RRI2, GRX3, PGI1, IMG2, RRN6, FRE8, PRO2, YKU80, HPR5, DSF1, EAF5, YBR284W, PDB1, SAC3, CDS1, CTL1, SAD1, YGR043C, TAF13, ARO1, YMR085W, TS(AGA)L, EAF7, IDH1, SKI3, SEN1, SXM1, YHR020W, GCR1,

				ADY2, SNR56, COX17, ERG28, MNP1, TRM2, TGL3, PRB1, YJR149W, BST1, TE(CUC)D, MNN9, ADE5,7, CTM1, DSS1, SER1, SSE2, YOL054W, GAD1, NAS6, ESP1, PRP42, NIP1, PDX3, YHC1, GDA1, BRF1, SSA2, RRI1, HKR1, SHM1, MAM33, BUR6, VID24, YAH1, SNR6, ARD1, TSC13, LCB4, LRO1, TAF4, KRE5, NOP53, TT(AGU)J, MAL23, CCT2, STF1, PDA1, TM(CAU)J1, ECM11, RPS0A, YLR278C, KAE1, SEC21, PRP6, RPL31B, SLX9, NUT2, ERJ5, TL(UAA)J, ARG1, DMA2, YLL056C, URA1, CNE1, AFT1, NCP1, SNF1, PAI3, DOC1, TDP1, SIS2, SSU72, MRP10, TRM10, PMT1, YPS6, TRA1, ILS1, LEU2, YPR004C, SLD5, PCI8, USO1, MRPL33, BUD7, NRG2, RPB9, RPC82, UTP13, UGA3, PSK2, MNN2, OPT1, TOS4, ESC8, PEX22, GAR1, HST2, COX15, MST1, CHS3, NOP14, HOS1, TQ(UUG)D2, PDR8, ORC5, PPM1, UBC13, PDS1, YGL157W, NEO1, SEC11, OSH6, MSS18, ADO1, MHT1, COX9, CTF13, HAC1, RNR4, HEF3, YRF1-6, AFG1, KEX2, COR1, NSG1, MSH5, TS(AGA)E, SDH4, FBP26, ATP3, DLD1, CDC14, URA8, DBP2, SDA1, KIN82, KAP120, HUR1, MED11, ERG12, YOR287C, ILV3, YER184C, CWH41, PDI1, PHD1, SPT8, URA2, BCS1, POP6, GPD1, HEM1, GUT2, TYS1, LEU9, EST2, COP1, DFG10, PUS6, MBF1, WBP1, MSF1, TE(UUC)L, NSG2, GIP2, CAR2, RRP1, CLC1, SIW14, VPS20, CDC5, NAB6, SNR61, MKK1, AIR1, DRS2, ACO1, PPG1, ARO3, DRS1, YFR018C, DAL81, KRI1, GPH1, GLT1, CUS2, FUN12, TIF5, RPS30A, SRB8, RNR3, MIH1, RAD50, SCT1, FMS1, YPL141C, PMT3, NUT1, JSN1, HMG2, MEC1, PBN1, IKI1, SUR2, GUS1, SLT2, CSI1, DCS2, RPN1, YRF1-1, UBP11, SUP45, HFD1, YGK3, TR(UCU)J1, HSM3, CUP9, GAL83, DBP8, TC(GCA)P1, ICL2, NMA1, CAF130, HPR1, PBS2, PPT1, MET18, MBA1, ECM38, RAD26, YPS1, CWH43, HSP60, SSA4, DIB1, SWI5, IES6, AAC3, GTT3, TM(CAU)J3, ENA2, PHR1, REV7, MSE1, ROG1, ALG2, RPL13A, GAL1, DAK2, DIA4, RDH54, PIB1, HEM12, ZRC1, CAC2, AAH1, SIP1, RIB7, ARG5,6, UBC6, RPS24B, STT3, APA1, SFA1, DOA1, NDE1, TQ(UUG)B, ARO7, SDL1, TE(UUC)C, PDR3, FAT1, PDH1, YMR226C, HRR25, HSP82, CHL1, SMX2, ATG19, ELP2, CPR5, SUR4, PRE5, AAR2, GAT1, CHD1, YPR172W, RPL16B, SEF1, CSF1, RKM2, SEC28, POP3, FKS1, ARG2, APS3, MRPS18, CSR2, RPL35A, RAD3, GDH1, PNG1, VPS24, ALG12, AAD3, MEF1, XYL2, TOM1, OAZ1, USA1, SAK1, RPO21, PCM1, GIM3, YMR118C, PAN6, YBR033W, PUS1, MRK1, CDC43, HUB1, PGM2, MCD4, PRP18, FAA2, ADD37, ISU2, IXR1, HYS2, PHO8, RPA49,

				RPL8A, CAK1, KRS1, TFG1, ATG18, RPS0B, GLO2, SEN2, YKL071W, GAT2, FPR3, YKL033W-A, PET112, BUD21, COX1, THI4, AAD15, AMD1, YJR107W, SEC13, MBR1, TRR2, MET28, COQ3, ATG1, PCA1, PSK1, CCC2, SNR19, ATG3, RPL6B, AIP1, CDC39, TR(UCU)K, YOX1, RPC40, VAS1, HPT1, SSP1, ISY1, MSM1, TH(GUG)E1, YNL134C, ADE8, TAD3, LYS2, COX23, SMX3, LYS12, TN(GUU)K, PTP2, DOT6, CTK2, APC1, UBX3, HOR2, YJL045W, INO4, LSM3, CDC31, ATG7, TG(GCC)M, INM1, CCT4, MLP1, TR(CCG)L, GLY1, GPI12, TP(AGG)C, TPA1, KTR4, SUC2, MAL11, ORC4, GAL3, WRS1, ERG2, YLR126C, ERG11, CRC1, IST3, YPT1, RPG1, RPT3, MAL33, MSH2, BNA4, CRH1, AST1, RPL5, DPL1, MDY2, ECM31, MSD1, TPK2, MKT1, TGL2, MPA43, TS(AGA)J, DED1, FLC1, FCF2, YFR006W, MET31, TG(GCC)C, MRPL25, SDS3, QCR8, SME1, COQ1, TEL2, SMM1, PUS7, IMP1, YNR071C, GLO4, POL5, NHP10, TR(UCU)J2, SAM4, SKI2, ERG5, UTR1, PAC10, CDC55, YPL144W, HIS3, DOG2, SSL1, STB5, TT(UGU)P, TG(UCC)O, UGA2, TPS3, SRL2, ADE6, VIP1, KAR2, ATP8, TRM12, SUT1, PEX11, YBR238C, SSN2, UTP5, PCL5, HMS1, RPL9B, ALG14, ARG81, UTP21, PRS2, SWD3, NDI1, YSF3, DAL80, JEM1, BET4, BGL2, RPS24A, YIL165C, KAP123, MTQ1, PHO11, GSH2, ERG3, NMD5, ALG6, RDS3, MSC1, AAD6, HOG1, SNF11, RPL7B, SLS1, RPA135, LSM4, CFT1, YRF1-3, RGT1, RPL22B, CCA1, CWC23, RET2, EDC2, NRM1, ADH4, SIR1, IRE1, FSP2, IZH1, CTI6, SPT7, UPC2, TEP1, HNT1, FAA3, RPT4, VRG4, LSM5, MNN1, LEU1, MRPL20, TKL1, MRPL40, SHU2, MMS2, CCE1, TSC3, TQ(UUG)E1, MEP2, AXL1, DBF4, STE7, ZTA1, PPA2, YRF1-2, TEL1, RSC30, SER33, CDC36, PET122, CLB6, SEC27, DER1, DBP5, NAS2, TUB3, PAC2, TR(UCU)M2, NOC4, PGS1, SCO1, BRR2, EFT2, CHS6, TMA20, PRD1, TG(GCC)P2, ULP1, TR(ACG)D, IOC4, MAK3, FIP1, MRPL37, RSC58, RPL35B, NOP1, KTR3, NUP170, MCK1, PHO85, NTG2, MNT3, YKR070W, TKL2, SCEI, CCT6, SWP1, PRP28, TE(UUC)P, ACO2, RRP5, SSL2, TS(AGA)M, MRPL27, YPC1, RPO31, HAT1, HEM4, YRM1, UBA1, SRB7, PRI2, PER1, CDC1, KAP95, RAD1, MSS116, TV(CAC)D, IDH2, HMRA1, PRP8, URA7, YNR063W, FPS1, YRF1-7, RPS27B, TA(AGC)K2, GTO1, SNM1, PHB2, MND2, YKL161C, TA(UGC)A, GCD6, LAG1, PEX13, CBC2, TAF14, TAL1, RNT1, DLD2, IMD4, AAD4, ATG5, UTP15, UBP3, IDI1, STO1, MOD5, VMA22, HPA3, MSS51, SHC1, MTG2,

				ORC2, KNH1, HAL9, SUE1, YMR130W, ARO2, CDC21, APN1, CYC7, ROT2, GRX5, RRB1, TOP3, INO80, SEN54, SPT3, RML2, HMX1, QCR9, DGA1, HHT1, SCW4, SKI6, PMC1, VPS15, TAF2, FYV6, ALD2, PHS1, HIS5, APL3, MDM20, GIS1, UBP14, VPS25, HRT3, RCE1, SNZ3, SEC12, VMA10, CPR3, YAP3, KCC4, FDH1, SOD1, SPT4, PFS2, LPD1, HSP26, URB2, DAL4, SEC23, MRPL39, PBP1, OSH3, MXR1, GAS4, CIN5, TS(AGA)D3, OMA1, YDR520C, SWH1, ADE3, YNK1, YNL274C, PTH1, THP1, MET7, YNL168C, MAF1, SUI3, SUA7, NPR1, DUS3, ISM1, RPT6, SEC59, CSH1, PMT6, SLF1, MSS1, YOR059C, ICL1, PRS3, SNU114, WTM2, GCV2, ACN9, RSM10, MTG1, ERB1, ALD3, SUI1, DAP2, PCL6, ADH3, MRPL8, HAS1, GAL4, AI4, GRE3, CAR1, RPA190, DAL2, THR1, PCL8, RPS29A, MIS1, PIM1, MNS1, GIM4, GRX4, YJL103C, DBF20, ALG7, TYR1, KIN2, TV(UAC)B, RNR1, PFK2, YPS3, HHT2, THP2, PTH2, YLR345W, MOB1, NUP145, DDI1, ARX1, PSD2, PRR1, DCS1, RUB1, FMT1, YSP3, RHR2, TRE1, NPT1, MAG1, TAF10, TAZ1, PUF2, ADY3, RAD57, HYP2, TQ(UUG)C, TN(GUU)C, CDC4, SGN1, SEC53, NPL6, LGE1, FPR4, ADD66, GCD1, HSP10, GPI16, SWF1, YNL247W, RPB7, NTH1, UTP22, PKR1, FZF1, OST3, COX20, YRR1, SPT10, SOL4, RIM8, ELP3, AAT2, PAN5, TG(GCC)J2, SHY1, TG(GCC)O2, EPS1, PKH1, PET111, CIN2, MTF1, ATH1, YJL213W, TE(UUC)E1, HAM1, UBC8, SSF2, CCT5, ASN2, YPR118W, DLS1, MSI1, YLL054C, RPI1, CDC6, DPM1, NIT3, SIZ1, AST2, RAD59, SLC1, ALD4, DAL82, TE(UUC)M, SNR58, GSC2, LSC2, AAP1, TRF5, YHR044C, NOG1, TR(UCU)D, NAM2, RPF1, MDM30, RAD34, SMD3, YTA7, RPL24B, BRR1, DAL7, TS(AGA)D1, ALG9, MED2, IMP2, YOR283W, GUK1, PPH3, FAS2, TUB1, TE(UUC)B, DPS1, MET10, TS(UGA)P, HIS2, APT1, UMP1, SWI1, MAK10, BFR1, ILV1, SNZ2, DEG1, PFA3, MIG3, RME1, MET2, SLM5, AZF1, ASH1, YDJ1, RIB5, ACA1, CDC26, HOM2, DSE4, CNS1, LIP1, GAL2, RFC3, WSS1, EFT1, TG(CCC)O, ESS1, HXK1, DCN1, UBC1, HAP2, YCK2, GPI14, ZWF1, PRT1, MKC7, AI2, TR(UCU)M1, POS5, ECI1, SKM1, ULA1, YLR281C, EHD3, ESF1, SWD1, PRO1, FRE2, STB2, NAT2, CTF18, RRF1, KTR2, CSE4, MPD1, MRPL11, TQ(UUG)E2, ALG3, PRK1, TA(UGC)O, MRP51, AHA1, YPS5, ASP1, CDC2, REC107, DPB2, HIS4, AYT1, PCL7, VPS74, YHR113W, NRD1, SLX8, XBP1, PFK1, TH(GUG)K, ALG5, COX2, SLM6, TAF6, YBR139W, AEP2, NCS2, ACB1, TIF6, YIL064W, HOM6, VMA21, GND1, STR3, YDR341C,

				EMG1, SGF11, THR4, RIB3, YBR014C, ASK10, RPL27A, YFR055W, TFB1, RPA43, YDR541C, TL(GAG)G, CAT8, SWR1, HAP1, NRG1, TCM62, DUT1, ALG1, SOM1, NOC2, AAT1, NDE2, YMR041C, TS(UGA)E, RPL8B, RPL18A, HHO1, SCH9, RET3, SGA1, VHS1, HST4, OYE2, PFK27, RDS1, ECM29, CLB5, YCS4, YMR31, RPN4, RPS16B, RAD28, ARH1, TQ(UUG)L, MRPL50, ABD1, RPS30B, MSH4, YDR415C, NMD2, FRE4, SLI15, NCA2, IST1, RPA14, RFA2, SNO1, COX12, RGR1, HCH1, URA6, UBC9, SUA5, SRL3, DTD1, RRP9, COX7, ADH2, TSC10, SHM2, NUS1, DIM1, YDL124W, LRP1, SRN2, YOR246C, UTP20, GTO3, RAP1, NPY1, TPP1, ARG3, YFH1, BDH1, ARN2, TE(UUC)K, MRM1, MRS1, ARG80, PCS60, FLO8, IPI3, THS1, LIP5, SRP72, KTR6, FRS2
cellular metabolic process	1245 out of 1943 genes, 64.1%	3690 out of 6348 genes, 58.1%	1.27e-07	SOH1, EEB1, TR(UCU)E, TUF1, GCN4, URM1, BCH1, LOS1, IPP1, MUM2, DYS1, AGX1, QRI5, RTT102, GCD10, MTO1, TAH1, PUS4, CLU1, MNT2, RPL13B, CBP6, DMA1, ATF2, STD1, TYW1, NTO1, TOS3, TK(CUU)J, MET14, TG(UCC)N, LYS1, TS(AGA)D2, CYM1, SEC14, CCP1, NAT1, SEN15, CDA1, SSC1, HSP78, FAB1, TRM3, CYR1, BUD13, CSG2, ERD1, YPL236C, BDF1, URB1, GAL80, GCD7, CMK1, TE(UUC)J, ATP2, RIB4, GRC3, SGS1, GID7, RKI1, DOA4, SOL3, GPM3, CKS1, RNA1, SUV3, HOM3, MSW1, TIF4632, YOL019W, PHO84, PET309, TQ(UUG)D3, RRD1, CWC27, CCL1, HDA2, CAD1, RET1, YNL045W, VHR1, GLO1, IMD2, GLG1, SNF7, MRP2, SNR51, GPI18, SPT20, SUP35, SWI6, HSP104, PCK1, AI5_BETA, HEM13, RIM20, RER2, TG(GCC)B, TR(ACG)K, PYK2, MDH2, ERG6, FAA4, TFB3, POP8, REF2, ERV2, NDD1, CET1, PTC6, URE2, EHT1, GPI11, TI(AAU)L1, ARG8, ZAP1, TH(GUG)M, YHB1, FOB1, IDP1, SNF4, INO1, STE12, MRPL3, CSL4, HAT2, RPC37, RAD16, GLC8, PMT5, PET10, TRP5, ILV5, MRP20, YTH1, NAB2, RLM1, SRB2, KTR7, DIN7, UBX6, RLI1, CDC7, YFR007W, PRP5, UBP16, POT1, SNR11, KTR1, FPR2, MAK31, DUR1,2, FMC1, BDS1, ERG25, ATP11, PIH1, HEM3, GPI19, ATG8, HUG1, ATG26, HTB1, PRS4, RSC9, PUB1, SUT2, UBR1, DAL3, ARO4, ISA1, YGL039W, RTT106, CPR2, SWI4, SMC6, TD(GUC)J1, TG(CCC)D, BUL1, SGF29, BIO3, MOT3, MNN4, RNH203, PSY4, IPT1, ERG9, STF2, SMB1, YMR291W, HCR1, HEK2, MFT1, SHR5, RPS9B, ADE12, TS(AGA)B, LPP1, RPL9A, LAS21, PAN2, TA(UGC)L, TR(UCU)B, LEU4, ERG7, SNT309, KRE6, TQ(UUG)D1, MRPL44, RNR2, DCC1, NOP16, HTZ1, ILV2, SER3, ADH7, TS(AGA)A, PMT2, ERF2,

				RRI2, PGI1, IMG2, RRN6, PRO2, YKU80, HPR5, EAF5, YBR284W, PDB1, SAC3, CDS1, CTL1, SAD1, YGR043C, TAF13, ARO1, YMR085W, TS(AGA)L, EAF7, IDH1, SKI3, SEN1, SXM1, YHR020W, GCR1, SNR56, COX17, ERG28, MNP1, TRM2, TGL3, PRB1, BST1, TE(CUC)D, MNN9, ADE5,7, CTM1, DSS1, SER1, SSE2, YOL054W, GAD1, NAS6, ESP1, PRP42, NIP1, PDX3, YHC1, GDA1, BRF1, SSA2, RRI1, HKR1, SHM1, MAM33, BUR6, VID24, YAH1, SNR6, ARD1, TSC13, LCB4, LRO1, TAF4, KRE5, NOP53, TT(AGU)J, CCT2, STF1, PDA1, TM(CAU)J1, ECM11, RPS0A, YLR278C, KAE1, PRP6, RPL31B, SLX9, NUT2, ERJ5, TL(UAA)J, ARG1, DMA2, YLL056C, URA1, CNE1, AFT1, NCP1, SNF1, PAI3, DOC1, TDP1, SIS2, SSU72, MRP10, TRM10, PMT1, YPS6, TRA1, LEU2, ILS1, SLD5, PCI8, MRPL33, BUD7, NRG2, RPB9, RPC82, UTP13, PSK2, UGA3, MNN2, OPT1, TOS4, ESC8, GAR1, HST2, COX15, MST1, CHS3, NOP14, HOS1, TQ(UUG)D2, PDR8, ORC5, PPM1, UBC13, PDS1, YGL157W, SEC11, OSH6, MSS18, ADO1, MHT1, COX9, CTF13, HAC1, RNR4, HEF3, YRF1-6, AFG1, KEX2, COR1, NSG1, MSH5, TS(AGA)E, SDH4, FBP26, ATP3, DLD1, CDC14, URA8, DBP2, KIN82, KAP120, HUR1, ERG12, MED11, YOR287C, ILV3, YER184C, CWH41, PDI1, PHD1, SPT8, URA2, BCS1, POP6, GPD1, HEM1, GUT2, TYS1, LEU9, EST2, PUS6, MBF1, WBP1, MSF1, TE(UUC)L, NSG2, GIP2, CAR2, RRP1, SIW14, VPS20, CDC5, NAB6, SNR61, MKK1, AIR1, ACO1, PPG1, ARO3, DRS1, YFR018C, DAL81, KRI1, GPH1, GLT1, CUS2, FUN12, TIF5, RPS30A, SRB8, RNR3, MIH1, RAD50, SCT1, FMS1, YPL141C, PMT3, NUT1, JSN1, HMG2, MEC1, PBN1, IKI1, SUR2, GUS1, SLT2, CSI1, DCS2, RPN1, YRF1-1, UBP11, SUP45, HFD1, YGK3, TR(UCU)J1, HSM3, CUP9, GAL83, DBP8, TC(GCA)P1, ICL2, NMA1, CAF130, HPR1, PBS2, PPT1, MET18, MBA1, ECM38, RAD26, YPS1, CWH43, HSP60, SSA4, DIB1, SWI5, AAC3, GTT3, TM(CAU)J3, PHR1, REV7, MSE1, ALG2, RPL13A, GAL1, DAK2, DIA4, RDH54, PIB1, ZRC1, HEM12, CAC2, AAH1, SIP1, RIB7, ARG5,6, UBC6, RPS24B, APA1, STT3, SFA1, DOA1, NDE1, TQ(UUG)B, ARO7, SDL1, TE(UUC)C, PDR3, FAT1, PDH1, HRR25, HSP82, CHL1, SMX2, ATG19, ELP2, CPR5, SUR4, PRE5, AAR2, GAT1, CHD1, YPR172W, RPL16B, SEF1, CSF1, RKM2, POP3, FKS1, ARG2, MRPS18, CSR2, RPL35A, RAD3, GDH1, PNG1, VPS24, ALG12, AAD3, MEF1, XYL2, TOM1, USA1, SAK1, RPO21, PCM1, GIM3, YMR118C, PAN6, YBR033W, PUS1, MRK1, CDC43, HUB1, PGM2,

				MCD4, PRP18, FAA2, ADD37, ISU2, IXR1, HYS2, PHO8, RPA49, RPL8A, CAK1, KRS1, TFG1, ATG18, RPS0B, GLO2, SEN2, GAT2, FPR3, PET112, BUD21, COX1, THI4, AAD15, AMD1, SEC13, MBR1, TRR2, MET28, COQ3, ATG1, PSK1, SNR19, ATG3, RPL6B, AIP1, CDC39, TR(UCU)K, YOX1, RPC40, VAS1, HPT1, ISY1, MSM1, TH(GUG)E1, ADE8, TAD3, LYS2, COX23, SMX3, LYS12, TN(GUU)K, PTP2, DOT6, CTK2, APC1, UBX3, HOR2, YJL045W, INO4, LSM3, CDC31, ATG7, TG(GCC)M, INM1, CCT4, MLP1, TR(CCG)L, GLY1, GPI12, TP(AGG)C, TPA1, KTR4, SUC2, MAL11, ORC4, GAL3, WRS1, ERG2, YLR126C, ERG11, CRC1, IST3, RPG1, RPT3, MSH2, BNA4, MAL33, RPL5, DPL1, MDY2, ECM31, MSD1, TPK2, MKT1, TS(AGA)J, DED1, FLC1, FCF2, YFR006W, MET31, TG(GCC)C, MRPL25, SDS3, QCR8, SME1, COQ1, TEL2, SMM1, PUS7, IMP1, GLO4, POL5, NHP10, TR(UCU)J2, SAM4, SKI2, ERG5, UTR1, PAC10, CDC55, YPL144W, HIS3, DOG2, SSL1, STB5, TT(UGU)P, TG(UCC)O, UGA2, TPS3, SRL2, ADE6, VIP1, KAR2, ATP8, TRM12, SUT1, PEX11, YBR238C, SSN2, UTP5, PCL5, HMS1, RPL9B, ALG14, ARG81, UTP21, PRS2, SWD3, NDI1, YSF3, DAL80, JEM1, BET4, RPS24A, KAP123, MTQ1, PHO11, GSH2, ERG3, NMD5, ALG6, MSC1, AAD6, RDS3, HOG1, SNF11, RPL7B, SLS1, RPA135, LSM4, CFT1, YRF1-3, RGT1, RPL22B, CCA1, CWC23, EDC2, NRM1, ADH4, SIR1, IRE1, FSP2, CTI6, SPT7, UPC2, TEP1, HNT1, FAA3, RPT4, VRG4, LSM5, MNN1, LEU1, MRPL20, TKL1, MRPL40, SHU2, MMS2, CCE1, TSC3, TQ(UUG)E1, AXL1, DBF4, STE7, PPA2, YRF1-2, TEL1, RSC30, SER33, CDC36, PET122, CLB6, DER1, DBP5, NAS2, TUB3, PAC2, TR(UCU)M2, NOC4, PGS1, SCO1, BRR2, EFT2, CHS6, TMA20, PRD1, TG(GCC)P2, ULP1, TR(ACG)D, MAK3, IOC4, FIP1, MRPL37, RSC58, RPL35B, NOP1, KTR3, NUP170, MCK1, PHO85, NTG2, MNT3, TKL2, SCEI, CCT6, SWP1, TE(UUC)P, PRP28, ACO2, RRP5, SSL2, TS(AGA)M, MRPL27, YPC1, RPO31, HAT1, HEM4, YRM1, UBA1, SRB7, PRI2, PER1, CDC1, KAP95, RAD1, MSS116, TV(CAC)D, IDH2, HMRA1, PRP8, URA7, YNR063W, FPS1, YRF1-7, RPS27B, GTO1, TA(AGC)K2, PHB2, SNM1, MND2, YKL161C, TA(UGC)A, GCD6, LAG1, PEX13, CBC2, TAF14, TAL1, RNT1, DLD2, IMD4, AAD4, ATG5, UTP15, UBP3, IDI1, STO1, MOD5, VMA22, HPA3, MSS51, SHC1, MTG2, ORC2, KNH1, HAL9, ARO2, CDC21, APN1, CYC7, GRX5, RRB1, TOP3, INO80, SEN54, SPT3, RML2, HMX1, QCR9, DGA1, HHT1, SKI6, VPS15, TAF2, FYV6, ALD2,

				PHS1, HIS5, MDM20, GIS1, UBP14, VPS25, HRT3, RCE1, SNZ3, SEC12, VMA10, CPR3, YAP3, KCC4, FDH1, SOD1, SPT4, PFS2, LPD1, HSP26, URB2, DAL4, SEC23, MRPL39, PBP1, OSH3, CIN5, TS(AGA)D3, OMA1, YDR520C, SWH1, ADE3, YNK1, YNL274C, PTH1, THP1, MET7, MAF1, SUI3, SUA7, NPR1, DUS3, ISM1, RPT6, SEC59, CSH1, PMT6, SLF1, MSS1, ICL1, PRS3, SNU114, WTM2, GCV2, ACN9, RSM10, MTG1, ERB1, ALD3, SUI1, DAP2, PCL6, ADH3, MRPL8, HAS1, GAL4, AI4, GRE3, CAR1, RPA190, DAL2, THR1, PCL8, RPS29A, MIS1, PIM1, MNS1, GIM4, YJL103C, DBF20, ALG7, TYR1, KIN2, TV(UAC)B, RNR1, PFK2, YPS3, HHT2, THP2, PTH2, YLR345W, MOB1, NUP145, DDI1, ARX1, PSD2, PRR1, DCS1, FMT1, RUB1, YSP3, RHR2, TRE1, NPT1, MAG1, TAF10, TAZ1, PUF2, RAD57, HYP2, TQ(UUG)C, TN(GUU)C, CDC4, SGN1, SEC53, NPL6, LGE1, FPR4, ADD66, GCD1, HSP10, GPI16, SWF1, YNL247W, RPB7, NTH1, UTP22, FZF1, OST3, COX20, YRR1, SPT10, SOL4, RIM8, AAT2, PAN5, TG(GCC)J2, ELP3, SHY1, TG(GCC)O2, EPS1, PKH1, PET111, CIN2, MTF1, ATH1, YJL213W, TE(UUC)E1, HAM1, UBC8, CCT5, ASN2, YPR118W, DLS1, MSI1, YLL054C, RPI1, CDC6, DPM1, SIZ1, RAD59, SLC1, ALD4, DAL82, TE(UUC)M, SNR58, GSC2, LSC2, AAP1, TRF5, YHR044C, NOG1, TR(UCU)D, NAM2, RPF1, MDM30, RAD34, SMD3, RPL24B, BRR1, DAL7, TS(AGA)D1, ALG9, MED2, IMP2, YOR283W, GUK1, PPH3, FAS2, TUB1, TE(UUC)B, DPS1, MET10, TS(UGA)P, HIS2, APT1, UMP1, SWI1, MAK10, ILV1, BFR1, SNZ2, DEG1, PFA3, MIG3, RME1, MET2, SLM5, AZF1, ASH1, YDJ1, RIB5, ACA1, CDC26, HOM2, CNS1, LIP1, GAL2, RFC3, WSS1, EFT1, TG(CCC)O, ESS1, HXK1, DCN1, UBC1, YCK2, GPI14, HAP2, ZWF1, PRT1, MKC7, AI2, TR(UCU)M1, POS5, ECI1, SKM1, ULA1, YLR281C, EHD3, ESF1, SWD1, PRO1, STB2, NAT2, CTF18, RRF1, KTR2, MPD1, MRPL11, TQ(UUG)E2, ALG3, PRK1, TA(UGC)O, AHA1, MRP51, YPS5, ASP1, CDC2, REC107, DPB2, HIS4, PCL7, VPS74, YHR113W, NRD1, SLX8, XBP1, PFK1, TH(GUG)K, ALG5, COX2, SLM6, TAF6, YBR139W, AEP2, NCS2, ACB1, TIF6, HOM6, STR3, GND1, YDR341C, EMG1, SGF11, THR4, RIB3, ASK10, RPL27A, YFR055W, TFB1, RPA43, YDR541C, TL(GAG)G, CAT8, SWR1, HAP1, NRG1, TCM62, DUT1, ALG1, SOM1, AAT1, NDE2, YMR041C, TS(UGA)E, RPL8B, RPL18A, SCH9, SGA1, VHS1, HST4, PFK27, RDS1, CLB5, YCS4, YMR31, RPN4, RPS16B, RAD28, TQ(UUG)L,

				MRPL50, ABD1, RPS30B, MSH4, YDR415C, NMD2, SLI15, NCA2, IST1, RPA14, RFA2, SNO1, COX12, RGR1, HCH1, URA6, UBC9, SUA5, SRL3, DTD1, RRP9, COX7, ADH2, SHM2, TSC10, DIM1, SRN2, LRP1, UTP20, GTO3, RAP1, NPY1, TPP1, ARG3, YFH1, BDH1, ARN2, TE(UUC)K, MRS1, MRM1, ARG80, FLO8, IPI3, THS1, LIP5, SRP72, KTR6, FRS2
primary metabolic process	1195 out of 1943 genes, 61.5%	3550 out of 6348 genes, 55.9%	2.08e-06	SOH1, EEB1, TR(UCU)E, TUF1, GCN4, URM1, BCH1, LOS1, MUM2, DYS1, AGX1, QRI5, RTT102, GCD10, MTO1, TAH1, PUS4, CLU1, MNT2, RPL13B, CBP6, DMA1, ATF2, STD1, IRS4, TYW1, NTO1, TOS3, TK(CUU)J, MET14, TG(UCC)N, LYS1, TS(AGA)D2, CYM1, SEC14, NAT1, SEN15, CDA1, SSC1, HSP78, FAB1, TRM3, CYR1, BUD13, CSG2, ERD1, YPL236C, BDF1, URB1, GAL80, GCD7, CMK1, TE(UUC)J, ATP2, GRC3, SGS1, GID7, RKI1, DOA4, SOL3, GPM3, CKS1, RNA1, SUV3, HOM3, MSW1, TIF4632, YOL019W, PET309, TQ(UUG)D3, RRD1, CWC27, CCL1, HDA2, CAD1, RET1, YNL045W, GLO1, IMD2, GLG1, SNF7, MRP2, SNR51, GPI18, SPT20, SUP35, SWI6, HSP104, PCK1, AI5_BETA, RIM20, RER2, TG(GCC)B, TR(ACG)K, PYK2, MDH2, ERG6, FAA4, TFB3, POP8, REF2, ERV2, NDD1, CET1, PTC6, URE2, EHT1, GPI11, TI(AAU)L1, ARG8, ZAP1, TH(GUG)M, FOB1, IDP1, SNF4, INO1, STE12, MRPL3, CSL4, HAT2, RPC37, YBR204C, RAD16, GLC8, UTR2, PMT5, TRP5, ILV5, MRP20, YTH1, NAB2, RLM1, SRB2, KTR7, DIN7, UBX6, RLI1, CDC7, PRP5, UBP16, POT1, SNR11, KTR1, FPR2, MAK31, DUR1,2, FMC1, ERG25, ATP11, PIH1, GPI19, ATG8, HUG1, ATG26, HTB1, PRS4, RSC9, PUB1, SUT2, UBR1, DAL3, ARO4, RTT106, CPR2, SWI4, SMC6, TD(GUC)J1, TG(CCC)D, BUL1, SGF29, MOT3, MNN4, RNH203, PSY4, IPT1, EXG2, ERG9, STF2, SMB1, YMR291W, HCR1, HEK2, MFT1, SHR5, RPS9B, ADE12, TS(AGA)B, LPP1, RPL9A, LAS21, PAN2, TA(UGC)L, TR(UCU)B, LEU4, ERG7, SNT309, KRE6, TQ(UUG)D1, MRPL44, RNR2, DCC1, NOP16, HTZ1, ILV2, SER3, TS(AGA)A, PMT2, ERF2, RRI2, PGI1, IMG2, RRN6, PRO2, YKU80, HPR5, EAF5, YBR284W, PDB1, SAC3, CDS1, CTL1, SAD1, YGR043C, TAF13, ARO1, YMR085W, TS(AGA)L, EAF7, IDH1, SKI3, SEN1, SXM1, YHR020W, GCR1, SNR56, COX17, ERG28, MNP1, TRM2, TGL3, PRB1, BST1, TE(CUC)D, MNN9, ADE5,7, CTM1, DSS1, SER1, SSE2, YOL054W, GAD1, NAS6, ESP1, PRP42, NIP1, PDX3, YHC1, GDA1, BRF1, SSA2, RRI1, HKR1, SHM1, BUR6, VID24, SNR6, ARD1, TSC13, LCB4, LRO1, TAF4, KRE5, NOP53, TT(AGU)J,

				MAL23, CCT2, STF1, PDA1, TM(CAU)J1, ECM11, RPS0A, YLR278C, KAE1, SEC21, PRP6, RPL31B, SLX9, NUT2, ERJ5, TL(UAA)J, ARG1, DMA2, URA1, CNE1, AFT1, NCP1, SNF1, PAI3, DOC1, TDP1, SSU72, MRP10, TRM10, PMT1, YPS6, TRA1, LEU2, ILS1, SLD5, PCI8, USO1, MRPL33, BUD7, NRG2, RPB9, RPC82, UTP13, PSK2, UGA3, MNN2, TOS4, ESC8, GAR1, HST2, COX15, MST1, CHS3, NOP14, HOS1, TQ(UUG)D2, PDR8, ORC5, PPM1, UBC13, PDS1, SEC11, OSH6, MSS18, ADO1, MHT1, CTF13, HAC1, RNR4, HEF3, YRF1-6, AFG1, KEX2, COR1, NSG1, MSH5, TS(AGA)E, FBP26, ATP3, DLD1, CDC14, URA8, DBP2, KIN82, KAP120, HUR1, ERG12, MED11, YOR287C, ILV3, YER184C, CWH41, PDI1, PHD1, SPT8, URA2, BCS1, POP6, GPD1, GUT2, TYS1, LEU9, EST2, COP1, DFG10, PUS6, MBF1, WBP1, MSF1, TE(UUC)L, NSG2, GIP2, CAR2, RRP1, CLC1, SIW14, VPS20, CDC5, NAB6, SNR61, MKK1, AIR1, ACO1, PPG1, ARO3, DRS1, YFR018C, DAL81, KRI1, GPH1, GLT1, CUS2, FUN12, TIF5, RPS30A, SRB8, RNR3, MIH1, RAD50, SCT1, FMS1, YPL141C, PMT3, NUT1, JSN1, HMG2, MEC1, PBN1, IKI1, SUR2, GUS1, SLT2, CSI1, DCS2, RPN1, YRF1-1, UBP11, SUP45, YGK3, TR(UCU)J1, HSM3, CUP9, GAL83, DBP8, TC(GCA)P1, ICL2, NMA1, CAF130, HPR1, PBS2, PPT1, MET18, RAD26, YPS1, CWH43, HSP60, SSA4, DIB1, SWI5, TM(CAU)J3, PHR1, REV7, MSE1, ROG1, ALG2, RPL13A, GAL1, RDH54, DIA4, PIB1, CAC2, AAH1, SIP1, ARG5,6, UBC6, RPS24B, APA1, STT3, SFA1, DOA1, NDE1, TQ(UUG)B, ARO7, SDL1, TE(UUC)C, PDR3, FAT1, HRR25, HSP82, CHL1, SMX2, ATG19, ELP2, CPR5, SUR4, PRE5, AAR2, GAT1, CHD1, RPL16B, SEF1, RKM2, SEC28, POP3, FKS1, ARG2, APS3, MRPS18, CSR2, RPL35A, RAD3, GDH1, PNG1, VPS24, ALG12, MEF1, XYL2, TOM1, OAZ1, USA1, SAK1, RPO21, PCM1, GIM3, YBR033W, PUS1, MRK1, CDC43, HUB1, PGM2, MCD4, PRP18, FAA2, ADD37, IXR1, HYS2, PHO8, RPA49, RPL8A, CAK1, KRS1, TFG1, ATG18, RPS0B, GLO2, SEN2, GAT2, FPR3, PET112, BUD21, AMD1, YJR107W, SEC13, MET28, ATG1, PSK1, SNR19, ATG3, RPL6B, AIP1, CDC39, TR(UCU)K, YOX1, RPC40, VAS1, HPT1, SSP1, ISY1, MSM1, TH(GUG)E1, ADE8, TAD3, LYS2, SMX3, LYS12, TN(GUU)K, PTP2, DOT6, CTK2, APC1, UBX3, HOR2, INO4, LSM3, CDC31, ATG7, TG(GCC)M, INM1, CCT4, MLP1, TR(CCG)L, GLY1, GPI12, TP(AGG)C, TPA1, KTR4, SUC2, MAL11, ORC4, GAL3, WRS1, ERG2, YLR126C, ERG11, CRC1, IST3, YPT1, RPG1, RPT3, MSH2, BNA4,

				CRH1, MAL33, DPL1, RPL5, MDY2, MSD1, TPK2, MKT1, TGL2, MPA43, TS(AGA)J, DED1, FLC1, FCF2, YFR006W, MET31, TG(GCC)C, MRPL25, SDS3, SME1, COQ1, TEL2, SMM1, PUS7, IMP1, YNR071C, GLO4, POL5, NHP10, TR(UCU)J2, SAM4, SKI2, ERG5, UTR1, PAC10, YPL144W, CDC55, HIS3, DOG2, SSL1, TT(UGU)P, TG(UCC)O, STB5, UGA2, TPS3, SRL2, ADE6, KAR2, ATP8, TRM12, SUT1, PEX11, SSN2, UTP5, PCL5, HMS1, RPL9B, ALG14, ARG81, UTP21, PRS2, SWD3, NDI1, YSF3, DAL80, JEM1, BET4, BGL2, RPS24A, KAP123, MTQ1, ERG3, NMD5, ALG6, MSC1, RDS3, HOG1, SNF11, RPL7B, SLS1, RPA135, LSM4, CFT1, YRF1-3, RGT1, RPL22B, CCA1, CWC23, RET2, EDC2, NRM1, ADH4, SIR1, IRE1, FSP2, IZH1, CTI6, SPT7, UPC2, TEP1, HNT1, FAA3, RPT4, VRG4, LSM5, MNN1, LEU1, MRPL20, TKL1, MRPL40, SHU2, MMS2, CCE1, TSC3, TQ(UUG)E1, AXL1, DBF4, STE7, YRF1-2, TEL1, RSC30, SER33, CDC36, PET122, CLB6, SEC27, DER1, DBP5, NAS2, TUB3, PAC2, TR(UCU)M2, NOC4, PGS1, SCO1, BRR2, EFT2, CHS6, TMA20, PRD1, TG(GCC)P2, ULP1, TR(ACG)D, MAK3, IOC4, FIP1, RSC58, RPL35B, MRPL37, NOP1, KTR3, NUP170, MCK1, PHO85, NTG2, MNT3, TKL2, SCEI, CCT6, SWP1, TE(UUC)P, PRP28, RRP5, SSL2, TS(AGA)M, MRPL27, YPC1, RPO31, HAT1, YRM1, UBA1, SRB7, PRI2, PER1, CDC1, KAP95, RAD1, MSS116, TV(CAC)D, IDH2, HMRA1, PRP8, URA7, YNR063W, YRF1-7, RPS27B, TA(AGC)K2, PHB2, SNM1, MND2, YKL161C, TA(UGC)A, GCD6, LAG1, PEX13, CBC2, TAF14, TAL1, RNT1, IMD4, ATG5, UTP15, UBP3, IDI1, STO1, MOD5, VMA22, HPA3, MSS51, SHC1, MTG2, ORC2, KNH1, HAL9, SUE1, ARO2, CDC21, APN1, ROT2, TOP3, RRB1, INO80, SEN54, SPT3, RML2, DGA1, HHT1, SCW4, SKI6, VPS15, TAF2, FYV6, ALD2, PHS1, HIS5, APL3, MDM20, GIS1, UBP14, VPS25, HRT3, RCE1, SEC12, VMA10, CPR3, YAP3, KCC4, FDH1, SPT4, PFS2, LPD1, HSP26, URB2, DAL4, SEC23, MRPL39, PBP1, OSH3, MXR1, GAS4, CIN5, TS(AGA)D3, OMA1, YDR520C, SWH1, ADE3, YNK1, PTH1, THP1, MAF1, SUI3, SUA7, NPR1, DUS3, ISM1, RPT6, SEC59, CSH1, PMT6, SLF1, MSS1, YOR059C, ICL1, PRS3, SNU114, WTM2, GCV2, ACN9, RSM10, MTG1, ERB1, ALD3, SUI1, DAP2, PCL6, ADH3, HAS1, MRPL8, GAL4, AI4, GRE3, CAR1, RPA190, DAL2, THR1, PCL8, RPS29A, MIS1, PIM1, MNS1, GIM4, YJL103C, DBF20, ALG7, TYR1, KIN2, TV(UAC)B, RNR1, PFK2, YPS3, HHT2, THP2, PTH2, YLR345W, MOB1, NUP145, DDI1, ARX1, PSD2, PRR1, DCS1,

				FMT1, RUB1, YSP3, TRE1, NPT1, MAG1, TAF10, TAZ1, PUF2, ADY3, RAD57, HYP2, TQ(UUG)C, TN(GUU)C, SGN1, CDC4, SEC53, NPL6, LGE1, FPR4, ADD66, GCD1, HSP10, GPI16, SWF1, YNL247W, RPB7, NTH1, PKR1, UTP22, FZF1, OST3, COX20, YRR1, SPT10, SOL4, RIM8, AAT2, TG(GCC)J2, ELP3, SHY1, TG(GCC)O2, EPS1, PKH1, PET111, CIN2, MTF1, ATH1, TE(UUC)E1, HAM1, UBC8, CCT5, ASN2, YPR118W, DLS1, MSI1, YLL054C, CDC6, DPM1, SIZ1, RAD59, SLC1, DAL82, TE(UUC)M, SNR58, GSC2, AAP1, TRF5, YHR044C, NOG1, TR(UCU)D, NAM2, RPF1, MDM30, RAD34, SMD3, YTA7, RPL24B, BRR1, DAL7, TS(AGA)D1, ALG9, MED2, IMP2, YOR283W, GUK1, PPH3, FAS2, TUB1, TE(UUC)B, DPS1, MET10, TS(UGA)P, HIS2, APT1, UMP1, SWI1, ILV1, BFR1, MAK10, PFA3, DEG1, MIG3, RME1, MET2, SLM5, AZF1, ASH1, YDJ1, ACA1, CDC26, HOM2, CNS1, LIP1, GAL2, RFC3, WSS1, EFT1, TG(CCC)O, ESS1, HXK1, DCN1, UBC1, YCK2, GPI14, HAP2, ZWF1, PRT1, MKC7, AI2, TR(UCU)M1, POS5, ECI1, SKM1, ULA1, YLR281C, EHD3, ESF1, SWD1, PRO1, STB2, NAT2, CTF18, RRF1, KTR2, MPD1, MRPL11, TQ(UUG)E2, ALG3, PRK1, TA(UGC)O, AHA1, MRP51, YPS5, ASP1, CDC2, REC107, DPB2, HIS4, PCL7, YHR113W, VPS74, NRD1, SLX8, XBP1, PFK1, TH(GUG)K, ALG5, SLM6, TAF6, YBR139W, AEP2, NCS2, ACB1, TIF6, HOM6, VMA21, STR3, GND1, YDR341C, EMG1, SGF11, THR4, ASK10, YFR055W, TFB1, RPL27A, RPA43, TL(GAG)G, CAT8, SWR1, HAP1, NRG1, TCM62, DUT1, ALG1, SOM1, AAT1, NDE2, TS(UGA)E, RPL8B, RPL18A, SCH9, RET3, SGA1, VHS1, HST4, PFK27, RDS1, ECM29, CLB5, YCS4, YMR31, RPN4, RPS16B, RAD28, TQ(UUG)L, MRPL50, ABD1, RPS30B, MSH4, YDR415C, NMD2, SLI15, NCA2, IST1, RPA14, RFA2, SNO1, COX12, RGR1, HCH1, URA6, UBC9, SRL3, DTD1, RRP9, ADH2, SHM2, TSC10, DIM1, SRN2, LRP1, UTP20, RAP1, NPY1, TPP1, ARG3, YFH1, TE(UUC)K, MRS1, MRM1, ARG80, FLO8, IPI3, THS1, SRP72, KTR6, FRS2
nitrogen compound metabolic process	134 out of 1943 genes, 6.9%	285 out of 6348 genes, 4.5%	2.92e-06	WRS1, ADH3, YLR126C, MSE1, GCN4, ARO4, DAL3, EHD3, DYS1, AGX1, DIA4, CAR1, PRO1, DAL2, THR1, ILS1, LEU2, MIS1, MSD1, ARG5,6, SFA1, ARO7, SDL1, UGA3, TYR1, MET31, ASP1, MST1, MET14, LYS1, HIS4, IDH2, URA7, SAM4, ATO3, FMT1, MHT1, HIS3, ARG2, UGA2, HOM6, LEU4, ADE6, STR3, YDR341C, GDH1, THR4, YFR055W, URA8, ARG81, ILV2, SER3, ILV3, YNL247W, ARO2, DAL80, HOM3, URA2, MSW1,

				PRO2, YIL165C, TYS1, AAT2, LEU9, AAT1, MSF1, CAR2, ARO1, YMR085W, ASN2, YPR118W, KRS1, IDH1, YHR020W, ADY2, ALD2, NIT3, HIS5, DAL82, ACO1, SER1, MET28, NAM2, ARO3, ADH4, GAD1, URE2, DAL81, GLT1, ARG8, LPD1, DAL4, DAL7, SHM1, IDP1, FMS1, PPH3, ADE3, VAS1, GUS1, LEU1, SNO1, DPS1, MET10, ILV5, TRP5, MSM1, HIS2, DTD1, TSC3, ILV1, NIT1, ISM1, LYS2, MEP2, LYS12, ADH2, SHM2, TSC10, MET2, SER33, ICL2, SLM5, MET18, DUR1,2, HOM2, ARG3, GLY1, GCV2, ARG80, ARG1, THS1, ALD3, FRS2
amine metabolic process	124 out of 1943 genes, 6.4%	261 out of 6348 genes, 4.1%	5.46e-06	WRS1, ADH3, YLR126C, MSE1, GCN4, MSF1, ARO4, DAL3, CAR2, EHD3, ARO1, DYS1, YMR085W, KRS1, YPR118W, ASN2, AGX1, DIA4, CAR1, PRO1, DAL2, IDH1, THR1, YHR020W, ILS1, LEU2, MIS1, ALD2, MSD1, HIS5, ARG5,6, DAL82, SFA1, ARO7, ACO1, SDL1, SER1, UGA3, ADH4, ARO3, NAM2, MET28, TYR1, GAD1, MET31, ASP1, GLT1, ARG8, LPD1, MST1, MET14, LYS1, HIS4, DAL4, DAL7, IDH2, IDP1, SHM1, FMS1, URA7, SAM4, ADE3, FMT1, VAS1, GUS1, MHT1, LEU1, HIS3, SNO1, MET10, DPS1, ILV5, TRP5, MSM1, ARG2, HIS2, DTD1, UGA2, HOM6, LEU4, TSC3, ILV1, ADE6, LYS2, ISM1, STR3, YDR341C, LYS12, ADH2, GDH1, TSC10, SHM2, THR4, MET2, SER33, YFR055W, ICL2, URA8, SLM5, MET18, ARG81, DUR1,2, ILV2, SER3, HOM2, ARG3, ILV3, YNL247W, ARO2, GLY1, ARG80, HOM3, GCV2, ARG1, URA2, MSW1, THS1, PRO2, TYS1, AAT2, ALD3, LEU9, FRS2, AAT1
amino acid metabolic process	114 out of 1943 genes, 5.9%	237 out of 6348 genes, 3.7%	9.85e-06	WRS1, ADH3, YLR126C, MSE1, GCN4, MSF1, ARO4, CAR2, EHD3, ARO1, YMR085W, KRS1, YPR118W, ASN2, AGX1, DIA4, CAR1, PRO1, IDH1, THR1, YHR020W, ILS1, LEU2, MIS1, ALD2, MSD1, HIS5, ARG5,6, SFA1, ARO7, ACO1, SDL1, SER1, ADH4, ARO3, NAM2, MET28, TYR1, GAD1, MET31, ASP1, GLT1, ARG8, LPD1, MST1, MET14, LYS1, HIS4, IDH2, IDP1, SHM1, URA7, SAM4, ADE3, FMT1, VAS1, GUS1, MHT1, LEU1, HIS3, SNO1, MET10, DPS1, ILV5, TRP5, MSM1, ARG2, HIS2, DTD1, UGA2, HOM6, LEU4, ILV1, ADE6, LYS2, ISM1, STR3, YDR341C, LYS12, ADH2, GDH1, SHM2, THR4, MET2, SER33, YFR055W, ICL2, URA8, SLM5, MET18, ARG81, DUR1,2, ILV2, SER3, HOM2, ARG3, ILV3, YNL247W, ARO2, GLY1, ARG80, HOM3, GCV2, ARG1, URA2, MSW1, THS1, PRO2, TYS1, AAT2, ALD3, LEU9, FRS2, AAT1
amino acid and derivative	117 out of 1943	254 out of 6348	0.00013	WRS1, ADH3, YLR126C, MSE1, GCN4, MSF1, ARO4, CAR2, EHD3, ARO1, DYS1, YMR085W,

metabolic process	genes, 6.0%	genes, 4.0%		KRS1, YPR118W, ASN2, AGX1, DIA4, CAR1, PRO1, IDH1, THR1, YHR020W, ILS1, LEU2, MIS1, ALD2, MSD1, HIS5, ARG5,6, SFA1, ARO7, ACO1, SDL1, SER1, UGA3, ADH4, ARO3, NAM2, MET28, TYR1, GAD1, MET31, ASP1, GLT1, ARG8, LPD1, MST1, MET14, LYS1, HIS4, IDH2, IDP1, SHM1, FMS1, URA7, SAM4, ADE3, FMT1, VAS1, GUS1, MHT1, LEU1, HIS3, SNO1, MET10, DPS1, ILV5, TRP5, MSM1, ARG2, HIS2, DTD1, UGA2, HOM6, LEU4, ILV1, ADE6, LYS2, ISM1, STR3, YDR341C, LYS12, ADH2, GDH1, SHM2, THR4, MET2, SER33, YFR055W, ICL2, URA8, SLM5, MET18, ARG81, DUR1,2, ILV2, SER3, HOM2, ARG3, ILV3, YNL247W, ARO2, GLY1, ARG80, HOM3, GCV2, ARG1, URA2, MSW1, THS1, PRO2, TYS1, AAT2, ALD3, LEU9, FRS2, AAT1
transport	427 out of 1943 genes, 22.0%	1165 out of 6348 genes, 18.4%	0.00084	SNX3, ATG2, MTM1, CRC1, YCF1, YPT1, IST3, DID2, PDR5, GET1, BCH1, PSE1, ITR1, LOS1, AST1, ERV29, AUS1, ERP1, YPR004C, DNF1, YKE4, USO1, YPT52, BUD7, GRX4, VAM3, YDL119C, MRH1, TPC1, TLG2, ENB1, ARN1, FLC1, KIN2, OPT1, YPT32, PEX22, SNF3, QCR8, THP2, ITR2, NUP145, CDC42, SEC14, IMP1, DDI1, HUT1, TAT2, YGR207C, ATO3, NEO1, SEC11, OSH6, SSC1, NDC1, HSP78, FAB1, SEC16, KAP122, ERP3, ATG11, BUD13, COR1, ATP8, LSG1, KAR2, AVT1, TOM20, SDH4, ERD1, SUT1, ATP3, AVT7, YIP5, SEC61, LOC1, YOR071C, SEC53, NPL6, ENT1, SDA1, ATP2, HXT9, KAP120, SEC24, GYP6, DOA4, MUP1, YPR157W, FZF1, RNA1, CAN1, YRR1, YMC2, VAM7, COY1, LSP1, DAL5, MDL1, ELP3, KAP123, ENA5, PHO84, VAM6, PKH1, COP1, SAR1, CDC50, PEX10, ERG3, NMD5, MSB3, YIF1, PEP12, ERP2, ENA1, NUP192, CLC1, AQY2, SNF7, YIA6, SIW14, LST7, VPS20, RSB1, YCR023C, RET2, NCE101, RER2, SEC31, AIR1, FAA4, LST8, DRS2, NOG1, ZRG17, PTR2, TIM9, TOM70, SUL1, FIT1, SMF1, TPO2, YHB1, KIP1, YPR003C, AVT2, IMP2, SFB3, YOR1, GUK1, PGA3, LST4, ODC2, COT1, ERV41, SEC66, FRE3, VRG4, LHS1, TUB1, GOT1, MET10, MST28, RGT2, BAP2, SFB2, VAC8, NAB2, YRB2, MEP2, GNP1, FUI1, HXT13, PEP7, KAP104, RLI1, MSN5, HXT12, HPR1, MST27, POM152, PBS2, ENT5, SIT1, HXT2, SEC27, CTR3, DER1, ECM38, DBP5, FEN2, YDJ1, TUB3, FCY2, PIC2, YDR338C, GAL2, ECM27, DIC1, SCO1, HSP60, SSA4, ATG8, ATG26, CHS6, VTH1, HXK1, BRE4, UBC1, YCK2, AAC3, YBR220C, RHO3, ENA2, NUP100, IVY1, NUP170, SUT2, YRO2, NFT1, GAL1, HOL1, SNX41, SSU1, ISA1, TIM21, VTH2, SSO2, FRE2, SEC39, ZRC1,

				SSL2, HXT5, VTA1, YLH47, GDI1, DAN1, MTR2, VPS38, HXT11, COG8, EMP24, NHA1, FAT1, SYS1, HRR25, ODC1, YRM1, GOS1, STF2, KAP95, GTR2, AGP1, MFT1, ATG19, SEC15, ELP2, VPS74, USE1, FPS1, MSS2, SUR4, MVB12, PXA2, TPO3, DNF2, COX2, YOR271C, YPR011C, PEX12, RIM2, PEX13, MSP1, YKR104W, CBC2, SEC28, FKS1, APS3, VTC4, ACB1, PMP2, TIF6, SLY41, ALP1, ATG5, STO1, THR4, PIL1, VPS24, HXT16, ARF3, SSP120, TOM1, GCS1, SGE1, BSP1, ENT4, SED4, TRS120, AVT4, GRX3, ERF2, CYC7, SRM1, YMR118C, GRX5, YVC1, FRE8, NOC2, YIL171W, TOM6, SAC3, YMR221C, TAT1, MCD4, GBP2, QCR9, YDR061W, RET3, OAC1, PMC1, FCY22, VPS15, AZR1, ERV15, MID1, ATG18, STV1, SXM1, ADY2, LEM3, YOL163W, SEC17, COX17, PHS1, SEC18, COX1, APL3, BST1, SDS24, NUP84, SEC13, VPS25, TRS130, MRL1, GAP1, BAP3, SEC12, ATG17, VMA10, APQ12, PCA1, ATG1, NIP1, TVP15, ARH1, DTR1, QDR2, VHT1, CCC2, SEC23, ATG3, DAL4, SSA2, YHC3, VBA1, VPS52, PHO89, OSH3, JEN1, HXT15, TOM40, MIA40, YIP1, SWH1, FRE4, SEC9, VID24, YMR171C, VPS73, MCH1, YAH1, IST1, VMA6, FET5, YOR378W, GEA2, THP1, HSE1, MDJ2, NUP157, NOP53, YIL166C, YKT6, MCH5, SBE22, STF1, YLL053C, BPH1, FUR4, NUP85, NUS1, LTE1, YSC84, POM34, COS10, SRN2, YEA6, CTP1, SEC21, YJL045W, ATG7, CDC31, YLR004C, YFH1, MLP1, ARN2, VCX1, COX18, MAL11, YFL054C, SRP72, AFT1, GET3, PBI2, SAM3
establishment of localization	430 out of 1943 genes, 22.1%	1179 out of 6348 genes, 18.6%	0.00146	SNX3, ATG2, MTM1, CRC1, YCF1, YPT1, IST3, DID2, PDR5, GET1, BCH1, PSE1, ITR1, LOS1, AST1, ERV29, AUS1, ERP1, YPR004C, DNF1, YKE4, USO1, YPT52, BUD7, GRX4, VAM3, YDL119C, MRH1, TPC1, TLG2, ENB1, DMA1, ARN1, FLC1, KIN2, OPT1, YPT32, PEX22, SNF3, QCR8, THP2, ITR2, NUP145, CDC42, SEC14, IMP1, DDI1, HUT1, TAT2, YGR207C, ATO3, NEO1, SEC11, OSH6, SSC1, NDC1, HSP78, FAB1, SEC16, KAP122, ERP3, ATG11, BUD13, COR1, ATP8, LSG1, KAR2, AVT1, TOM20, SDH4, ERD1, SUT1, ATP3, AVT7, YIP5, SEC61, LOC1, YOR071C, SEC53, NPL6, ENT1, SDA1, ATP2, HXT9, KAP120, SEC24, GYP6, DOA4, MUP1, YPR157W, FZF1, RNA1, CAN1, YRR1, YMC2, VAM7, COY1, LSP1, DAL5, MDL1, ELP3, KAP123, ENA5, PHO84, VAM6, PKH1, COP1, SAR1, CDC50, PEX10, ERG3, NMD5, MSB3, YIF1, PEP12, ERP2, ENA1, NUP192, CLC1, AQY2, SNF7, YIA6, SIW14, LST7, VPS20, RSB1, YCR023C, RET2, NCE101, RER2, SEC31, AIR1, FAA4, LST8, DRS2, NOG1,

				ZRG17, PTR2, TIM9, TOM70, SUL1, FIT1, SMF1, TPO2, YHB1, KIP1, YPR003C, AVT2, IMP2, SFB3, YOR1, GUK1, PGA3, LST4, ODC2, COT1, ERV41, SEC66, FRE3, VRG4, LHS1, TUB1, GOT1, MET10, MST28, RGT2, BAP2, SFB2, VAC8, NAB2, YRB2, MEP2, GNP1, FUI1, HXT13, PEP7, KAP104, RLI1, MSN5, HXT12, HPR1, MST27, POM152, PBS2, ENT5, SIT1, HXT2, SEC27, CTR3, DER1, ECM38, DBP5, FEN2, YDJ1, TUB3, FCY2, PIC2, YDR338C, GAL2, ECM27, DIC1, SCO1, HSP60, SSA4, ATG8, ATG26, CHS6, VTH1, HXK1, BRE4, UBC1, YCK2, AAC3, YBR220C, RHO3, ENA2, NUP100, IVY1, NUP170, SUT2, YRO2, NFT1, GAL1, HOL1, SNX41, SSU1, ISA1, TIM21, VTH2, SSO2, FRE2, SEC39, ZRC1, SSL2, HXT5, VTA1, YLH47, GDI1, DAN1, MTR2, VPS38, HXT11, COG8, EMP24, NHA1, FAT1, SYS1, HRR25, ODC1, YRM1, GOS1, STF2, KAP95, GTR2, AGP1, MFT1, ATG19, SEC15, ELP2, VPS74, USE1, FPS1, MSS2, SUR4, MVB12, PXA2, TPO3, DNF2, COX2, YOR271C, YPR011C, PEX12, RIM2, PEX13, MSP1, YKR104W, CBC2, SEC28, FKS1, APS3, VTC4, ACB1, PMP2, TIF6, SLY41, ALP1, ATG5, STO1, THR4, PIL1, VPS24, HXT16, ARF3, SSP120, TOM1, GCS1, SGE1, BSP1, ENT4, SED4, TRS120, AVT4, GRX3, ERF2, CYC7, SRM1, YMR118C, GRX5, YVC1, FRE8, NOC2, YIL171W, TOM6, SAC3, YMR221C, TAT1, MCD4, GBP2, QCR9, YDR061W, RET3, OAC1, PMC1, FCY22, VPS15, AZR1, ERV15, MID1, ATG18, STV1, SXM1, ADY2, LEM3, YOL163W, SEC17, COX17, PHS1, SEC18, COX1, APL3, BST1, SDS24, NUP84, SEC13, VPS25, TRS130, MRL1, GAP1, BAP3, SEC12, ATG17, VMA10, APQ12, PCA1, ATG1, NIP1, TVP15, ARH1, DTR1, QDR2, VHT1, CCC2, SEC23, ATG3, DAL4, SSA2, YHC3, VBA1, VPS52, PHO89, OSH3, JEN1, HXT15, TOM40, MIA40, YIP1, SWH1, FRE4, SEC9, VID24, YMR171C, VPS73, MCH1, YAH1, IST1, VMA6, FET5, RFA2, YOR378W, GEA2, THP1, HSE1, MDJ2, NUP157, NOP53, YIL166C, YKT6, MCH5, SBE22, STF1, YLL053C, BPH1, FUR4, NUP85, NUS1, LTE1, YSC84, POM34, COS10, SRN2, YEA6, CTP1, SEC21, YJL045W, ATG7, CDC31, YLR004C, YFH1, MLP1, ARN2, VCX1, COX18, DMA2, MAL11, YFL054C, SRP72, AFT1, GET3, PBI2, SAM3
localization	444 out of 1943 genes, 22.9%	1222 out of 6348 genes, 19.3%	0.00150	SNX3, ATG2, CRN1, MTM1, CRC1, YCF1, YPT1, IST3, DID2, PDR5, GET1, BCH1, PSE1, ITR1, LOS1, AST1, ERV29, AUS1, ERP1, YPR004C, DNF1, ABF2, YKE4, ARP2, USO1, YPT52, BUD7, GRX4, VAM3, MDM31, YDL119C, MRH1, TPC1, TLG2, ENB1, DMA1, ARN1, FLC1, KIN2, OPT1, YPT32, PEX22,

				SNF3, QCR8, THP2, ITR2, NUP145, CDC42, SEC14, IMP1, DDI1, HUT1, TAT2, YGR207C, ATO3, PDS1, NEO1, SEC11, OSH6, SSC1, NDC1, HSP78, FAB1, SEC16, KAP122, ERP3, ATG11, BUD13, COR1, ATP8, KAR2, LSG1, AVT1, TOM20, SDH4, ERD1, SUT1, ATP3, AVT7, YIP5, SEC61, LOC1, YOR071C, SEC53, NPL6, ENT1, SDA1, ATP2, HXT9, KAP120, SEC24, GYP6, DOA4, MUP1, YPR157W, FZF1, RNA1, CAN1, YRR1, YMC2, VAM7, COY1, LSP1, DAL5, MDL1, ELP3, KAP123, ENA5, EPS1, PHO84, VAM6, PKH1, COP1, SAR1, CDC50, PEX10, ERG3, NMD5, MSB3, YIF1, PEP12, ERP2, ENA1, NUP192, CLC1, AQY2, SNF7, YIA6, SIW14, LST7, VPS20, RSB1, YCR023C, RET2, NCE101, RER2, SEC31, AIR1, FAA4, LST8, DRS2, ARC15, NOG1, ZRG17, PTR2, TIM9, TOM70, SUL1, URE2, FIT1, SMF1, TPO2, YHB1, KIP1, YPR003C, AVT2, IMP2, SFB3, YOR1, GUK1, PGA3, LST4, ODC2, COT1, ERV41, SEC66, FRE3, VRG4, LHS1, TUB1, GOT1, MET10, MST28, RGT2, BAP2, SFB2, VAC8, NAB2, YRB2, MEP2, GNP1, FUI1, HXT13, PEP7, KAP104, RLI1, MSN5, HXT12, HPR1, MST27, POM152, PBS2, ENT5, SIT1, HXT2, SEC27, SCP160, CTR3, DER1, ECM38, DBP5, FEN2, YDJ1, TUB3, FCY2, PIC2, YDR338C, GAL2, ECM27, DIC1, SCO1, HSP60, SSA4, ATG8, ATG26, CHS6, VTH1, HXK1, BRE4, UBC1, YCK2, AAC3, YBR220C, RHO3, ENA2, NUP100, IVY1, NUP170, SUT2, YRO2, NFT1, GAL1, HOL1, SNX41, SSU1, ISA1, TIM21, VTH2, SSO2, FRE2, SEC39, BUL1, ZRC1, SSL2, HXT5, VTA1, YLH47, GDI1, DAN1, MTR2, VPS38, HXT11, EMP24, COG8, NHA1, FAT1, SYS1, HRR25, ODC1, YRM1, GOS1, STF2, KAP95, GTR2, AGP1, HEK2, MFT1, SHR5, ATG19, SEC15, ELP2, VPS74, USE1, FPS1, MSS2, SUR4, MVB12, PXA2, TPO3, DNF2, COX2, YOR271C, YPR011C, PHB2, PEX12, RIM2, PEX13, MSP1, YKR104W, CBC2, SEC28, FKS1, APS3, VTC4, ACB1, PMP2, TIF6, SLY41, ALP1, ATG5, STO1, THR4, PIL1, VPS24, HXT16, ARF3, SSP120, TOM1, GCS1, SGE1, BSP1, ENT4, SED4, TRS120, AVT4, GRX3, ERF2, CYC7, SRM1, YMR118C, GRX5, YVC1, FRE8, NOC2, YIL171W, TOM6, SAC3, YMR221C, TAT1, MCD4, GBP2, QCR9, YDR061W, RET3, OAC1, PMC1, FCY22, VPS15, AZR1, ERV15, MID1, ATG18, STV1, SXM1, ADY2, LEM3, YOL163W, SEC17, COX17, PHS1, SEC18, COX1, APL3, BST1, SDS24, MDM20, NUP84, SEC13, VPS25, TRS130, MRL1, GAP1, BAP3, SEC12, ATG17, VMA10, APQ12, PCA1, ATG1, NIP1, TVP15, ARH1, DTR1, QDR2, VHT1, CCC2, SEC23, ATG3, DAL4, SSA2, YHC3, VBA1,

				VPS52, PHO89, OSH3, JEN1, HXT15, TOM40, MIA40, YIP1, SWH1, FRE4, SEC9, VID24, YMR171C, VPS73, MCH1, YAH1, IST1, VMA6, FET5, RFA2, YOR378W, GEA2, THP1, HSE1, MDJ2, NUP157, NOP53, YIL166C, YKT6, MCH5, SBE22, STF1, YLL053C, BPH1, FUR4, NUP85, NUS1, LTE1, YSC84, POM34, COS10, SRN2, YEA6, CTP1, SEC21, YJL045W, ATG7, CDC31, YLR004C, YFH1, ARN2, MLP1, VCX1, COX18, DMA2, MAL11, YFL054C, SRP72, AFT1, GET3, PBI2, SAM3
biosynthetic process	693 out of 1943 genes, 35.7%	1999 out of 6348 genes, 31.5%	0.00186	ERG2, WRS1, SOH1, ERG11, TR(UCU)E, TUF1, RPG1, GCN4, MAL33, BNA4, MSH2, BCH1, MUM2, RPL5, AGX1, RTT102, GCD10, MTO1, ECM31, MSD1, CLU1, MNT2, RPL13B, CBP6, TS(AGA)J, DED1, STD1, TG(GCC)C, MET31, MRPL25, SDS3, NTO1, TK(CUU)J, MET14, TEL2, TG(UCC)N, LYS1, COQ1, TS(AGA)D2, POL5, NHP10, TR(UCU)J2, SAM4, ERG5, UTR1, CDC55, HIS3, SSL1, CYR1, STB5, TG(UCC)O, TT(UGU)P, ADE6, VIP1, CSG2, ATP8, ERD1, SUT1, BDF1, SSN2, UTP5, GAL80, GCD7, RPL9B, HMS1, ALG14, TE(UUC)J, ATP2, RIB4, ARG81, PRS2, SGS1, RKI1, SOL3, CKS1, NDI1, DAL80, HOM3, BET4, MSW1, RPS24A, TIF4632, PET309, TQ(UUG)D3, MTQ1, CCL1, GSH2, HDA2, CAD1, ERG3, ALG6, RET1, HOG1, SNF11, RPL7B, YNL045W, SLS1, RPA135, CFT1, VHR1, IMD2, GLG1, MRP2, RGT1, RPL22B, GPI18, SPT20, SUP35, SWI6, PCK1, HEM13, NRM1, RER2, TG(GCC)B, TR(ACG)K, MDH2, ERG6, FAA4, TFB3, REF2, ADH4, SIR1, NDD1, GPI11, IRE1, TI(AAU)L1, ARG8, ZAP1, CTI6, TH(GUG)M, SPT7, UPC2, IDP1, SNF4, INO1, STE12, MRPL3, HNT1, FAA3, RPC37, VRG4, GLC8, MNN1, MRPL20, LEU1, PMT5, TKL1, MRPL40, TRP5, ILV5, MRP20, TSC3, RLM1, SRB2, TQ(UUG)E1, KTR7, DBF4, TEL1, RSC30, SER33, RLI1, CDC36, CDC7, PET122, CLB6, KTR1, DBP5, TR(UCU)M2, ERG25, PGS1, HEM3, GPI19, ATG26, EFT2, CHS6, TMA20, PRS4, TG(GCC)P2, TR(ACG)D, IOC4, RSC9, MRPL37, RPL35B, RSC58, KTR3, SUT2, ARO4, RTT106, SWI4, MNT3, TKL2, SWP1, TD(GUC)J1, TE(UUC)P, TG(CCC)D, SGF29, BIO3, SSL2, MOT3, TS(AGA)M, MRPL27, MNN4, RNH203, IPT1, RPO31, HEM4, YRM1, ERG9, SRB7, STF2, PRI2, PER1, RAD1, HEK2, MFT1, HCR1, TV(CAC)D, SHR5, RPS9B, ADE12, HMRA1, IDH2, URA7, YNR063W, TS(AGA)B, RPS27B, TA(AGC)K2, GCD6, RPL9A, TA(UGC)A, LAG1, TAF14, LAS21, TAL1, TA(UGC)L, RNT1, IMD4, LEU4, TR(UCU)B, ERG7, KRE6, TQ(UUG)D1, ATG5, UTP15, IDI1, MRPL44, RNR2, DCC1, MSS51, MTG2, SHC1, HTZ1, ILV2,

				SER3, ORC2, HAL9, KNH1, TS(AGA)A, ARO2, PMT2, CDC21, ERF2, PGI1, IMG2, RRN6, PRO2, TOP3, INO80, EAF5, YBR284W, SAC3, SPT3, CDS1, RML2, DGA1, YGR043C, TAF13, ARO1, TS(AGA)L, TAF2, EAF7, IDH1, SEN1, YHR020W, GCR1, ERG28, MNP1, PHS1, ALD2, HIS5, TE(CUC)D, GIS1, MNN9, SNZ3, ADE5,7, SER1, YOL054W, YAP3, FDH1, NIP1, PDX3, SPT4, LPD1, GDA1, BRF1, MRPL39, HKR1, SHM1, CIN5, BUR6, TS(AGA)D3, SWH1, YDR520C, ADE3, YNK1, YAH1, TSC13, PTH1, THP1, LRO1, MET7, TAF4, KRE5, MAF1, SUI3, SUA7, TT(AGU)J, ISM1, STF1, TM(CAU)J1, RPS0A, SEC59, YLR278C, KAE1, CSH1, PMT6, MSS1, RPL31B, PRS3, NUT2, TL(UAA)J, WTM2, ARG1, ACN9, RSM10, URA1, MTG1, AFT1, ALD3, SUI1, NCP1, ADH3, MRPL8, SIS2, SSU72, MRP10, GAL4, PMT1, RPA190, THR1, LEU2, ILS1, TRA1, RPS29A, MIS1, SLD5, MNS1, BUD7, MRPL33, NRG2, RPB9, YJL103C, ALG7, RPC82, PSK2, UGA3, MNN2, TYR1, TV(UAC)B, RNR1, TOS4, ESC8, HST2, COX15, MST1, THP2, CHS3, PTH2, HOS1, TQ(UUG)D2, PDR8, ORC5, PSD2, FMT1, MHT1, HAC1, RNR4, HEF3, RHR2, NSG1, MSH5, TS(AGA)E, NPT1, TAF10, ATP3, TAZ1, TQ(UUG)C, HYP2, TN(GUU)C, URA8, SEC53, NPL6, LGE1, GCD1, HUR1, GPI16, SWF1, ERG12, MED11, RPB7, YNL247W, ILV3, YER184C, FZF1, OST3, CWH41, YRR1, PHD1, SOL4, SPT8, URA2, HEM1, GPD1, ELP3, TYS1, TG(GCC)J2, PAN5, AAT2, GUT2, LEU9, EST2, TG(GCC)O2, PET111, MBF1, MTF1, WBP1, MSF1, TE(UUC)L, NSG2, TE(UUC)E1, HAM1, ASN2, YPR118W, DLS1, CDC5, YLL054C, RPI1, CDC6, DPM1, SLC1, DAL82, TE(UUC)M, GSC2, ACO1, TR(UCU)D, NAM2, ARO3, DAL81, GLT1, FUN12, TIF5, RPS30A, SRB8, RNR3, RPL24B, SCT1, TS(AGA)D1, ALG9, FMS1, PMT3, MED2, NUT1, GUK1, HMG2, FAS2, PBN1, IKI1, SUR2, GUS1, TE(UUC)B, DPS1, MET10, TS(UGA)P, HIS2, APT1, SWI1, SUP45, ILV1, TR(UCU)J1, SNZ2, HSM3, CUP9, PFA3, MIG3, RME1, MET2, TC(GCA)P1, NMA1, SLM5, CAF130, HPR1, AZF1, MET18, ECM38, ASH1, RIB5, ACA1, HOM2, LIP1, RFC3, CWH43, EFT1, TG(CCC)O, ESS1, SWI5, HAP2, GPI14, ZWF1, PRT1, TM(CAU)J3, AI2, TR(UCU)M1, POS5, MSE1, ALG2, RPL13A, YLR281C, PRO1, DIA4, HEM12, CTF18, RRF1, AAH1, RIB7, ARG5,6, KTR2, RPS24B, APA1, STT3, MRPL11, SFA1, NDE1, TQ(UUG)B, ARO7, SDL1, TE(UUC)C, TQ(UUG)E2, ALG3, PDR3, TA(UGC)O, MRP51, CDC2, DPB2, HIS4, ELP2, VPS74, NRD1, SUR4, XBP1, TH(GUG)K, ALG5, GAT1, CHD1,

				YPR172W, RPL16B, SEF1, TAF6, YBR139W, AEP2, FKS1, ARG2, MRPS18, TIF6, CSR2, HOM6, RPL35A, GND1, STR3, YDR341C, RAD3, GDH1, SGF11, THR4, RIB3, ASK10, RPL27A, YFR055W, TFB1, ALG12, RPA43, TL(GAG)G, CAT8, SWR1, MEF1, HAP1, TOM1, NRG1, SAK1, RPO21, PCM1, DUT1, ALG1, YBR033W, PAN6, CDC43, AAT1, NDE2, PGM2, YMR041C, TS(UGA)E, RPL8B, MCD4, RPL18A, FAA2, IXR1, HYS2, RPA49, RPL8A, KRS1, HST4, TFG1, RPS0B, RDS1, CLB5, GAT2, PET112, YMR31, THI4, AMD1, RPN4, RPS16B, MET28, COQ3, PSK1, TQ(UUG)L, MRPL50, RPL6B, ATG3, RPS30B, MSH4, CDC39, TR(UCU)K, YOX1, RPC40, IST1, HPT1, VAS1, RPA14, RFA2, SNO1, RGR1, MSM1, URA6, DTD1, TH(GUG)E1, ADE8, LYS2, LYS12, TN(GUU)K, ADH2, TSC10, DOT6, HOR2, INO4, RAP1, ATG7, TG(GCC)M, NPY1, ARG3, TE(UUC)K, TR(CCG)L, GLY1, GPI12, TP(AGG)C, ARG80, TPA1, KTR4, FLO8, THS1, LIP5, ORC4, KTR6, FRS2
organic acid metabolic process	155 out of 1943 genes, 8.0%	371 out of 6348 genes, 5.8%	0.00253	WRS1, ADH3, CRC1, YLR126C, EEB1, MSE1, GCN4, ECI1, ARO4, EHD3, DPL1, AGX1, DIA4, CAR1, PRO1, THR1, ILS1, LEU2, MIS1, BIO3, MSD1, ARG5,6, SFA1, ARO7, SDL1, UGA3, TYR1, FAT1, PDH1, MET31, ASP1, MST1, MET14, LYS1, HIS4, IDH2, URA7, GLO4, SUR4, SAM4, FMT1, MHT1, HIS3, ARG2, ACB1, DLD2, UGA2, HOM6, LEU4, ADE6, STR3, YDR341C, GDH1, PEX11, THR4, YFR055W, URA8, ARG81, ILV2, SER3, ILV3, YNL247W, ARO2, PGI1, HOM3, URA2, MSW1, PRO2, TYS1, AAT2, LEU9, AAT1, PDB1, YMR041C, RML2, FAA2, MSF1, YNL045W, CAR2, ARO1, YMR085W, KRS1, VHR1, ASN2, HST4, YPR118W, GLO1, IDH1, YHR020W, GLO2, ALD2, PCK1, HIS5, MDH2, PYK2, FAA4, ACO1, SER1, ADH4, MET28, NAM2, ARO3, GAD1, EHT1, FDH1, GLT1, PDX3, ARG8, LPD1, DAL7, SHM1, IDP1, FAA3, FAS2, ADE3, VAS1, TSC13, GUS1, LEU1, YNL274C, SNO1, DPS1, MET10, ILV5, TRP5, MSM1, HIS2, MET7, DTD1, ILV1, ISM1, LYS2, LYS12, PDA1, ADH2, SHM2, MET2, SER33, ICL2, SLM5, POT1, MET18, DUR1,2, ICL1, HOM2, ARG3, GLY1, GCV2, ARG80, ARG1, ACN9, THS1, LIP5, ALD3, FRS2
carboxylic acid metabolic process	155 out of 1943 genes, 8.0%	371 out of 6348 genes, 5.8%	0.00253	WRS1, ADH3, CRC1, YLR126C, EEB1, MSE1, GCN4, ECI1, ARO4, EHD3, DPL1, AGX1, DIA4, CAR1, PRO1, THR1, ILS1, LEU2, MIS1, BIO3, MSD1, ARG5,6, SFA1, ARO7, SDL1, UGA3, TYR1, FAT1, PDH1, MET31, ASP1, MST1, MET14, LYS1, HIS4, IDH2, URA7, GLO4, SUR4, SAM4, FMT1, MHT1, HIS3, ARG2, ACB1, DLD2, UGA2, HOM6, LEU4,

				ADE6, STR3, YDR341C, GDH1, PEX11, THR4, YFR055W, URA8, ARG81, ILV2, SER3, ILV3, YNL247W, ARO2, PGI1, HOM3, URA2, MSW1, PRO2, TYS1, AAT2, LEU9, AAT1, PDB1, YMR041C, RML2, FAA2, MSF1, YNL045W, CAR2, ARO1, YMR085W, KRS1, VHR1, ASN2, HST4, YPR118W, GLO1, IDH1, YHR020W, GLO2, ALD2, PCK1, HIS5, MDH2, PYK2, FAA4, ACO1, SER1, ADH4, MET28, NAM2, ARO3, GAD1, EHT1, FDH1, GLT1, PDX3, ARG8, LPD1, DAL7, SHM1, IDP1, FAA3, FAS2, ADE3, VAS1, TSC13, GUS1, LEU1, YNL274C, SNO1, DPS1, MET10, ILV5, TRP5, MSM1, HIS2, MET7, DTD1, ILV1, ISM1, LYS2, LYS12, PDA1, ADH2, SHM2, MET2, SER33, ICL2, SLM5, POT1, MET18, DUR1,2, ICL1, HOM2, ARG3, GLY1, GCV2, ARG80, ARG1, ACN9, THS1, LIP5, ALD3, FRS2
amino acid biosynthetic process	64 out of 1943 genes, 3.3%	129 out of 6348 genes, 2.0%	0.00578	ADE3, GCN4, MHT1, LEU1, HIS3, MET10, ARO4, TRP5, ILV5, ARG2, HIS2, ARO1, YPR118W, ASN2, AGX1, PRO1, HOM6, LEU4, IDH1, ILV1, THR1, LYS2, STR3, LEU2, MIS1, LYS12, GDH1, ALD2, THR4, HIS5, ARG5,6, MET2, SER33, YFR055W, ARO7, ACO1, SER1, ARO3, TYR1, MET28, ILV2, SER3, HOM2, ARG3, ILV3, GLT1, ARO2, ARG8, LPD1, GLY1, MET14, LYS1, HIS4, HOM3, ARG1, IDH2, IDP1, SHM1, PRO2, SAM4, AAT2, ALD3, LEU9, AAT1
response to stimulus	342 out of 1943 genes, 17.6%	925 out of 6348 genes, 14.6%	0.00632	TDP1, HOF1, ATG2, SOH1, YCF1, SIS2, GCN4, TIR1, PDR5, MSH2, GET1, URM1, GAL4, GRE3, DPL1, TAP42, TRA1, MDY2, PIM1, CMP2, AGA1, SVF1, MTO1, GRX4, RPB9, MKT1, MFA1, MTL1, ATF2, STD1, AGA2, IRS4, SNF3, HHT2, MET14, CDC42, CCP1, GLO4, STE18, PDR8, UBC13, PDS1, PRR1, SKI2, SSC1, HSP78, FAB1, YOR338W, DOG2, SSL1, HAC1, KAP122, STB5, RHR2, AFG1, TPS3, UGA2, ATC1, MSH5, CSG2, KAR2, MAG1, PRX1, SEY1, BDF1, RAD57, GAL80, NPL6, KIN82, SGS1, CRT10, NTH1, MID2, YRR1, HOM3, SPT10, CUP1-2, LSP1, HOR7, GPD1, YOL019W, RRD1, GSH2, CAD1, RDS3, AAD6, TIR2, HOG1, ATH1, GRE1, HAM1, ENA1, TOS1, MSI1, GLO1, SIW14, RSB1, SPT20, YCR023C, RAS2, HSP104, RAD59, RIM20, TRF5, SGT2, ACO1, TFB3, ARO3, PTC6, URE2, IRE1, RAD34, IZH1, RAD50, YHB1, STE12, YDL038C, CSL4, YOR1, PPH3, HAT2, MEC1, RAD16, LHS1, SLT2, CSI1, RMI1, MET10, SSD1, SHU2, RGT2, CDC10, MMS2, UMP1, YGK3, RLM1, HSM3, DIN7, STE7, ZTA1, MIG3, TEL1, RSC30, PRM2, CDC36, HPR1, AZF1, PBS2, MET18, MF(ALPHA)1, WSC2, SCP160, RAD26, YDJ1, FMC1, BDS1, HOM2, YLR046C, YLR247C, SSK1, YDR338C, RFC3, SCO1,

				WSS1, HSP60, ATG8, SSA4, HUG1, HTB1, UBC1, YCK2, ZWF1, PHR1, RSC9, REV7, MCK1, POS5, ARO4, GAL1, PHO85, HSP42, SFK1, DAK2, NTG2, RDH54, SMC6, ZRC1, CAC2, SSL2, MNN4, PSY4, DOA1, DAN1, MFA2, HAT1, SDL1, BEM4, NHA1, PDR3, AHA1, HRR25, YRM1, GOS1, IES4, HSP82, CDC2, STF2, PRI2, CDC1, GTR2, RAD1, MF(ALPHA)2, DPB2, TIR3, VPS74, SLX8, XBP1, FPS1, SLH1, CUP1-1, FAR1, SLM6, YPD1, PAN2, HOM6, AAD4, GND1, ATG5, UBP3, RAD3, SPI1, DFM1, YBR014C, ASK10, PIL1, SIP5, TFB1, DCC1, AAD3, SGE1, NRG1, HAL9, TIR4, ARO2, STE4, GRX3, RRI2, APN1, TCM62, SRM1, GRX5, MRK1, KOG1, YKU80, HPR5, INO80, EAF5, HUB1, SAC3, RGD1, HSP31, YGL220W, IXR1, HYS2, HHT1, SCH9, SKI6, VPS15, AZR1, CLN2, ATG18, EAF7, SKI3, GLO2, RDS1, FYV6, PRM5, TRM2, PRB1, AAD15, NUP84, RPN4, UBP14, HSP12, VPS25, RCE1, MET28, SSE2, TRR2, ATG17, GAD1, ATG1, RAD28, PRM7, SOD1, QDR2, SIP18, HSP26, ATG3, SSA2, AIP1, HKR1, RRI1, MSH4, IRA2, BNR1, CDC39, MXR1, CIN5, MAM33, ORM1, SVS1, RFA2, GPX1, THP1, YIR041W, MDJ2, HCH1, OPY2, TOR1, BPH1, PTP2, LTE1, TSA2, SNG1, HOR2, HBT1, YDL124W, LRP1, RAP1, ATG7, TPP1, MLP1, AFR1, WTM2, BUD25, YLL056C, GET3, ALD3, SNF1, STE50, GAL3
biological regulation	550 out of 1943 genes, 28.3%	1571 out of 6348 genes, 24.7%	0.01240	ERG2, SDC25, CRN1, SOH1, YLR126C, YPT1, TOS8, SPC19, GCN4, MAL33, MSH2, GET1, DFG5, DPL1, HIT1, RTT102, ABF2, CMP2, KEL2, GCD10, MDM31, TPK2, MKT1, ENB1, RPL13B, DMA1, STD1, ARN1, IRS4, YPT32, MET31, SDS3, SNF3, NTO1, TEL2, CDC42, GLO4, NHP10, SKI2, SSC1, UTR1, CDC55, YPL144W, FAB1, SSL1, CYR1, ATG11, STB5, DAP1, NET1, ATC1, CSG2, ERD1, SUT1, BDF1, SSN2, UTP5, GAL80, GCD7, PCL5, CMK1, HMS1, ARG81, SWD3, SGS1, GID7, DOA4, CRT10, CKS1, NDI1, RNA1, DAL80, VAM7, SPA2, TIF4632, YOL019W, MTQ1, CLB4, SAR1, CCL1, HDA2, CAD1, YCR062W, MSB3, HOG1, SNF11, VHR1, SNF7, YRF1-3, RGT1, SPT20, SWI6, RAS2, HSP104, NRM1, LST8, ARC15, TFB3, REF2, SIR1, NDD1, PTC6, URE2, IRE1, ZAP1, IZH1, CTI6, SPT7, UPC2, FOB1, SNF4, TEP1, STE12, HAT2, COT1, FRE3, RGT2, MMS2, DAD4, NAB2, RLM1, SRB2, MEP2, DBF4, STE7, YRF1-2, TEL1, RSC30, CDC36, CDC7, PET122, CLB6, WSC2, MAK31, SSK1, RFM1, PIH1, SCO1, HUG1, HTB1, GPG1, RHO3, IOC4, MAK3, RSC9, RSC58, DAD2, PUB1, SUT2, PHO85, ISA1, RHO5, YGL039W, RTT106, SWI4, EMP70, SGF29, SSL2, VTA1, MOT3, MFA2, HAT1, ECM32, BEM4, YRM1,

				SRB7, CDC1, PER1, HEK2, MFT1, HCR1, MF(ALPHA)2, MSS116, SHR5, RPS9B, ADE12, HMRA1, YNR063W, YRF1-7, RPS27B, YPD1, PHB2, MND2, GCD6, CBC2, TAF14, UTP15, STO1, DFM1, MRPL44, VMA22, GIP4, DCC1, FUS2, MSS51, HTZ1, ORC2, HSL7, GCS1, HAL9, STE4, APN1, GRX3, RRI2, IMG2, GRX5, RRN6, FRE8, YKU80, TOP3, INO80, EAF5, YBR284W, SPT21, SAC3, RGD1, SPT3, HMX1, GCN1, HHT1, TAF13, PMC1, VPS15, ERV15, TAF2, CLN2, EAF7, SKI3, GCR1, FYV6, PRB1, BST1, GIS1, VPS25, UBP14, GIP3, VMA10, YAP3, KCC4, ESP1, SOD1, PDX3, SPT4, LPD1, SLM2, BRF1, RRI1, HKR1, PBP1, OSH3, CIN5, BUR6, YDR520C, VID24, ARD1, LCB4, PEA2, MET7, TAF4, MAF1, OPY2, BOI2, SUA7, NPR1, MAL23, STF1, YLR278C, KAE1, LTE1, SLF1, TSA2, GIS4, MSS1, GIS2, PRS3, YIH1, NUT2, ERJ5, VCX1, AFR1, WTM2, BUD8, SPO12, DMA2, AFT1, GET3, SUI1, SNF1, PBI2, IBD2, STE50, DOC1, PCL6, SIS2, GCN20, GAL4, PDE2, TAP42, TRA1, PCL8, YPT52, GRX4, NRG2, RPB9, YJL103C, MFA1, DSE2, PSK2, UGA3, RHO4, RNR1, TOS4, PEX22, ESC8, HST2, THP2, PTH2, YLR345W, MOB1, HOS1, STE18, TAT2, PDR8, ORC5, UBC13, PRR1, YOR338W, ADO1, YSP3, HAC1, KAP122, YRF1-6, NPT1, MUK1, TAF10, PRX1, RAD57, CDC14, NPL6, SDA1, LGE1, FPR4, GCD1, HUR1, GYP6, MED11, RPB7, IML1, YER184C, FZF1, PDI1, YRR1, PHD1, SPT10, SPT8, HEM1, GPD1, ELP3, EST2, VAM6, EPS1, PKH1, DFG10, PET111, MBF1, MTF1, RSR1, UBC8, DLS1, MSI1, SIW14, LST7, YLL054C, RPI1, CDC6, PCL1, RAD59, DAL82, GSC2, MKK1, RHO2, DAL81, MAM3, TIF5, YTA7, SRB8, MIH1, RAD50, MED2, NUT1, YOR1, MEC1, SPH1, YOR008C-A, IKI1, CSI1, SLT2, YRF1-1, YRB2, SWI1, BFR1, MAK10, CUP9, MIG3, RME1, GAL83, HPR1, CAF130, AZF1, PBS2, MET18, SIT1, MF(ALPHA)1, SCP160, ASH1, ACA1, CDC26, ESS1, DCN1, SWI5, UBC1, HAP2, MUC1, SKM1, GAL1, SFK1, SWD1, CLB1, DIA4, PRO1, ZRC1, CAC2, SIP1, ARG5,6, UBC6, GDI1, MPD1, EMP24, NHA1, PDR3, PRK1, HSP82, GTR2, YPL230W, CLB2, PCL7, ELP2, VPS74, SLX8, XBP1, SUR4, SLH1, FAR1, GAT1, CHD1, SLM6, SEF1, TAF6, FKS1, NCS2, ARG2, CSR2, RAD3, SGF11, BOI1, YBR014C, YFR055W, TFB1, EMI1, RGD2, SWR1, CAT8, ARF3, HAP1, TOM1, NRG1, BUD2, CLN1, TRS120, YBR033W, YVC1, MRK1, KOG1, CDC43, PGM2, YGL220W, GBP2, HHO1, IXR1, ISU2, SCH9, RPA49, HST4, TFG1, PFK27, STV1, RDS1, LEM3, CLB5, YCS4, GAT2, FPR3, BUD21, TRS130, RPN4, RPS16B, MET28,

				TRR2, ATG17, PCA1, SWA2, PSK1, ARH1, CCC2, RPS30B, YHC3, IRA2, CDC39, MUB1, YOX1, NMD2, FRE4, SLI15, VMA6, RPA14, RFA2, GEA2, RGR1, HCH1, UBC9, TOR1, PPZ1, PTP2, DOT6, CAP2, CTK2, YGR111W, APC1, HBT1, LRP1, INO4, RAP1, YFH1, MLP1, ARN2, ARG80, ASF2, FLO8, ORC4, GAL3
protein amino acid glycosylation	40 out of 1943 genes, 2.1%	73 out of 6348 genes, 1.1%	0.01955	KTR3, VRG4, ALG6, MNN1, PMT5, ALG2, WBP1, PMT1, KRE5, MNT3, SWP1, MNS1, KTR7, ERD1, DPM1, SEC59, KTR2, RER2, MNN9, ALG7, STT3, MNN4, PMT6, ALG12, MNT2, ALG14, KTR1, MNN2, ALG3, PMT2, OST3, GDA1, CWH41, ALG1, VPS74, ALG9, KTR4, PMT3, KTR6, ALG5
biopolymer glycosylation	40 out of 1943 genes, 2.1%	73 out of 6348 genes, 1.1%	0.01955	KTR3, VRG4, ALG6, MNN1, PMT5, ALG2, WBP1, PMT1, KRE5, MNT3, SWP1, MNS1, KTR7, ERD1, DPM1, SEC59, KTR2, RER2, MNN9, ALG7, STT3, MNN4, PMT6, ALG12, MNT2, ALG14, KTR1, MNN2, ALG3, PMT2, OST3, GDA1, CWH41, ALG1, VPS74, ALG9, KTR4, PMT3, KTR6, ALG5
