# Supplementary material for: Performing statistical analyses on quantitative data in Taverna workflows: An example using R and maxdBrowse to identify differentially-expressed genes from microarray data
Source: BMC Bioinformatics. 2008 Aug 7;9:334. doi: 10.1186/1471-2105-9-334 (PMC2528018; doi:10.1186/1471-2105-9-334)
Supplement: Additional file 3 — Nitrogen t-test. [file 1471-2105-9-334-S3.zip › 0.05ttest/0.05Go/cellcomp.pdf]

Result Table

Terms from the Component Ontology with p-value as good or better than 0.05

Gene Ontology term	Cluster frequency	Genome frequency of use	Corrected P-value	Genes annotated to the term
unannotated	24 out of 1943 genes, 1.2%	out of 6348 genes, 0.0%	0	YDL228C, ARS605, YFL015C, YKL033W-A_ALT, Q0270, YKR106W_1, YFL031W_EX2_ALT, PSY1, 2MIC_REP2, Q0155, TLC1_0, OPI6, YNL203C, Q0320, YRF, BUD30, MBB1, CEN13, YLR426W_EX2, Q0167, SNR17A_EX2, OPI8, TY1B_A_LR4, YEL074W
organelle membrane	371 out of 1943 genes, 19.1%	979 out of 6348 genes, 15.4%	3.13e-05	DAP2, ERG2, MTM1, CRC1, YCF1, YPT1, DID2, BNA4, GET1, PMT1, QRI5, ERV29, ERP1, YKE4, MNS1, USO1, VAM3, MDM31, ICY1, YDL119C, ALG7, TPC1, TLG2, ENB1, MNT2, ATF2, ARN1, FLC1, YPT32, YSC83, PEX22, QCR8, COX15, PTH2, COQ1, NUP145, SEC14, IMP1, HUT1, SEN15, PSD2, NEO1, SEC11, SSC1, NDC1, FAB1, COX9, SEC16, KAP122, ERP3, AFG1, COR1, CSG2, ATP8, AVT1, TOM20, SDH4, PEX11, YPL236C, ATP3, TAZ1, DLD1, YBR238C, AVT7, SEC61, YPR071W, ALG14, ATP2, KAP120, SEC24, GPI16, SWF1, MGR1, IML1, PKR1, COX20, NDI1, OST3, CWH41, JEM1, YMC2, VAM7, BCS1, COY1, LSP1, MDL1, GUT2, KAP123, YOL019W, SHY1, EPS1, VAM6, PET309, COP1, PET111, SAR1, CDC50, PEX10, ERG3, NMD5, ALG6, WBP1, NSG2, YIF1,

				ERP2, SLS1, NUP192, CLC1, AQY2, SNF7, YIA6, LST7, VPS20, GPI18, NNF2, YCR023C, RET2, DPM1, HEM13, RER2, SEC31, ERG6, LST8, CBR1, ARC15, ERV2, ZRG17, TIM9, TOM70, EHT1, YOR285W, GPI11, IRE1, CSM4, MAM3, IZH1, YPR114W, MGM1, TPO2, YPR091C, YPR003C, AVT2, ALG9, IMP2, PMT3, SFB3, LST4, ODC2, COT1, ERV41, HMG2, YJL171C, SEC66, VRG4, PBN1, SUR2, MNN1, PMT5, GOT1, SFB2, VAC8, CCE1, YRB2, HFD1, TSC3, RTN1, GNP1, PFA3, ILM1, PEP7, PET122, POM152, UBP16, ENT5, SIT1, SEC27, MBA1, FPR2, KTR1, SCP160, CTR3, DER1, ECM38, DBP5, LIP1, ERG25, PIC2, YPS1, CWH43, DIC1, SCO1, YML018C, GPI19, ATG8, VTH1, GPI14, AAC3, ULP1, NUP100, IVY1, NUP170, ALG2, MNT3, YCL045C, TIM21, SWP1, SEC39, PIB1, EMP70, YDR307W, ZRC1, KTR2, VTA1, YLH47, UBC6, YPC1, STT3, MTR2, VPS38, COG8, EMP24, ALG3, PDH1, SYS1, ODC1, GOS1, ERG9, STF2, YDR128W, PER1, KAP95, GTR2, SHR5, USE1, MSS2, MVB12, PXA2, TPO3, ALG5, COX2, YOR271C, YPR011C, LPP1, PHB2, PEX12, RIM2, PEX13, LAG1, MSP1, BIG1, SEC28, LAS21, APS3, VTC4, VMA21, ERG7, KRE6, DFM1, PIL1, VPS24, VMA22, ALG12, TOM71, MSS51, MTG2, USA1,

				TMS1, SED4, PMT2, AVT4, ERF2, TCM62, CYC7, YMR118C, ALG1, YVC1, SOM1, KOG1, TOM6, SEN54, SAC3, YMR221C, MCD4, CDS1, HMX1, QCR9, DGA1, SCH9, PHO8, OAC1, RET3, PMC1, VPS15, ATG18, STV1, SXM1, SEN2, ERG28, PHS1, COX1, BST1, APL3, NUP84, SEC13, MNN9, VPS25, RCE1, MRL1, SEC12, YLR050C, VMA10, COQ3, SWA2, TVP15, ARH1, GDA1, CCC2, SEC23, SSA2, YHC3, VBA1, VPS52, OMS1, TOM40, MIA40, YIP1, OMA1, VID24, YMR171C, VPS73, MCH1, NCA2, VMA6, TSC13, FET5, LCB4, COX12, THP1, HSE1, MDJ2, NUP157, OPY2, TOR1, COX7, STF1, YLL053C, TSC10, SEC59, NUP85, NUS1, CSH1, POM34, PMT6, SRN2, CTP1, YEA6, MSS1, SEC21, YJL045W, OM45, CDC31, MLP1, ARN2, VCX1, GPI12, PCS60, COX18, MTG1, CNE1, NCP1, SNF1
plasma membrane	154 out of 1943 genes, 7.9%	354 out of 6348 genes, 5.6%	4.12e-05	MUC1, TIR1, PDR5, SKM1, CRH1, DAL3, HOL1, ITR1, SSU1, SFK1, DFG5, RHO5, FRE2, SSO2, YPS6, DNF1, BUL1, YPT52, HXT5, MFA1, MTL1, MRH1, DAN1, MFA2, STD1, HXT11, ARN1, NHA1, KIN2, RHO4, FAT1, OPT1, HRR25, EXG2, YPS3, SNF3, AGP1, CWP1, ITR2, CDC42, SEC15, DDI1, FPS1, STE18, TAT2, TPO3, DNF2, ATO3, LAS21, FKS1, CYR1, PMP2, TRE1, ERG7, YHL044W, ALP1, YNL194C, AVT7, HXT16,

				YOR071C, HXT9, FLO5, SGE1, BSP1, MUP1, STE4, MID2, CAN1, HOR7, KOG1, DAL5, ENA5, YOL019W, PHO84, RAX1, TAT1, ENA1, RSR1, AQY2, MID1, CLC1, AZR1, YLL054C, ADY2, LEM3, CDC6, RSB1, RAS2, SEC18, APL3, GSC2, HSP12, LST8, RHO2, GAP1, BAP3, PTR2, SUL1, PCA1, QDR2, SMF1, SLM2, VHT1, TPO2, HKR1, PHO89, SNF4, JEN1, GAS4, HXT15, YOR1, FLO1, FRE4, FRE3, SEC9, FET5, LCB4, GAS2, YOR378W, MST28, BAP2, RGT2, KRE1, OPY2, YKT6, MCH5, TOR1, MEP2, GNP1, YLL053C, PPZ1, FUI1, HXT13, FUR4, SKG3, MST27, SED1, GIS4, HXT2, CTR3, FEN2, FCY2, ARN2, ERG25, AXL2, GAL2, BUD8, CWH43, MAL11, YCK2, MKC7, RHO3, ENA2, SAM3
cell	1814 out of 1943 genes, 93.4%	5753 out of 6348 genes, 90.6%	9.47e-05	SNX3, SDC25, SIM1, SOH1, TR(UCU)E, TUF1, SPC19, GCN4, DID2, URM1, BCH1, LOS1, IPP1, MUM2, YIR035C, SFI1, DFG5, DYS1, AGX1, HIT1, QRI5, BSC2, YAR029W, DNF1, STP3, RTT102, ABF2, CMP2, AGA1, GCD10, MTO1, TAH1, MDM31, YDL119C, PUS4, CLU1, MNT2, RPL13B, CBP6, DMA1, ATF2, STD1, PET130, IRS4, YPT32, YSC83, TYW1, NTO1, TOS3, TK(CUU)J, MET14, TG(UCC)N, LYS1, TS(AGA)D2, CWP1, CYM1, ITR2, CDC42, SEC14, YOR286W, CCP1, HUT1, NAT1, SEN15, CDA1, YGR207C, YLR419W,

				ATO3, SSC1, HSP78, FAB1, TRM3, CYR1, ATG11, BUD13, DAP1, NET1, CSG2, LSG1, ERD1, YPL236C, YTA6, BDF1, URB1, GAL80, YPR071W, GCD7, CMK1, TE(UUC)J, ATP2, RIB4, GRC3, SGS1, GID7, SHE1, RKI1, DOA4, SOL3, GPM3, CKS1, RNA1, CAN1, SUV3, YDR161W, HOM3, MSW1, LSP1, HOR7, TIF4632, YOL019W, ENA5, PHO84, PET309, TQ(UUG)D3, RRD1, CWC27, SAR1, CCL1, HDA2, CAD1, RET1, YNL045W, ERP2, ENA1, VHR1, GLO1, IMD2, SNF7, YIA6, MRP2, SNR51, GPI18, SPT20, SUP35, SWI6, RAS2, HSP104, PCK1, AI5_BETA, HEM13, RIM20, RER2, TR(ACG)K, TG(GCC)B, PYK2, MDH2, SEC31, ERG6, FAA4, LST8, SGT2, CBR1, ARC15, TFB3, POP8, REF2, ERV2, NDD1, TIM9, TOM70, CET1, PTC6, URE2, EHT1, GPI11, TI(AAU)L1, DSN1, ARG8, ZAP1, YPR114W, TH(GUG)M, YHB1, FOB1, IDP1, SNF4, INO1, STE12, MRPL3, CSL4, HAT2, ODC2, COT1, RPC37, FLO1, SEC66, YBR204C, FRE3, RAD16, GLC8, LHS1, UTR2, PMT5, PET10, RMI1, GOT1, TRP5, ILV5, LAG2, MRP20, BAP2, YTH1, NAB2, RLM1, SRB2, KTR7, DIN7, UBX6, KAP104, RLI1, MAM1, CDC7, MSN5, PRP5, MST27, POM152, UBP16, POT1, SNR11, KTR1, FPR2, MAK31, DUR1,2, CTR3, FEN2, FMC1, SSK1, ERG25, ATP11, RFM1,

				PIH1, HEM3, DIC1, YML018C, GPI19, ATG8, HUG1, ATG26, HTB1, PRS4, SET5, RHO3, YER130C, RSC9, IVY1, PUB1, SUT2, UBR1, YOR387C, DAL3, ARO4, YAL061W, ISA1, RHO5, YGL039W, RTT106, SWI4, VTH2, SMC6, TD(GUC)J1, SEC39, TG(CCC)D, NCA3, BUL1, EMP70, MYO1, SGF29, BIO3, MOT3, YLH47, MNN4, RNH203, PSY4, MTR2, MFA2, IPT1, VPS38, ECM32, EXG2, GOS1, SMB1, STF2, ERG9, YMR291W, AGP1, HCR1, MFT1, HEK2, SHR5, RPS9B, ADE12, TS(AGA)B, YPR011C, LPP1, YPD1, PEX12, RPL9A, LAS21, PAN2, TA(UGC)L, SLY41, TR(UCU)B, LEU4, ERG7, SNT309, KRE6, YTM1, TQ(UUG)D1, SPI1, MRPL44, RNR2, HXT16, ECM21, DCC1, FUS2, NOP16, FLO5, ACF2, HTZ1, ILV2, SER3, SSP120, ADH7, HSL7, TIR4, TS(AGA)A, PMT2, STE4, ERF2, RRI2, GRX3, PGI1, SRM1, IMG2, RRN6, OSW2, FRE8, PRO2, YKU80, HPR5, EAF5, YBR284W, PDB1, SAC3, RAX1, HSP31, TAT1, CDS1, CTL1, YBP2, SAD1, YGR043C, GCN1, TAF13, ARO1, FCY22, ERV15, TS(AGA)L, CLN2, EAF7, IDH1, SKI3, SEN1, SXM1, YHR020W, GCR1, ADY2, SNR56, SAP155, COX17, ERG28, MNP1, KEL3, TGL3, PRB1, SEC18, YJR149W, BST1, TE(CUC)D, NUP84, MNN9, ADE5,7, CTM1, DSS1,

				SER1, GIP3, SSE2, YOL054W, GAD1, NAS6, ESP1, PRP42, NIP1, YHC1, DTR1, QDR2, GDA1, SLM2, BRF1, SSA2, RRI1, HKR1, SHM1, PHO89, BUD20, MAM33, BUR6, TOM40, MIA40, VID24, MCH1, YAH1, SNR6, ARD1, TSC13, LCB4, MIF2, FMP46, HSE1, KRE1, MDJ2, LRO1, TAF4, KRE5, NOP53, TT(AGU)J, MAL23, CCT2, STF1, PDA1, YLL053C, BPH1, TM(CAU)J1, ECM11, RPS0A, NUP85, YLR278C, KAE1, POM34, TSA2, GIS4, SEC21, CYK3, PRP6, RPL31B, SLX9, NUT2, ERJ5, TL(UAA)J, BUD8, ARG1, SPO12, COX18, DMA2, URA1, CNE1, AFT1, GET3, NCP1, SNF1, JLP2, STE50, SAM3, PAI3, DOC1, TDP1, SIS2, SSU72, PDR5, MRP10, ITR1, TRM10, PDE2, PMT1, YPS6, ERV29, TRA1, ILS1, LEU2, ERP1, YPR004C, SLD5, ARP2, PCI8, USO1, MRPL33, BUD7, NRG2, RPB9, YPR127W, RPC82, TPC1, UTP13, UGA3, PSK2, MNN2, OPT1, TOS4, ESC8, PEX22, GAR1, HST2, YNL320W, COX15, MST1, CHS3, NOP14, KRE33, HOS1, TQ(UUG)D2, YPR117W, STE18, PDR8, TAT2, ORC5, UBC13, PDS1, YGL157W, NEO1, SEC11, OSH6, NDC1, MSS18, ADO1, MHT1, COX9, CTF13, HAC1, RNR4, KAP122, HEF3, YRF1-6, AFG1, KEX2, COR1, MSH5, NSG1, YDL203C, TS(AGA)E, TOM20, SDH4, MUK1,

				FBP26, ATP3, PRX1, SEY1, DLD1, AVT7, ZIP1, CDC14, LOC1, URA8, ENT1, DBP2, SDA1, HXT9, KAP120, YFL067W, YPL229W, HUR1, MED11, ERG12, MGR1, ILV3, YER184C, CWH41, PDI1, PHD1, YMC2, SPT8, CUP1-2, URA2, BCS1, COY1, POP6, GPD1, HEM1, GUT2, TYS1, LEU9, EST2, VAM6, DFG10, COP1, PUS6, MBF1, TIR2, MSF1, WBP1, TE(UUC)L, GRE1, NSG2, YBR242W, GIP2, CAR2, YIF1, RRP1, RSR1, CLC1, AQY2, YCL074W, SIW14, VPS20, CDC5, PCL1, RSB1, NNF2, YCR023C, NAB6, SNR61, MKK1, AIR1, DRS2, ACO1, PPG1, ARO3, DRS1, ZRG17, PTR2, SNA2, DAL81, KRI1, GPH1, GLT1, CUS2, FUN12, MAM3, TIF5, RPS30A, MCM16, SRB8, RNR3, MIH1, RAD50, SCT1, YPR091C, FMS1, YPL141C, PMT3, NUT1, IML3, YOR1, YHL017W, ERV41, HMG2, MEC1, SPH1, PBN1, IKI1, SUR2, GUS1, SLT2, CSI1, DCS2, SSD1, CDC10, YRF1-1, RPN1, YRB2, SUP45, HFD1, TR(UCU)J1, HSM3, CUP9, GAL83, DBP8, TC(GCA)P1, ICL2, NMA1, HXT12, CAF130, HPR1, PBS2, PPT1, MET18, MBA1, SCP160, ECM38, RAD26, KAR1, FCY2, YPS1, YDR338C, CWH43, HSP60, SSA4, DIB1, YGL080W, VTH1, BRE4, SWI5, IES6, AAC3, GTT3, TM(CAU)J3, ENA2, PHR1, REV7, MSE1, ALG2, RPL13A, GAL1, YGR130C, SFK1, CLB1, DIA4, RDH54,

				TIM21, YCL045C, PIB1, HEM12, ZRC1, CAC2, AAH1, SIP1, PES4, ARG5,6, HXT5, UBC6, GDI1, RPS24B, STT3, APA1, SFA1, DOA1, YMR031C, NDE1, PTC7, TQ(UUG)B, ARO7, LOT5, SDL1, TE(UUC)C, HXT11, EMP24, NHA1, PDR3, FAT1, PDH1, SYS1, YMR226C, HRR25, HSP82, CHL1, YPL230W, CLB2, SMX2, ATG19, ELP2, CPR5, SUR4, PRE5, SLH1, AAR2, TPO3, GAT1, CHD1, YPR172W, RPL16B, SEF1, CSF1, RIM2, IDS2, SAP4, BIG1, SEC28, POP3, YOL048C, FKS1, ARG2, APS3, MRPS18, CSR2, RPL35A, ALP1, RAD3, GDH1, PNG1, BOI1, VPS24, ALG12, RGD2, MEF1, TOM1, OAZ1, USA1, SGE1, SAK1, RPO21, SED4, PCM1, CLN1, GIM3, YMR118C, PAN6, YBR033W, PUS1, CDC43, PHO12, HUB1, YMR221C, YGL220W, PGM2, GBP2, MCD4, PRP18, FAA2, YPR174C, ADD37, ISU2, IXR1, HYS2, YDR061W, PHO8, RPA49, RPL8A, YNR066C, CAK1, KRS1, MID1, TFG1, ATG18, RPS0B, STV1, GLO2, SEN2, YKL071W, LEM3, SEC17, GAT2, FPR3, PET112, BUD21, COX1, THI4, AMD1, SEC13, HSP12, MBR1, TMA17, STP4, YLR050C, COS9, TRR2, MET28, COQ3, ATG17, APQ12, ATG1, PCA1, SWA2, PSK1, TVP15, SIP18, CCC2, SNR19, RPL6B, ATG3, AIP1, YHC3, VBA1, BNR1, CDC39, YBL054W, HXT15,

				TR(UCU)K, YOX1, YIP1, RPC40, ORM1, SVS1, VMA6, VAS1, HPT1, SSP1, ISY1, YOR378W, GEA2, MSM1, TH(GUG)E1, YKT6, YNL134C, TAD3, ADE8, LYS2, COX23, SMX3, LYS12, PPZ1, TN(GUU)K, PTP2, DOT6, YSC84, CTK2, YGR111W, APC1, SKG3, UBX3, HOR2, YEA6, YJL045W, INO4, LSM3, OM45, YDR089W, YLR004C, CDC31, ATG7, TG(GCC)M, INM1, CCT4, MLP1, TR(CCG)L, GLY1, GPI12, TP(AGG)C, STU1, TPA1, KTR4, SUC2, MAL11, ORC4, GAL3, WRS1, HOF1, ERG2, YEL007W, ATG2, CRN1, CRC1, YCF1, YOR223W, YLR126C, ERG11, IST3, YPT1, RPG1, RPT3, TOS8, RFS1, MAL33, MSH2, BNA4, CRH1, GET1, AST1, RPL5, DPL1, YNL217W, MDY2, YKE4, KEL2, SVF1, ECM31, MSD1, TPK2, MKT1, MTL1, MPA43, ENB1, TLG2, TS(AGA)J, DED1, ARN1, FLC1, FCF2, YFR006W, MET31, TG(GCC)C, MRPL25, SDS3, SNF3, QCR8, SME1, TEL2, COQ1, SMM1, PUS7, IMP1, GLO4, POL5, NHP10, TR(UCU)J2, SAM4, VIK1, ERG5, SKI2, PAC10, UTR1, CDC55, YPL144W, SEC16, DOG2, EFR3, SSL1, ERP3, STB5, TG(UCC)O, TT(UGU)P, YNR024W, UGA2, TPS3, NNF1, SRL2, ATC1, ADE6, VIP1, TRM12, KAR2, ATP8, AVT1, SUT1, PEX11, APD1, YBR238C, SSN2, YIP5, SEC61, UTP5, PCL5, HMS1, RPL9B, ALG14,

				ARG81, SEC24, UTP21, PRS2, SWD3, AME1, MUP1, NDI1, YSF3, DAL80, JEM1, BET4, BGL2, VAM7, SPA2, RPS24A, DAL5, MDL1, KAP123, MTQ1, CLB4, PEX10, GSH2, ERG3, YCR062W, NMD5, ALG6, RDS3, MSC1, MSB3, HOG1, SNF11, RPL7B, SLS1, RPA135, TOS1, LSM4, CFT1, YRF1-3, RGT1, RPL22B, CCA1, CWC23, RET2, EDC2, NRM1, ADH4, SIR1, YOR285W, IRE1, IZH1, FIT1, CTI6, PIN2, SPT7, UPC2, KIP1, AVT2, HNT1, LST4, RPT4, VRG4, LSM5, MNN1, MRPL20, LEU1, TKL1, MRPL40, MST28, RGT2, MMS2, DAD4, CCE1, TSC3, TQ(UUG)E1, MEP2, AXL1, DBF4, FUI1, HXT13, STE7, ZTA1, PPA2, ILM1, YRF1-2, PEP7, TEL1, RSC30, PRM2, SER33, CDC36, PET122, ENT5, HXT2, CLB6, SEC27, WSC2, DER1, DBP5, PAC2, TUB3, NAS2, YLR046C, TR(UCU)M2, YLR247C, PIC2, NOC4, RRP14, PGS1, SCO1, BRR2, EFT2, CHS6, TMA20, PRD1, ULP1, TG(GCC)P2, TR(ACG)D, GPG1, IOC4, MAK3, FIP1, MRPL37, RPL35B, RSC58, DAD2, NOP1, KTR3, NUP170, MCK1, YRO2, NFT1, YGR250C, PHO85, SNX41, SSU1, NTG2, MNT3, YKR070W, TKL2, SWP1, SCEI, CCT6, PRP28, TE(UUC)P, ACO2, YDR307W, YKR096W, RRP5, SSL2, VTA1, TS(AGA)M, MRPL27, YPC1, DAN1, RPO31,

				HAT1, BEM4, ODC1, YRM1, UBA1, YBR063C, SRB7, YDR128W, PRI2, CDC1, PER1, KAP95, RAD1, MSS116, TV(CAC)D, SEC15, IDH2, HMRA1, PRP8, URA7, YNR063W, FPS1, MVB12, YRF1-7, YOR271C, RPS27B, TA(AGC)K2, NOP13, GTO1, SNM1, PHB2, MND2, TA(UGC)A, GCD6, PEX13, LAG1, YKR104W, CBC2, TAF14, TAL1, RNT1, DLD2, IMD4, CRP1, YHL044W, UBP3, ATG5, UTP15, IDI1, STO1, MOD5, DFM1, VMA22, SIP5, GIP4, HPA3, MSS51, SHC1, MTG2, ORC2, GCS1, HAL9, KNH1, TMA10, BSP1, SUE1, ARO2, CDC21, APN1, CYC7, ROT2, GRX5, YBR025C, RRB1, YIL171W, TOP3, INO80, SEN54, SPT21, RGD1, SPT3, RML2, QCR9, HMX1, DGA1, PMU1, SCW4, HHT1, SKI6, PMC1, YNL144C, VPS15, AZR1, ECM7, TAF2, FYV6, YLR412W, PHS1, ALD2, HIS5, APL3, MDM20, GIS1, VPS25, UBP14, HRT3, RCE1, GAP1, SEC12, VMA10, CPR3, YAP3, KCC4, FDH1, PRM7, SOD1, SPT4, PFS2, LPD1, HSP26, YFR011C, URB2, SEC23, DAL4, MRPL39, VPS52, PBP1, OMS1, OSH3, MXR1, GAS4, CIN5, TS(AGA)D3, OMA1, YDR520C, SWH1, SEC9, ADE3, VPS73, YNK1, FET5, YNL274C, PTH1, THP1, PEA2, MET7, YNL168C, NUP157, MAF1, YNL024C, SUI3, OPY2, BOI2, SUA7, MCH5, NBP1, DUS3, NPR1, ISM1, SBE22,

				RPT6, YKR075C, SEC59, LTE1, CSH1, SLF1, PMT6, FSH2, YLR108C, CTP1, MSS1, TMT1, YOR059C, GIS2, PRS3, ICL1, YIH1, SNU114, VCX1, AFR1, WTM2, GCV2, LSB3, ACN9, RSM10, MTG1, ERB1, ALD3, SUI1, IBD2, PBI2, DAP2, PCL6, ADH3, MRPL8, MTM1, HAS1, TIR1, YCR087C-A, PSE1, GAL4, GCN20, AI4, GRE3, CAR1, RPA190, PCL8, AUS1, RPS29A, PIM1, MIS1, MNS1, GIM4, YPT52, GRX4, VAM3, YJL103C, MFA1, ICY1, DBF20, LDB17, ALG7, MRH1, DSE2, AGA2, TYR1, KIN2, RHO4, TV(UAC)B, RNR1, PFK2, YPS3, HHT2, THP2, YPR022C, PTH2, MOB1, RBG2, YLR345W, NUP145, ARX1, DDI1, MDM35, PDS5, PSD2, DCS1, PRR1, RUB1, FMT1, RHR2, YOR262W, TRE1, FLO10, NPT1, MAG1, TAF10, TAZ1, YNL194C, PUF2, ADY3, TQ(UUG)C, HYP2, RAD57, YOR071C, TN(GUU)C, SEC53, CDC4, SGN1, NPL6, LGE1, FPR4, GCD1, ADD66, HSP10, GYP6, GPI16, SWF1, ECM18, NTH1, IML1, RPB7, YNL247W, UTP22, YPR157W, PKR1, FZF1, COX20, OST3, MID2, YRR1, SPT10, SOL4, ELP3, AAT2, PAN5, TG(GCC)J2, SHY1, TG(GCC)O2, EPS1, PKH1, PET111, CIN2, CDC50, MTF1, ATH1, YJL213W, TE(UUC)E1, PEP12, HAM1, UBC8, CCT5, SSF2, NUP192, ASN2, YPR118W, DLS1, MSI1, LST7, YLL054C,

				RPI1, CDC6, DPM1, NIT3, AST2, SIZ1, SLC1, RAD59, ALD4, DAL82, TE(UUC)M, SNR58, GSC2, LSC2, TRF5, AAP1, RHO2, NOG1, TR(UCU)D, NAM2, RPF1, SUL1, MDM30, CSM4, RAD34, SMD3, YTA7, SMF1, YAL049C, MGM1, RPL24B, TPO2, BRR1, DAL7, TS(AGA)D1, YPR003C, ALG9, YOR390W, FLO9, IMP2, MED2, YOR283W, SFB3, GUK1, PGA3, PPH3, FAS2, YJL171C, YOR008C-A, TUB1, TE(UUC)B, MET10, DPS1, TS(UGA)P, SFB2, APT1, HIS2, VAC8, UMP1, SWI1, RTN1, MAK10, BFR1, ILV1, GNP1, DEG1, PFA3, MIG3, RME1, MET2, YEL043W, SLM5, AZF1, ARP10, SIT1, ABP140, ASH1, YDJ1, RIB5, ACA1, CDC26, HOM2, DSE4, LIP1, CNS1, AXL2, GAL2, RFC3, ECM27, EFT1, TG(CCC)O, ESS1, HXK1, UBC1, HAP2, GPI14, YCK2, PRT1, ZWF1, MKC7, YBR220C, YNL176C, NUP100, MUC1, AI2, TR(UCU)M1, POS5, SKM1, ECI1, YLR281C, HOL1, EHD3, HSP42, ESF1, SWD1, PRO1, SSO2, FRE2, STB2, NAT2, CTF18, RRF1, KTR2, CSE4, MPD1, MRPL11, MIC17, TQ(UUG)E2, COG8, RTS2, ALG3, PRK1, TA(UGC)O, MRP51, AHA1, IES4, ASP1, CDC2, YPL191C, REC107, GTR2, DPB2, HIS4, PCL7, TIR3, VPS74, YHR113W, NRD1, SLX8, USE1, MSS2, XBP1, PFK1, PXA2, YNR029C, TH(GUG)K, CUP1-1, ALG5, DNF2, FAR1, COX2, SLM6, TAF6,

				MSP1, YBR139W, AEP2, SKG6, NCS2, VTC4, ACB1, PMP2, TIF6, YIL064W, HOM6, VMA21, GND1, SPC97, STR3, YDR341C, EMG1, SGF11, THR4, YBR014C, RIB3, ASK10, PIL1, RPL27A, TFB1, YFR055W, TOM71, RPA43, IRR1, TL(GAG)G, CAT8, SWR1, ARF3, HAP1, TMS1, NRG1, NIF3, BUD2, ENT4, TRS120, AVT4, TCM62, DUT1, ALG1, YVC1, SOM1, NOC2, KOG1, STB3, TOM6, AAT1, NDE2, TS(UGA)E, HMF1, RPL8B, RPL18A, HHO1, SCH9, RET3, OAC1, SGA1, VHS1, HST4, OYE2, PFK27, RDS1, ECM29, CLB5, PRM5, YOL163W, YCS4, YMR31, SDS24, RPN4, TRS130, MRL1, RPS16B, BAP3, RAD28, ARH1, TQ(UUG)L, VHT1, MRPL50, RPS30B, ABD1, YML081W, MSH4, IRA2, JEN1, MUB1, NMD2, SLI15, FRE4, YMR171C, NCA2, IST1, GAS2, RFA2, RPA14, SNO1, COX12, RGR1, HCH1, UBC9, URA6, SUA5, DTD1, SRL3, YIL166C, TOR1, RRP9, COX7, ADH2, LSB1, FUR4, TSC10, SHM2, NUS1, DIM1, CAP2, SED1, SNG1, HBT1, COS10, SET7, YDL124W, YAL065C, LRP1, SRN2, YOR246C, UTP20, GTO3, RAP1, NPY1, TPP1, ARG3, YFH1, BDH1, TE(UUC)K, ARN2, ARG80, MRM1, MRS1, ASF2, YPL109C, PCS60, FLO8, IPI3, THS1, YFL054C, LIP5, SRP72, FRS2, KTR6
endomembrane system	212 out of 1943 genes, 10.9%	522 out of 6348 genes, 8.2%	0.00011	ERG2, NUP100, NUP170, HAS1, YPT1, ALG2, GET1,

				PMT1, QRI5, MNT3, YCL045C, ERV29, SWP1, ERP1, SEC39, YKE4, MNS1, USO1, YDR307W, KTR2, UBC6, ALG7, YPC1, STT3, MNT2, MTR2, ATF2, EMP24, COG8, FLC1, ALG3, SYS1, YPT32, GOS1, ERG9, PRI2, PER1, KAP95, SHR5, NUP145, SEC14, HUT1, USE1, SEN15, ALG5, PSD2, LPP1, NEO1, SEC11, NDC1, SEC16, LAG1, BIG1, SEC28, LAS21, KAP122, ERP3, APS3, VMA21, ERG7, NSG1, KRE6, CSG2, TOM20, TAZ1, DFM1, YBR238C, SEC61, VMA22, YPR071W, TOM71, ALG12, ALG14, KAP120, SEC24, USA1, GPI16, SWF1, SED4, PKR1, PMT2, OST3, CWH41, ERF2, ALG1, JEM1, COY1, GUT2, KAP123, SEN54, EPS1, SAC3, COP1, SAR1, CDS1, MCD4, ERG3, HMX1, YPR174C, DGA1, NMD5, ALG6, WBP1, NSG2, RET3, YIF1, ERP2, VPS15, NUP192, AQY2, CLC1, STV1, SXM1, LST7, SEN2, GPI18, NNF2, ERG28, DPM1, RET2, PHS1, BST1, APL3, RER2, NUP84, MNN9, SEC13, SEC31, LST8, RCE1, SEC12, YLR050C, ERV2, ZRG17, APQ12, TOM70, SWA2, GPI11, IRE1, CSM4, TVP15, IZH1, YPR114W, GDA1, CCC2, SEC23, YPR091C, SNF4, YPR003C, ALG9, PMT3, SFB3, LST4, YIP1, ERV41, YJL171C, HMG2, SEC66, SWH1, VID24, VRG4, PBN1, SUR2, TSC13, MNN1, LCB4, PMT5, GOT1, THP1, SFB2,

				NUP157, YRB2, TSC3, RTN1, HFD1, BFR1, TOR1, YLL053C, ILM1, SEC59, NUP85, GAL83, NUS1, POM34, PMT6, POM152, ENT5, COS10, SEC27, SEC21, KTR1, FPR2, SCP160, CTR3, DER1, OM45, DBP5, CDC31, MLP1, ERG25, LIP1, YPS1, GPI12, CWH43, GPI19, COX18, CNE1, GPI14, ULP1, NCP1, GTT3, SNF1
cell part	1813 out of 1943 genes, 93.3%	5752 out of 6348 genes, 90.6%	0.00013	SNX3, SDC25, SIM1, SOH1, TR(UCU)E, TUF1, SPC19, GCN4, DID2, URM1, BCH1, LOS1, IPP1, MUM2, YIR035C, SFI1, DFG5, DYS1, AGX1, HIT1, QRI5, BSC2, YAR029W, DNF1, STP3, RTT102, ABF2, CMP2, AGA1, GCD10, MTO1, TAH1, MDM31, YDL119C, PUS4, CLU1, MNT2, RPL13B, CBP6, DMA1, ATF2, STD1, PET130, IRS4, YPT32, YSC83, TYW1, NTO1, TOS3, TK(CUU)J, MET14, TG(UCC)N, LYS1, TS(AGA)D2, CWP1, CYM1, ITR2, CDC42, SEC14, YOR286W, CCP1, HUT1, NAT1, SEN15, CDA1, YGR207C, YLR419W, ATO3, SSC1, HSP78, FAB1, TRM3, CYR1, ATG11, BUD13, DAP1, NET1, CSG2, LSG1, ERD1, YPL236C, YTA6, BDF1, URB1, GAL80, YPR071W, GCD7, CMK1, TE(UUC)J, ATP2, RIB4, GRC3, SGS1, GID7, SHE1, RKI1, DOA4, SOL3, GPM3, CKS1, RNA1, CAN1, SUV3, YDR161W, HOM3, MSW1, LSP1, HOR7, TIF4632, YOL019W, ENA5, PHO84, PET309, TQ(UUG)D3, RRD1,

				CWC27, SAR1, CCL1, HDA2, CAD1, RET1, YNL045W, ERP2, ENA1, VHR1, GLO1, IMD2, SNF7, YIA6, MRP2, SNR51, GPI18, SPT20, SUP35, SWI6, RAS2, HSP104, PCK1, AI5_BETA, HEM13, RIM20, RER2, TR(ACG)K, TG(GCC)B, PYK2, MDH2, SEC31, ERG6, FAA4, LST8, SGT2, CBR1, ARC15, TFB3, POP8, REF2, ERV2, NDD1, TIM9, TOM70, CET1, PTC6, URE2, EHT1, GPI11, TI(AAU)L1, DSN1, ARG8, ZAP1, YPR114W, TH(GUG)M, YHB1, FOB1, IDP1, SNF4, INO1, STE12, MRPL3, CSL4, HAT2, ODC2, COT1, RPC37, FLO1, SEC66, YBR204C, FRE3, RAD16, GLC8, LHS1, UTR2, PMT5, PET10, RMI1, GOT1, TRP5, ILV5, LAG2, MRP20, BAP2, YTH1, NAB2, RLM1, SRB2, KTR7, DIN7, UBX6, KAP104, RLI1, MAM1, CDC7, MSN5, PRP5, MST27, POM152, UBP16, POT1, SNR11, KTR1, FPR2, MAK31, DUR1,2, CTR3, FEN2, FMC1, SSK1, ERG25, ATP11, RFM1, PIH1, HEM3, DIC1, YML018C, GPI19, ATG8, HUG1, ATG26, HTB1, PRS4, SET5, RHO3, YER130C, RSC9, IVY1, PUB1, SUT2, UBR1, YOR387C, DAL3, ARO4, YAL061W, ISA1, RHO5, YGL039W, RTT106, SWI4, VTH2, SMC6, TD(GUC)J1, SEC39, TG(CCC)D, NCA3, BUL1, EMP70, MYO1, SGF29, BIO3, MOT3, YLH47, MNN4, RNH203, PSY4, MTR2, MFA2, IPT1,

				VPS38, ECM32, EXG2, GOS1, SMB1, STF2, ERG9, YMR291W, AGP1, HCR1, MFT1, HEK2, SHR5, RPS9B, ADE12, TS(AGA)B, YPR011C, LPP1, YPD1, PEX12, RPL9A, LAS21, PAN2, TA(UGC)L, SLY41, TR(UCU)B, LEU4, ERG7, SNT309, KRE6, YTM1, TQ(UUG)D1, SPI1, MRPL44, RNR2, HXT16, ECM21, DCC1, FUS2, NOP16, FLO5, ACF2, HTZ1, ILV2, SER3, SSP120, ADH7, HSL7, TIR4, TS(AGA)A, PMT2, STE4, ERF2, RRI2, GRX3, PGI1, SRM1, IMG2, RRN6, OSW2, FRE8, PRO2, YKU80, HPR5, EAF5, YBR284W, PDB1, SAC3, RAX1, HSP31, TAT1, CDS1, CTL1, YBP2, SAD1, YGR043C, GCN1, TAF13, ARO1, FCY22, ERV15, TS(AGA)L, CLN2, EAF7, IDH1, SKI3, SEN1, SXM1, YHR020W, GCR1, ADY2, SNR56, SAP155, COX17, ERG28, MNP1, KEL3, TGL3, PRB1, SEC18, YJR149W, BST1, TE(CUC)D, NUP84, MNN9, ADE5,7, CTM1, DSS1, SER1, GIP3, SSE2, YOL054W, GAD1, NAS6, ESP1, PRP42, NIP1, YHC1, DTR1, QDR2, GDA1, SLM2, BRF1, SSA2, RRI1, HKR1, SHM1, PHO89, BUD20, MAM33, BUR6, TOM40, MIA40, VID24, MCH1, YAH1, SNR6, ARD1, TSC13, LCB4, MIF2, FMP46, HSE1, KRE1, MDJ2, LRO1, TAF4, KRE5, NOP53, TT(AGU)J, MAL23, CCT2, STF1, PDA1, YLL053C, BPH1,

				TM(CAU)J1, ECM11, RPS0A, NUP85, YLR278C, KAE1, POM34, TSA2, GIS4, SEC21, CYK3, PRP6, RPL31B, SLX9, NUT2, ERJ5, TL(UAA)J, BUD8, ARG1, SPO12, COX18, DMA2, URA1, CNE1, AFT1, GET3, NCP1, SNF1, JLP2, STE50, SAM3, PAI3, DOC1, TDP1, SIS2, SSU72, PDR5, MRP10, ITR1, TRM10, PDE2, PMT1, YPS6, ERV29, TRA1, ILS1, LEU2, ERP1, YPR004C, SLD5, ARP2, PCI8, USO1, MRPL33, BUD7, NRG2, RPB9, YPR127W, RPC82, TPC1, UTP13, UGA3, PSK2, MNN2, OPT1, TOS4, ESC8, PEX22, GAR1, HST2, YNL320W, COX15, MST1, CHS3, NOP14, KRE33, HOS1, TQ(UUG)D2, YPR117W, STE18, PDR8, TAT2, ORC5, UBC13, PDS1, YGL157W, NEO1, SEC11, OSH6, NDC1, MSS18, ADO1, MHT1, COX9, CTF13, HAC1, RNR4, KAP122, HEF3, YRF1-6, AFG1, KEX2, COR1, MSH5, NSG1, YDL203C, TS(AGA)E, TOM20, SDH4, MUK1, FBP26, ATP3, PRX1, SEY1, DLD1, AVT7, ZIP1, CDC14, LOC1, URA8, ENT1, DBP2, SDA1, HXT9, KAP120, YFL067W, YPL229W, HUR1, MED11, ERG12, MGR1, ILV3, YER184C, CWH41, PDI1, PHD1, YMC2, SPT8, CUP1-2, URA2, BCS1, COY1, POP6, GPD1, HEM1, GUT2, TYS1, LEU9, EST2, VAM6, DFG10, COP1, PUS6, MBF1, TIR2, MSF1, WBP1, TE(UUC)L, GRE1, NSG2,

				YBR242W, GIP2, CAR2, YIF1, RRP1, RSR1, CLC1, AQY2, YCL074W, SIW14, VPS20, CDC5, PCL1, RSB1, NNF2, YCR023C, NAB6, SNR61, MKK1, AIR1, DRS2, ACO1, PPG1, ARO3, DRS1, ZRG17, PTR2, SNA2, DAL81, KRI1, GPH1, GLT1, CUS2, FUN12, MAM3, TIF5, RPS30A, MCM16, SRB8, RNR3, MIH1, RAD50, SCT1, YPR091C, FMS1, YPL141C, PMT3, NUT1, IML3, YOR1, YHL017W, ERV41, HMG2, MEC1, SPH1, PBN1, IKI1, SUR2, GUS1, SLT2, CSI1, DCS2, SSD1, CDC10, YRF1-1, RPN1, YRB2, SUP45, HFD1, TR(UCU)J1, HSM3, CUP9, GAL83, DBP8, TC(GCA)P1, ICL2, NMA1, HXT12, CAF130, HPR1, PBS2, PPT1, MET18, MBA1, SCP160, ECM38, RAD26, KAR1, FCY2, YPS1, YDR338C, CWH43, HSP60, SSA4, DIB1, YGL080W, VTH1, BRE4, SWI5, IES6, AAC3, GTT3, TM(CAU)J3, ENA2, PHR1, REV7, MSE1, ALG2, RPL13A, GAL1, YGR130C, SFK1, CLB1, DIA4, RDH54, TIM21, YCL045C, PIB1, HEM12, ZRC1, CAC2, AAH1, SIP1, PES4, ARG5,6, HXT5, UBC6, GDI1, RPS24B, STT3, APA1, SFA1, DOA1, YMR031C, NDE1, PTC7, TQ(UUG)B, ARO7, LOT5, SDL1, TE(UUC)C, HXT11, EMP24, NHA1, PDR3, FAT1, PDH1, SYS1, YMR226C, HRR25, HSP82, CHL1, YPL230W, CLB2, SMX2, ATG19, ELP2, CPR5, SUR4, PRE5, SLH1, AAR2, TPO3, GAT1,

				CHD1, YPR172W, RPL16B, SEF1, CSF1, RIM2, IDS2, SAP4, BIG1, SEC28, POP3, YOL048C, FKS1, ARG2, APS3, MRPS18, CSR2, RPL35A, ALP1, RAD3, GDH1, PNG1, BOI1, VPS24, ALG12, RGD2, MEF1, TOM1, USA1, SGE1, SAK1, RPO21, SED4, PCM1, CLN1, GIM3, YMR118C, PAN6, YBR033W, PUS1, CDC43, PHO12, HUB1, YMR221C, YGL220W, PGM2, GBP2, MCD4, PRP18, FAA2, YPR174C, ADD37, ISU2, IXR1, HYS2, YDR061W, PHO8, RPA49, RPL8A, YNR066C, CAK1, KRS1, MID1, TFG1, ATG18, RPS0B, STV1, GLO2, SEN2, YKL071W, LEM3, SEC17, GAT2, FPR3, PET112, BUD21, COX1, THI4, AMD1, SEC13, HSP12, MBR1, TMA17, STP4, YLR050C, COS9, TRR2, MET28, COQ3, ATG17, APQ12, ATG1, PCA1, SWA2, PSK1, TVP15, SIP18, CCC2, SNR19, RPL6B, ATG3, AIP1, YHC3, VBA1, BNR1, CDC39, YBL054W, HXT15, TR(UCU)K, YOX1, YIP1, RPC40, ORM1, SVS1, VMA6, VAS1, HPT1, SSP1, ISY1, YOR378W, GEA2, MSM1, TH(GUG)E1, YKT6, YNL134C, TAD3, ADE8, LYS2, COX23, SMX3, LYS12, PPZ1, TN(GUU)K, PTP2, DOT6, YSC84, CTK2, YGR111W, APC1, SKG3, UBX3, HOR2, YEA6, YJL045W, INO4, LSM3, OM45, YDR089W, YLR004C, CDC31, ATG7, TG(GCC)M, INM1, CCT4, MLP1, TR(CCG)L, GLY1,

				GPI12, TP(AGG)C, STU1, TPA1, KTR4, SUC2, MAL11, ORC4, GAL3, WRS1, HOF1, ERG2, YEL007W, ATG2, CRN1, CRC1, YCF1, YOR223W, YLR126C, ERG11, IST3, YPT1, RPG1, RPT3, TOS8, RFS1, MAL33, MSH2, BNA4, CRH1, GET1, AST1, RPL5, DPL1, YNL217W, MDY2, YKE4, KEL2, SVF1, ECM31, MSD1, TPK2, MKT1, MTL1, MPA43, ENB1, TLG2, TS(AGA)J, DED1, ARN1, FLC1, FCF2, YFR006W, MET31, TG(GCC)C, MRPL25, SDS3, SNF3, QCR8, SME1, TEL2, COQ1, SMM1, PUS7, IMP1, GLO4, POL5, NHP10, TR(UCU)J2, SAM4, VIK1, ERG5, SKI2, PAC10, UTR1, CDC55, YPL144W, SEC16, DOG2, EFR3, SSL1, ERP3, STB5, TG(UCC)O, TT(UGU)P, YNR024W, UGA2, TPS3, NNF1, SRL2, ATC1, ADE6, VIP1, TRM12, KAR2, ATP8, AVT1, SUT1, PEX11, APD1, YBR238C, SSN2, YIP5, SEC61, UTP5, PCL5, HMS1, RPL9B, ALG14, ARG81, SEC24, UTP21, PRS2, SWD3, AME1, MUP1, NDI1, YSF3, DAL80, JEM1, BET4, BGL2, VAM7, SPA2, RPS24A, DAL5, MDL1, KAP123, MTQ1, CLB4, PEX10, GSH2, ERG3, YCR062W, NMD5, ALG6, RDS3, MSC1, MSB3, HOG1, SNF11, RPL7B, SLS1, RPA135, TOS1, LSM4, CFT1, YRF1-3, RGT1, RPL22B, CCA1, CWC23, RET2, EDC2, NRM1, ADH4, SIR1,

				YOR285W, IRE1, IZH1, FIT1, CTI6, PIN2, SPT7, UPC2, KIP1, AVT2, HNT1, LST4, RPT4, VRG4, LSM5, MNN1, MRPL20, LEU1, TKL1, MRPL40, MST28, RGT2, MMS2, DAD4, CCE1, TSC3, TQ(UUG)E1, MEP2, AXL1, DBF4, FUI1, HXT13, STE7, ZTA1, PPA2, ILM1, YRF1-2, PEP7, TEL1, RSC30, PRM2, SER33, CDC36, PET122, ENT5, HXT2, CLB6, SEC27, WSC2, DER1, DBP5, PAC2, TUB3, NAS2, YLR046C, TR(UCU)M2, YLR247C, PIC2, NOC4, RRP14, PGS1, SCO1, BRR2, EFT2, CHS6, TMA20, PRD1, ULP1, TG(GCC)P2, TR(ACG)D, GPG1, IOC4, MAK3, FIP1, MRPL37, RPL35B, RSC58, DAD2, NOP1, KTR3, NUP170, MCK1, YRO2, NFT1, YGR250C, PHO85, SNX41, SSU1, NTG2, MNT3, YKR070W, TKL2, SWP1, SCEI, CCT6, PRP28, TE(UUC)P, ACO2, YDR307W, YKR096W, RRP5, SSL2, VTA1, TS(AGA)M, MRPL27, YPC1, DAN1, RPO31, HAT1, BEM4, ODC1, YRM1, UBA1, YBR063C, SRB7, YDR128W, PRI2, CDC1, PER1, KAP95, RAD1, MSS116, TV(CAC)D, SEC15, IDH2, HMRA1, PRP8, URA7, YNR063W, FPS1, MVB12, YRF1-7, YOR271C, RPS27B, TA(AGC)K2, NOP13, GTO1, SNM1, PHB2, MND2, TA(UGC)A, GCD6, PEX13, LAG1, YKR104W, CBC2, TAF14, TAL1, RNT1, DLD2, IMD4, CRP1, YHL044W, UBP3,

				ATG5, UTP15, IDI1, STO1, MOD5, DFM1, VMA22, SIP5, GIP4, HPA3, MSS51, SHC1, MTG2, ORC2, GCS1, HAL9, KNH1, TMA10, BSP1, SUE1, ARO2, CDC21, APN1, CYC7, ROT2, GRX5, YBR025C, RRB1, YIL171W, TOP3, INO80, SEN54, SPT21, RGD1, SPT3, RML2, QCR9, HMX1, DGA1, PMU1, SCW4, HHT1, SKI6, PMC1, YNL144C, VPS15, AZR1, ECM7, TAF2, FYV6, YLR412W, PHS1, ALD2, HIS5, APL3, MDM20, GIS1, VPS25, UBP14, HRT3, RCE1, GAP1, SEC12, VMA10, CPR3, YAP3, KCC4, FDH1, PRM7, SOD1, SPT4, PFS2, LPD1, HSP26, YFR011C, URB2, SEC23, DAL4, MRPL39, VPS52, PBP1, OMS1, OSH3, MXR1, GAS4, CIN5, TS(AGA)D3, OMA1, YDR520C, SWH1, SEC9, ADE3, VPS73, YNK1, FET5, YNL274C, PTH1, THP1, PEA2, MET7, YNL168C, NUP157, MAF1, YNL024C, SUI3, OPY2, BOI2, SUA7, MCH5, NBP1, DUS3, NPR1, ISM1, SBE22, RPT6, YKR075C, SEC59, LTE1, CSH1, SLF1, PMT6, FSH2, YLR108C, CTP1, MSS1, TMT1, YOR059C, GIS2, PRS3, ICL1, YIH1, SNU114, VCX1, AFR1, WTM2, GCV2, LSB3, ACN9, RSM10, MTG1, ERB1, ALD3, SUI1, IBD2, PBI2, DAP2, PCL6, ADH3, MRPL8, MTM1, HAS1, TIR1, YCR087C-A, PSE1, GAL4, GCN20, AI4, GRE3, CAR1, RPA190, PCL8, AUS1, RPS29A, PIM1, MIS1, MNS1, GIM4, YPT52,

				GRX4, VAM3, YJL103C, MFA1, ICY1, DBF20, LDB17, ALG7, MRH1, DSE2, AGA2, TYR1, KIN2, RHO4, TV(UAC)B, RNR1, PFK2, YPS3, HHT2, THP2, YPR022C, PTH2, MOB1, RBG2, YLR345W, NUP145, ARX1, DDI1, MDM35, PDS5, PSD2, DCS1, PRR1, RUB1, FMT1, RHR2, YOR262W, TRE1, FLO10, NPT1, MAG1, TAF10, TAZ1, YNL194C, PUF2, ADY3, TQ(UUG)C, HYP2, RAD57, YOR071C, TN(GUU)C, SEC53, CDC4, SGN1, NPL6, LGE1, FPR4, GCD1, ADD66, HSP10, GYP6, GPI16, SWF1, ECM18, NTH1, IML1, RPB7, YNL247W, UTP22, YPR157W, PKR1, FZF1, COX20, OST3, MID2, YRR1, SPT10, SOL4, ELP3, AAT2, PAN5, TG(GCC)J2, SHY1, TG(GCC)O2, EPS1, PKH1, PET111, CIN2, CDC50, MTF1, ATH1, YJL213W, TE(UUC)E1, PEP12, HAM1, UBC8, CCT5, SSF2, NUP192, ASN2, YPR118W, DLS1, MSI1, LST7, YLL054C, RPI1, CDC6, DPM1, NIT3, AST2, SIZ1, SLC1, RAD59, ALD4, DAL82, TE(UUC)M, SNR58, GSC2, LSC2, TRF5, AAP1, RHO2, NOG1, TR(UCU)D, NAM2, RPF1, SUL1, MDM30, CSM4, RAD34, SMD3, YTA7, SMF1, YAL049C, MGM1, RPL24B, TPO2, BRR1, DAL7, TS(AGA)D1, YPR003C, ALG9, YOR390W, FLO9, IMP2, MED2, YOR283W, SFB3, GUK1, PGA3, PPH3, FAS2, YJL171C, YOR008C-A,

				TUB1, TE(UUC)B, MET10, DPS1, TS(UGA)P, SFB2, APT1, HIS2, VAC8, UMP1, SWI1, RTN1, MAK10, BFR1, ILV1, GNP1, DEG1, PFA3, MIG3, RME1, MET2, YEL043W, SLM5, AZF1, ARP10, SIT1, ABP140, ASH1, YDJ1, RIB5, ACA1, CDC26, HOM2, DSE4, LIP1, CNS1, AXL2, GAL2, RFC3, ECM27, EFT1, TG(CCC)O, ESS1, HXK1, UBC1, HAP2, GPI14, YCK2, PRT1, ZWF1, MKC7, YBR220C, YNL176C, NUP100, MUC1, AI2, TR(UCU)M1, POS5, SKM1, ECI1, YLR281C, HOL1, EHD3, HSP42, ESF1, SWD1, PRO1, SSO2, FRE2, STB2, NAT2, CTF18, RRF1, KTR2, CSE4, MPD1, MRPL11, MIC17, TQ(UUG)E2, COG8, RTS2, ALG3, PRK1, TA(UGC)O, MRP51, AHA1, IES4, ASP1, CDC2, YPL191C, REC107, GTR2, DPB2, HIS4, PCL7, TIR3, VPS74, YHR113W, NRD1, SLX8, USE1, MSS2, XBP1, PFK1, PXA2, YNR029C, TH(GUG)K, CUP1-1, ALG5, DNF2, FAR1, COX2, SLM6, TAF6, MSP1, YBR139W, AEP2, SKG6, NCS2, VTC4, ACB1, PMP2, TIF6, YIL064W, HOM6, VMA21, GND1, SPC97, STR3, YDR341C, EMG1, SGF11, THR4, YBR014C, RIB3, ASK10, PIL1, RPL27A, TFB1, YFR055W, TOM71, RPA43, IRR1, TL(GAG)G, CAT8, SWR1, ARF3, HAP1, TMS1, NRG1, NIF3, BUD2, ENT4, TRS120, AVT4, TCM62, DUT1, ALG1, YVC1, SOM1, NOC2, KOG1, STB3, TOM6, AAT1, NDE2,

				TS(UGA)E, HMF1, RPL8B, RPL18A, HHO1, SCH9, RET3, OAC1, SGA1, VHS1, HST4, OYE2, PFK27, RDS1, ECM29, CLB5, PRM5, YOL163W, YCS4, YMR31, SDS24, RPN4, TRS130, MRL1, RPS16B, BAP3, RAD28, ARH1, TQ(UUG)L, VHT1, MRPL50, RPS30B, ABD1, YML081W, MSH4, IRA2, JEN1, MUB1, NMD2, SLI15, FRE4, YMR171C, NCA2, IST1, GAS2, RFA2, RPA14, SNO1, COX12, RGR1, HCH1, UBC9, URA6, SUA5, DTD1, SRL3, YIL166C, TOR1, RRP9, COX7, ADH2, LSB1, FUR4, TSC10, SHM2, NUS1, DIM1, CAP2, SED1, SNG1, HBT1, COS10, SET7, YDL124W, YAL065C, LRP1, SRN2, YOR246C, UTP20, GTO3, RAP1, NPY1, TPP1, ARG3, YFH1, BDH1, TE(UUC)K, ARN2, ARG80, MRM1, MRS1, ASF2, YPL109C, PCS60, FLO8, IPI3, THS1, YFL054C, LIP5, SRP72, FRS2, KTR6
endoplasmic reticulum part	131 out of 1943 genes, 6.7%	302 out of 6348 genes, 4.8%	0.00047	ERG2, YPT1, ALG2, GET1, PMT1, YCL045C, ERV29, SWP1, ERP1, SEC39, YKE4, MNS1, YDR307W, UBC6, ALG7, YPC1, STT3, MPD1, ATF2, EMP24, FLC1, ALG3, ERG9, PER1, SHR5, CPR5, HUT1, USE1, ALG5, SEC11, SEC16, LAG1, BIG1, LAS21, ERP3, VMA21, ERG7, KRE6, CSG2, KAR2, DFM1, SEC61, VMA22, YPR071W, ALG12, ALG14, SEC24, USA1, GPI16, SWF1, SED4, PKR1, PMT2, OST3, CWH41, PDI1, ERF2, ROT2, JEM1, ALG1, EPS1, MCD4,

				CDS1, SAR1, HMX1, ERG3, DGA1, ALG6, WBP1, NSG2, ERP2, YIF1, AQY2, GPI18, NNF2, ERG28, DPM1, PHS1, BST1, RER2, SEC13, MNN9, SEC31, RCE1, SEC12, YLR050C, ERV2, ZRG17, SWA2, GPI11, CSM4, IRE1, IZH1, YPR114W, SEC23, YPR091C, ALG9, YPR003C, PMT3, YIP1, YJL171C, HMG2, ERV41, SEC66, PBN1, LHS1, SUR2, TSC13, PMT5, LCB4, KRE5, RTN1, TSC3, YLL053C, ILM1, SEC59, NUS1, PMT6, FPR2, SCP160, DER1, ERG25, LIP1, YPS1, GPI12, CWH43, GPI19, GPI14, CNE1, GET3, NCP1
intracellular	1665 out of 1943 genes, 85.7%	5229 out of 6348 genes, 82.4%	0.00075	SNX3, SDC25, SOH1, TR(UCU)E, TUF1, SPC19, GCN4, DID2, URM1, BCH1, LOS1, IPP1, MUM2, YIR035C, SFI1, DYS1, AGX1, HIT1, QRI5, BSC2, YAR029W, DNF1, STP3, RTT102, ABF2, CMP2, GCD10, MTO1, TAH1, MDM31, YDL119C, PUS4, CLU1, MNT2, RPL13B, CBP6, DMA1, ATF2, STD1, PET130, IRS4, YPT32, YSC83, TYW1, NTO1, TOS3, TK(CUU)J, MET14, TG(UCC)N, LYS1, TS(AGA)D2, CYM1, CDC42, SEC14, YOR286W, CCP1, HUT1, NAT1, SEN15, YGR207C, YLR419W, ATO3, SSC1, HSP78, FAB1, TRM3, CYR1, ATG11, BUD13, NET1, CSG2, LSG1, ERD1, YPL236C, YTA6, BDF1, URB1, GAL80, YPR071W, GCD7, CMK1, TE(UUC)J, ATP2, RIB4, GRC3, SGS1, GID7, SHE1, RKI1, DOA4,

				SOL3, GPM3, CKS1, RNA1, CAN1, SUV3, YDR161W, HOM3, MSW1, LSP1, HOR7, TIF4632, YOL019W, PET309, TQ(UUG)D3, RRD1, CWC27, SAR1, CCL1, HDA2, CAD1, RET1, YNL045W, ERP2, VHR1, GLO1, IMD2, SNF7, YIA6, MRP2, SNR51, GPI18, SPT20, SUP35, SWI6, RAS2, HSP104, PCK1, AI5_BETA, HEM13, RIM20, RER2, TR(ACG)K, TG(GCC)B, PYK2, MDH2, SEC31, ERG6, FAA4, LST8, SGT2, CBR1, ARC15, TFB3, POP8, REF2, ERV2, NDD1, TIM9, TOM70, CET1, PTC6, URE2, EHT1, GPI11, TI(AAU)L1, DSN1, ARG8, ZAP1, YPR114W, TH(GUG)M, YHB1, FOB1, IDP1, SNF4, INO1, STE12, MRPL3, CSL4, HAT2, ODC2, COT1, RPC37, SEC66, YBR204C, RAD16, GLC8, LHS1, UTR2, PMT5, PET10, RMI1, GOT1, TRP5, ILV5, LAG2, MRP20, YTH1, NAB2, RLM1, SRB2, KTR7, DIN7, UBX6, KAP104, RLI1, MAM1, CDC7, MSN5, PRP5, MST27, POM152, UBP16, POT1, SNR11, KTR1, FPR2, MAK31, DUR1,2, CTR3, FMC1, SSK1, ERG25, ATP11, RFM1, PIH1, HEM3, DIC1, YML018C, GPI19, ATG8, HUG1, ATG26, HTB1, PRS4, SET5, RHO3, YER130C, RSC9, IVY1, PUB1, SUT2, UBR1, ARO4, YAL061W, ISA1, RHO5, YGL039W, RTT106, SWI4, SMC6, TD(GUC)J1, SEC39, TG(CCC)D, NCA3, BUL1, EMP70, MYO1, SGF29, BIO3, MOT3,

				YLH47, RNH203, PSY4, MTR2, VPS38, ECM32, GOS1, SMB1, STF2, ERG9, YMR291W, HCR1, HEK2, MFT1, SHR5, RPS9B, ADE12, TS(AGA)B, YPR011C, LPP1, YPD1, PEX12, RPL9A, LAS21, PAN2, TA(UGC)L, SLY41, TR(UCU)B, LEU4, ERG7, SNT309, KRE6, YTM1, TQ(UUG)D1, MRPL44, RNR2, ECM21, DCC1, FUS2, NOP16, ACF2, HTZ1, ILV2, SER3, SSP120, HSL7, TS(AGA)A, PMT2, ERF2, RRI2, GRX3, PGI1, SRM1, IMG2, RRN6, OSW2, PRO2, YKU80, HPR5, EAF5, PDB1, SAC3, CDS1, CTL1, YBP2, SAD1, YGR043C, GCN1, TAF13, ARO1, ERV15, TS(AGA)L, CLN2, EAF7, IDH1, SKI3, SEN1, SXM1, YHR020W, GCR1, ADY2, SNR56, SAP155, COX17, ERG28, MNP1, KEL3, TGL3, PRB1, SEC18, YJR149W, BST1, TE(CUC)D, NUP84, MNN9, ADE5,7, CTM1, DSS1, SER1, GIP3, SSE2, YOL054W, GAD1, NAS6, ESP1, PRP42, NIP1, YHC1, GDA1, SLM2, BRF1, SSA2, RRI1, SHM1, BUD20, MAM33, BUR6, TOM40, MIA40, VID24, MCH1, YAH1, SNR6, ARD1, TSC13, LCB4, MIF2, FMP46, HSE1, MDJ2, LRO1, TAF4, KRE5, NOP53, TT(AGU)J, MAL23, CCT2, STF1, PDA1, YLL053C, BPH1, TM(CAU)J1, ECM11, RPS0A, NUP85, YLR278C, KAE1, POM34, TSA2, GIS4, SEC21, CYK3, PRP6, RPL31B, SLX9, NUT2,

				ERJ5, TL(UAA)J, BUD8, ARG1, SPO12, COX18, DMA2, URA1, CNE1, AFT1, GET3, NCP1, SNF1, JLP2, STE50, SAM3, PAI3, DOC1, TDP1, SIS2, SSU72, PDR5, MRP10, TRM10, PDE2, PMT1, ERV29, TRA1, ILS1, LEU2, ERP1, YPR004C, SLD5, ARP2, PCI8, USO1, MRPL33, BUD7, NRG2, RPB9, YPR127W, RPC82, TPC1, UTP13, UGA3, PSK2, MNN2, OPT1, TOS4, ESC8, PEX22, GAR1, HST2, YNL320W, COX15, MST1, CHS3, NOP14, KRE33, HOS1, TQ(UUG)D2, STE18, PDR8, ORC5, UBC13, PDS1, YGL157W, NEO1, SEC11, OSH6, NDC1, MSS18, ADO1, MHT1, COX9, CTF13, HAC1, RNR4, KAP122, HEF3, YRF1-6, AFG1, KEX2, COR1, MSH5, NSG1, YDL203C, TS(AGA)E, TOM20, SDH4, MUK1, FBP26, PRX1, ATP3, SEY1, DLD1, AVT7, ZIP1, CDC14, LOC1, URA8, ENT1, DBP2, SDA1, KAP120, YPL229W, MED11, ERG12, MGR1, ILV3, YER184C, CWH41, PDI1, PHD1, YMC2, SPT8, CUP1-2, URA2, BCS1, COY1, POP6, GPD1, HEM1, GUT2, TYS1, LEU9, EST2, VAM6, COP1, DFG10, PUS6, MBF1, WBP1, MSF1, TE(UUC)L, GRE1, NSG2, YBR242W, GIP2, CAR2, YIF1, RRP1, RSR1, CLC1, AQY2, YCL074W, SIW14, VPS20, CDC5, PCL1, RSB1, NNF2, YCR023C, NAB6, SNR61, AIR1, DRS2, ACO1, PPG1, ARO3, DRS1, ZRG17, SNA2, DAL81,

				KRI1, GPH1, GLT1, CUS2, FUN12, MAM3, TIF5, RPS30A, MCM16, SRB8, RNR3, MIH1, RAD50, SCT1, YPR091C, FMS1, YPL141C, PMT3, NUT1, IML3, YHL017W, ERV41, HMG2, MEC1, SPH1, PBN1, IKI1, SUR2, GUS1, SLT2, CSI1, DCS2, SSD1, CDC10, RPN1, YRF1-1, YRB2, SUP45, HFD1, TR(UCU)J1, HSM3, CUP9, GAL83, DBP8, TC(GCA)P1, ICL2, NMA1, CAF130, HPR1, PBS2, PPT1, MET18, MBA1, SCP160, ECM38, RAD26, KAR1, YPS1, CWH43, HSP60, SSA4, DIB1, YGL080W, VTH1, SWI5, IES6, AAC3, GTT3, TM(CAU)J3, ENA2, PHR1, REV7, MSE1, ALG2, RPL13A, GAL1, YGR130C, CLB1, DIA4, RDH54, TIM21, YCL045C, PIB1, HEM12, ZRC1, CAC2, AAH1, SIP1, PES4, ARG5,6, UBC6, GDI1, RPS24B, STT3, APA1, SFA1, DOA1, YMR031C, NDE1, PTC7, TQ(UUG)B, ARO7, LOT5, SDL1, TE(UUC)C, EMP24, PDR3, FAT1, PDH1, SYS1, YMR226C, HRR25, HSP82, CHL1, YPL230W, CLB2, SMX2, ATG19, ELP2, CPR5, SUR4, PRE5, SLH1, AAR2, TPO3, GAT1, CHD1, YPR172W, RPL16B, SEF1, CSF1, RIM2, IDS2, SAP4, BIG1, SEC28, POP3, YOL048C, FKS1, ARG2, APS3, MRPS18, CSR2, RPL35A, RAD3, GDH1, PNG1, VPS24, ALG12, RGD2, MEF1, TOM1, USA1, SAK1, RPO21, SED4, PCM1, CLN1, GIM3, YMR118C, PAN6,

				YBR033W, PUS1, CDC43, PHO12, YMR221C, YGL220W, PGM2, GBP2, MCD4, PRP18, FAA2, YPR174C, ADD37, ISU2, IXR1, HYS2, YDR061W, PHO8, RPA49, RPL8A, CAK1, KRS1, MID1, TFG1, ATG18, RPS0B, STV1, GLO2, SEN2, YKL071W, LEM3, SEC17, GAT2, FPR3, PET112, BUD21, COX1, THI4, AMD1, SEC13, HSP12, MBR1, TMA17, STP4, YLR050C, TRR2, MET28, COQ3, ATG17, APQ12, ATG1, SWA2, PSK1, TVP15, CCC2, SNR19, ATG3, RPL6B, AIP1, YHC3, VBA1, BNR1, CDC39, YBL054W, TR(UCU)K, YOX1, YIP1, RPC40, ORM1, SVS1, VMA6, VAS1, HPT1, ISY1, GEA2, MSM1, TH(GUG)E1, YKT6, YNL134C, TAD3, ADE8, LYS2, COX23, SMX3, LYS12, PPZ1, TN(GUU)K, PTP2, DOT6, YSC84, CTK2, YGR111W, APC1, SKG3, UBX3, HOR2, YEA6, YJL045W, INO4, LSM3, OM45, YLR004C, CDC31, ATG7, TG(GCC)M, INM1, CCT4, MLP1, TR(CCG)L, GLY1, GPI12, TP(AGG)C, STU1, TPA1, KTR4, SUC2, ORC4, GAL3, WRS1, HOF1, ERG2, YEL007W, CRN1, ATG2, CRC1, YCF1, YLR126C, ERG11, IST3, YPT1, RPG1, RPT3, TOS8, RFS1, MAL33, MSH2, BNA4, CRH1, GET1, RPL5, DPL1, YNL217W, MDY2, YKE4, SVF1, ECM31, MSD1, TPK2, MKT1, MPA43, ENB1, TLG2, TS(AGA)J, DED1, ARN1, FLC1, FCF2,

				YFR006W, MET31, TG(GCC)C, MRPL25, SDS3, QCR8, SME1, TEL2, COQ1, SMM1, PUS7, IMP1, GLO4, POL5, NHP10, TR(UCU)J2, SAM4, VIK1, ERG5, SKI2, PAC10, UTR1, CDC55, YPL144W, SEC16, EFR3, DOG2, SSL1, ERP3, STB5, TG(UCC)O, TT(UGU)P, YNR024W, UGA2, TPS3, NNF1, SRL2, ATC1, ADE6, VIP1, TRM12, KAR2, ATP8, AVT1, SUT1, PEX11, APD1, YBR238C, SSN2, YIP5, SEC61, UTP5, PCL5, HMS1, RPL9B, ALG14, ARG81, SEC24, UTP21, PRS2, SWD3, AME1, NDI1, YSF3, DAL80, JEM1, BET4, VAM7, SPA2, RPS24A, MDL1, KAP123, MTQ1, CLB4, PEX10, GSH2, ERG3, YCR062W, NMD5, ALG6, RDS3, MSC1, MSB3, HOG1, SNF11, RPL7B, SLS1, RPA135, TOS1, LSM4, CFT1, YRF1-3, RGT1, RPL22B, CCA1, CWC23, RET2, EDC2, NRM1, ADH4, SIR1, YOR285W, IRE1, IZH1, CTI6, SPT7, KIP1, UPC2, AVT2, HNT1, LST4, RPT4, VRG4, LSM5, MNN1, MRPL20, LEU1, TKL1, MRPL40, MST28, MMS2, DAD4, CCE1, TSC3, TQ(UUG)E1, DBF4, STE7, ZTA1, PPA2, ILM1, YRF1-2, PEP7, TEL1, RSC30, SER33, CDC36, PET122, ENT5, CLB6, SEC27, WSC2, DER1, DBP5, PAC2, TUB3, NAS2, TR(UCU)M2, YLR247C, PIC2, NOC4, RRP14, PGS1, SCO1, BRR2, EFT2, CHS6, TMA20, PRD1, ULP1, TG(GCC)P2, TR(ACG)D,

				GPG1, IOC4, MAK3, FIP1, MRPL37, RPL35B, DAD2, RSC58, NOP1, NUP170, YRO2, YGR250C, PHO85, SNX41, NTG2, MNT3, YKR070W, TKL2, SWP1, SCEI, CCT6, PRP28, TE(UUC)P, ACO2, YDR307W, YKR096W, RRP5, SSL2, VTA1, TS(AGA)M, MRPL27, YPC1, RPO31, HAT1, BEM4, ODC1, YRM1, UBA1, SRB7, YDR128W, PRI2, PER1, KAP95, RAD1, MSS116, TV(CAC)D, SEC15, IDH2, HMRA1, PRP8, URA7, YNR063W, FPS1, MVB12, YRF1-7, YOR271C, RPS27B, TA(AGC)K2, NOP13, GTO1, SNM1, PHB2, MND2, TA(UGC)A, GCD6, PEX13, LAG1, CBC2, TAF14, TAL1, RNT1, DLD2, IMD4, CRP1, UBP3, ATG5, UTP15, IDI1, STO1, MOD5, DFM1, VMA22, SIP5, GIP4, HPA3, MSS51, SHC1, MTG2, ORC2, GCS1, HAL9, TMA10, BSP1, SUE1, ARO2, CDC21, APN1, CYC7, ROT2, GRX5, YBR025C, RRB1, TOP3, INO80, SPT21, SEN54, RGD1, SPT3, RML2, HMX1, QCR9, DGA1, PMU1, HHT1, SKI6, PMC1, YNL144C, VPS15, TAF2, FYV6, YLR412W, ALD2, PHS1, HIS5, APL3, MDM20, GIS1, UBP14, VPS25, HRT3, RCE1, GAP1, SEC12, VMA10, CPR3, YAP3, FDH1, SOD1, SPT4, PFS2, LPD1, HSP26, YFR011C, URB2, SEC23, MRPL39, VPS52, PBP1, OMS1, OSH3, MXR1, CIN5, TS(AGA)D3, OMA1,

				YDR520C, SWH1, ADE3, VPS73, YNK1, FET5, YNL274C, PTH1, THP1, PEA2, MET7, YNL168C, NUP157, MAF1, YNL024C, SUI3, OPY2, BOI2, SUA7, NBP1, DUS3, NPR1, ISM1, SBE22, RPT6, YKR075C, SEC59, LTE1, CSH1, SLF1, PMT6, FSH2, YLR108C, CTP1, MSS1, TMT1, YOR059C, GIS2, ICL1, PRS3, YIH1, SNU114, VCX1, WTM2, GCV2, LSB3, ACN9, RSM10, MTG1, ERB1, ALD3, SUI1, IBD2, PBI2, DAP2, PCL6, ADH3, MRPL8, MTM1, HAS1, YCR087C-A, PSE1, GAL4, GCN20, AI4, GRE3, CAR1, RPA190, PCL8, AUS1, RPS29A, MIS1, PIM1, MNS1, GIM4, YPT52, GRX4, VAM3, YJL103C, ICY1, DBF20, LDB17, ALG7, MRH1, TYR1, RHO4, TV(UAC)B, RNR1, PFK2, HHT2, THP2, YPR022C, PTH2, MOB1, RBG2, YLR345W, NUP145, DDI1, ARX1, MDM35, PDS5, PSD2, DCS1, PRR1, RUB1, FMT1, RHR2, YOR262W, NPT1, MAG1, TAF10, TAZ1, YNL194C, PUF2, ADY3, TQ(UUG)C, RAD57, HYP2, TN(GUU)C, SEC53, CDC4, SGN1, NPL6, LGE1, FPR4, GCD1, ADD66, HSP10, GYP6, GPI16, SWF1, ECM18, NTH1, YNL247W, IML1, RPB7, UTP22, PKR1, FZF1, COX20, OST3, YRR1, SPT10, SOL4, ELP3, AAT2, PAN5, TG(GCC)J2, SHY1, TG(GCC)O2, EPS1, PKH1, PET111, CIN2, CDC50, MTF1, ATH1, YJL213W, TE(UUC)E1, PEP12, HAM1,

				UBC8, CCT5, SSF2, NUP192, ASN2, YPR118W, DLS1, MSI1, LST7, YLL054C, RPI1, CDC6, DPM1, NIT3, AST2, SIZ1, SLC1, RAD59, ALD4, DAL82, TE(UUC)M, SNR58, GSC2, LSC2, TRF5, AAP1, RHO2, NOG1, TR(UCU)D, NAM2, RPF1, MDM30, CSM4, RAD34, SMD3, YTA7, SMF1, YAL049C, MGM1, RPL24B, TPO2, BRR1, DAL7, TS(AGA)D1, YPR003C, ALG9, IMP2, MED2, YOR283W, SFB3, GUK1, PGA3, PPH3, FAS2, YJL171C, TUB1, TE(UUC)B, DPS1, MET10, TS(UGA)P, SFB2, APT1, HIS2, VAC8, UMP1, SWI1, RTN1, MAK10, BFR1, ILV1, GNP1, DEG1, PFA3, MIG3, RME1, MET2, YEL043W, SLM5, AZF1, ARP10, SIT1, ABP140, ASH1, YDJ1, ACA1, CDC26, HOM2, CNS1, AXL2, LIP1, RFC3, EFT1, TG(CCC)O, ESS1, HXK1, UBC1, HAP2, GPI14, PRT1, ZWF1, YNL176C, NUP100, AI2, TR(UCU)M1, POS5, ECI1, YLR281C, HOL1, EHD3, HSP42, ESF1, SWD1, PRO1, SSO2, STB2, NAT2, CTF18, RRF1, KTR2, CSE4, MPD1, MRPL11, MIC17, TQ(UUG)E2, COG8, RTS2, ALG3, PRK1, TA(UGC)O, MRP51, AHA1, IES4, ASP1, CDC2, YPL191C, REC107, GTR2, DPB2, HIS4, PCL7, VPS74, YHR113W, NRD1, SLX8, USE1, MSS2, XBP1, PFK1, PXA2, YNR029C, TH(GUG)K, CUP1-1, ALG5, FAR1, COX2, SLM6, TAF6,

				MSP1, YBR139W, AEP2, SKG6, NCS2, ACB1, VTC4, TIF6, YIL064W, HOM6, VMA21, GND1, SPC97, STR3, YDR341C, EMG1, SGF11, THR4, YBR014C, RIB3, ASK10, PIL1, RPL27A, TFB1, YFR055W, TOM71, RPA43, IRR1, TL(GAG)G, CAT8, SWR1, ARF3, HAP1, TMS1, NRG1, NIF3, BUD2, ENT4, TRS120, AVT4, TCM62, DUT1, ALG1, YVC1, SOM1, NOC2, KOG1, STB3, TOM6, AAT1, NDE2, TS(UGA)E, HMF1, RPL8B, RPL18A, HHO1, SCH9, RET3, OAC1, SGA1, VHS1, HST4, OYE2, PFK27, RDS1, ECM29, CLB5, YCS4, YMR31, SDS24, RPN4, TRS130, MRL1, RPS16B, BAP3, RAD28, ARH1, TQ(UUG)L, MRPL50, RPS30B, ABD1, YML081W, MSH4, IRA2, JEN1, MUB1, NMD2, SLI15, YMR171C, NCA2, IST1, GAS2, RFA2, RPA14, SNO1, COX12, RGR1, HCH1, UBC9, URA6, SUA5, DTD1, SRL3, TOR1, RRP9, COX7, ADH2, LSB1, TSC10, SHM2, NUS1, DIM1, CAP2, SED1, HBT1, COS10, SET7, YDL124W, LRP1, SRN2, YOR246C, UTP20, GTO3, RAP1, NPY1, TPP1, ARG3, YFH1, BDH1, TE(UUC)K, ARN2, ARG80, MRM1, MRS1, ASF2, YPL109C, PCS60, FLO8, IPI3, THS1, LIP5, SRP72, FRS2
nuclear envelope-endoplasmic reticulum network	125 out of 1943 genes, 6.4%	288 out of 6348 genes, 4.5%	0.00081	ERG2, SAR1, CDS1, MCD4, ERG3, HMX1, DGA1, YPT1, ALG6, ALG2, WBP1, GET1, NSG2, YIF1, ERP2, PMT1, AQY2, YCL045C, ERV29, SWP1, ERP1,

				SEC39, GPI18, YKE4, MNS1, YDR307W, NNF2, ERG28, DPM1, PHS1, BST1, UBC6, RER2, MNN9, SEC13, ALG7, YPC1, SEC31, STT3, RCE1, SEC12, ATF2, YLR050C, EMP24, ERV2, FLC1, ALG3, ZRG17, SWA2, GPI11, IRE1, CSM4, ERG9, IZH1, PER1, YPR114W, SEC23, SHR5, YPR091C, YPR003C, ALG9, HUT1, USE1, PMT3, YIP1, ERV41, HMG2, YJL171C, ALG5, SEC66, SEC11, PBN1, SUR2, TSC13, LCB4, SEC16, PMT5, LAG1, BIG1, LAS21, THP1, ERP3, TSC3, RTN1, BFR1, VMA21, ERG7, KRE6, CSG2, YLL053C, ILM1, SEC59, DFM1, NUS1, SEC61, VMA22, YPR071W, PMT6, ALG12, ALG14, SEC24, FPR2, SCP160, DER1, USA1, GPI16, SWF1, SED4, PKR1, PMT2, LIP1, ERG25, YPS1, OST3, CWH41, ERF2, GPI12, CWH43, JEM1, ALG1, GPI19, CNE1, GPI14, NCP1, EPS1
endoplasmic reticulum membrane	123 out of 1943 genes, 6.3%	284 out of 6348 genes, 4.5%	0.00113	ERG2, SAR1, CDS1, MCD4, ERG3, HMX1, DGA1, YPT1, ALG6, ALG2, WBP1, GET1, NSG2, YIF1, ERP2, PMT1, AQY2, YCL045C, ERV29, SWP1, ERP1, SEC39, GPI18, YKE4, MNS1, YDR307W, NNF2, ERG28, DPM1, PHS1, BST1, UBC6, RER2, MNN9, SEC13, ALG7, YPC1, SEC31, STT3, RCE1, SEC12, ATF2, YLR050C, EMP24, ERV2, FLC1, ALG3, ZRG17, SWA2, GPI11, IRE1, CSM4, ERG9, IZH1, PER1, YPR114W, SEC23, SHR5, YPR091C,

				YPR003C, ALG9, HUT1, USE1, PMT3, YIP1, ERV41, HMG2, YJL171C, ALG5, SEC66, SEC11, PBN1, SUR2, TSC13, LCB4, SEC16, PMT5, LAG1, BIG1, LAS21, ERP3, TSC3, RTN1, VMA21, ERG7, KRE6, CSG2, YLL053C, ILM1, SEC59, DFM1, NUS1, SEC61, VMA22, YPR071W, PMT6, ALG12, ALG14, SEC24, FPR2, SCP160, DER1, USA1, GPI16, SWF1, SED4, PKR1, PMT2, LIP1, ERG25, YPS1, OST3, CWH41, ERF2, GPI12, CWH43, JEM1, ALG1, GPI19, CNE1, GPI14, NCP1, EPS1
membrane	628 out of 1943 genes, 32.3%	1804 out of 6348 genes, 28.4%	0.00137	ERG2, SNX3, ATG2, CRC1, ERG11, YOR223W, YCF1, YPT1, DID2, CRH1, BNA4, GET1, BCH1, AST1, DFG5, QRI5, BSC2, YAR029W, DNF1, YKE4, AGA1, MDM31, YDL119C, MTL1, TLG2, ENB1, MNT2, ATF2, STD1, ARN1, FLC1, YFR006W, YPT32, YSC83, SNF3, QCR8, COQ1, CWP1, ITR2, CDC42, SEC14, IMP1, HUT1, SEN15, ATO3, SSC1, FAB1, SEC16, ERP3, CYR1, ATG11, DAP1, CSG2, ATP8, AVT1, ERD1, PEX11, YPL236C, YBR238C, YIP5, SEC61, YPR071W, ALG14, ATP2, SEC24, MUP1, NDI1, CAN1, JEM1, VAM7, LSP1, HOR7, DAL5, MDL1, KAP123, YOL019W, ENA5, PHO84, PET309, SAR1, PEX10, ERG3, YCR062W, NMD5, ALG6, ERP2, SLS1, ENA1, SNF7, YIA6, GPI18, RAS2, RET2, AI5_BETA, HEM13, RER2, SEC31, ERG6, LST8, CBR1,

				ARC15, ERV2, TIM9, TOM70, EHT1, YOR285W, GPI11, IRE1, IZH1, YPR114W, FIT1, PIN2, SNF4, AVT2, LST4, ODC2, COT1, FLO1, SEC66, FRE3, VRG4, UTR2, MNN1, PMT5, PET10, GOT1, MST28, RGT2, BAP2, CCE1, TSC3, MEP2, AXL1, KTR7, FUI1, HXT13, ILM1, PEP7, PRM2, RLI1, PET122, MST27, POM152, UBP16, ENT5, HXT2, SEC27, KTR1, FPR2, WSC2, CTR3, DER1, DBP5, FEN2, YLR046C, ERG25, PIC2, DIC1, SCO1, YML018C, GPI19, ATG8, ATG26, CHS6, ULP1, RHO3, IVY1, KTR3, NUP170, YRO2, DAL3, NFT1, SSU1, RHO5, MNT3, VTH2, SWP1, SCEI, SEC39, BUL1, EMP70, YDR307W, VTA1, YLH47, YPC1, MNN4, DAN1, MTR2, MFA2, IPT1, VPS38, ODC1, EXG2, GOS1, YBR063C, ERG9, STF2, YDR128W, CDC1, PER1, KAP95, AGP1, SHR5, SEC15, FPS1, MVB12, YOR271C, YPR011C, LPP1, PHB2, PEX12, PEX13, YKR104W, LAG1, LAS21, SLY41, ERG7, YHL044W, KRE6, ATG5, DFM1, HXT16, VMA22, MSS51, FLO5, MTG2, SHC1, BSP1, TIR4, PMT2, STE4, ERF2, CYC7, OSW2, FRE8, YIL171W, YBR284W, SEN54, SAC3, RAX1, TAT1, CDS1, QCR9, HMX1, DGA1, PMC1, FCY22, VPS15, AZR1, ERV15, ECM7, SXM1, ADY2, ERG28, PHS1, TGL3, SEC18, APL3, BST1, NUP84, MNN9, VPS25,

				RCE1, GAP1, SEC12, VMA10, PRM7, DTR1, QDR2, GDA1, SLM2, SEC23, DAL4, SSA2, HKR1, VPS52, PHO89, OMS1, GAS4, TOM40, MIA40, OMA1, SEC9, VID24, VPS73, MCH1, TSC13, FET5, LCB4, THP1, HSE1, KRE1, MDJ2, LRO1, NUP157, OPY2, MCH5, STF1, YLL053C, SEC59, NUP85, POM34, CSH1, PMT6, GIS4, CTP1, MSS1, SEC21, YOR059C, ERJ5, VCX1, BUD8, COX18, URA1, MTG1, CNE1, GET3, NCP1, SNF1, SAM3, DAP2, MTM1, TIR1, PDR5, ITR1, AI4, PMT1, YPS6, ERV29, ERP1, AUS1, MNS1, YPT52, USO1, BUD7, VAM3, MFA1, ICY1, ALG7, MRH1, DSE2, TPC1, MNN2, KIN2, RHO4, OPT1, PEX22, YPS3, COX15, YNL320W, CHS3, PTH2, NUP145, DDI1, YPR117W, STE18, TAT2, PSD2, NEO1, SEC11, NDC1, COX9, KAP122, AFG1, KEX2, COR1, TRE1, NSG1, TOM20, SDH4, ATP3, DLD1, YNL194C, SEY1, TAZ1, AVT7, ADY3, YOR071C, ENT1, HXT9, KAP120, YFL067W, HUR1, GPI16, SWF1, IML1, MGR1, YPR157W, PKR1, OST3, COX20, CWH41, MID2, YMC2, URA2, BCS1, COY1, GUT2, SHY1, VAM6, EPS1, COP1, DFG10, PET111, CDC50, WBP1, NSG2, PEP12, YIF1, RSR1, NUP192, AQY2, CLC1, LST7, VPS20, YLL054C, CDC6, RSB1, NNF2, YCR023C, DPM1, SLC1, GSC2, DRS2, RHO2,

				PTR2, ZRG17, SUL1, SNA2, CSM4, MAM3, SMF1, MGM1, TPO2, SCT1, YPR091C, YPR003C, ALG9, YOR390W, FLO9, PMT3, IMP2, SFB3, YOR1, YHL017W, PGA3, ERV41, YJL171C, HMG2, YOR008C-A, PBN1, SUR2, SFB2, CDC10, VAC8, YRB2, RTN1, HFD1, BFR1, GNP1, PFA3, HXT12, SIT1, MBA1, SCP160, ECM38, FCY2, AXL2, LIP1, YPS1, GAL2, YDR338C, ECM27, CWH43, BRE4, VTH1, AAC3, YCK2, GPI14, MKC7, GTT3, YBR220C, ENA2, YNL176C, NUP100, MUC1, AI2, SKM1, ALG2, HOL1, SFK1, FRE2, SSO2, YCL045C, TIM21, PIB1, ZRC1, KTR2, HXT5, UBC6, STT3, COG8, EMP24, HXT11, NHA1, ALG3, FAT1, PDH1, SYS1, HRR25, GTR2, ATG19, SUR4, MSS2, USE1, PXA2, TPO3, ALG5, DNF2, FAR1, COX2, CSF1, RIM2, MSP1, BIG1, SKG6, SEC28, YOL048C, FKS1, APS3, VTC4, PMP2, VMA21, ALP1, PIL1, VPS24, ALG12, TOM71, SGE1, USA1, TMS1, SED4, AVT4, TCM62, ALG1, YMR118C, YVC1, SOM1, KOG1, TOM6, YMR221C, MCD4, YPR174C, YDR061W, SCH9, PHO8, OAC1, RET3, YNR066C, MID1, ATG18, STV1, SEN2, LEM3, PRM5, YOL163W, SEC17, COX1, SEC13, HSP12, MRL1, BAP3, YLR050C, COS9, COQ3, APQ12, PCA1, ATG1, SWA2, TVP15, ARH1, VHT1, CCC2, VBA1, YHC3, JEN1, HXT15, YIP1, ORM1,

				FRE4, YMR171C, NCA2, VMA6, SSP1, GAS2, COX12, YOR378W, GEA2, YIL166C, YKT6, TOR1, COX7, PPZ1, FUR4, TSC10, NUS1, SKG3, SED1, SNG1, COS10, YAL065C, YEA6, SRN2, YOR246C, YJL045W, OM45, YDR089W, ATG7, CDC31, YLR004C, MLP1, ARN2, GPI12, PCS60, KTR4, YFL054C, MAL11, KTR6
intracellular part	1652 out of 1943 genes, 85.0%	5191 out of 6348 genes, 81.8%	0.00165	SNX3, SOH1, TR(UCU)E, TUF1, SPC19, GCN4, DID2, URM1, BCH1, LOS1, IPP1, MUM2, YIR035C, SFI1, DYS1, AGX1, HIT1, QRI5, BSC2, YAR029W, DNF1, STP3, RTT102, ABF2, CMP2, GCD10, MTO1, TAH1, MDM31, YDL119C, PUS4, CLU1, MNT2, RPL13B, CBP6, DMA1, ATF2, STD1, PET130, IRS4, YPT32, YSC83, TYW1, NTO1, TOS3, TK(CUU)J, MET14, TG(UCC)N, LYS1, TS(AGA)D2, CYM1, SEC14, YOR286W, CCP1, HUT1, NAT1, SEN15, YGR207C, YLR419W, ATO3, SSC1, HSP78, FAB1, TRM3, CYR1, ATG11, BUD13, NET1, CSG2, LSG1, ERD1, YPL236C, YTA6, BDF1, URB1, GAL80, YPR071W, GCD7, CMK1, TE(UUC)J, ATP2, RIB4, GRC3, SGS1, GID7, SHE1, RKI1, DOA4, SOL3, GPM3, CKS1, RNA1, CAN1, SUV3, YDR161W, HOM3, MSW1, LSP1, HOR7, TIF4632, YOL019W, PET309, TQ(UUG)D3, RRD1, CWC27, SAR1, CCL1, HDA2, CAD1, RET1, YNL045W, ERP2, VHR1, GLO1, IMD2, SNF7, YIA6,

				MRP2, SNR51, GPI18, SPT20, SUP35, SWI6, HSP104, PCK1, AI5_BETA, HEM13, RIM20, RER2, TR(ACG)K, TG(GCC)B, PYK2, MDH2, SEC31, ERG6, FAA4, LST8, SGT2, CBR1, ARC15, TFB3, POP8, REF2, ERV2, NDD1, TIM9, TOM70, CET1, PTC6, URE2, EHT1, GPI11, TI(AAU)L1, DSN1, ARG8, ZAP1, YPR114W, TH(GUG)M, YHB1, FOB1, IDP1, SNF4, INO1, STE12, MRPL3, CSL4, HAT2, ODC2, COT1, RPC37, SEC66, YBR204C, RAD16, GLC8, LHS1, UTR2, PMT5, PET10, RMI1, GOT1, TRP5, ILV5, LAG2, MRP20, YTH1, NAB2, RLM1, SRB2, KTR7, DIN7, UBX6, KAP104, RLI1, MAM1, CDC7, MSN5, PRP5, MST27, POM152, UBP16, POT1, SNR11, KTR1, FPR2, MAK31, DUR1,2, CTR3, FMC1, SSK1, ERG25, ATP11, RFM1, PIH1, HEM3, DIC1, YML018C, GPI19, ATG8, HUG1, ATG26, HTB1, PRS4, SET5, YER130C, RSC9, IVY1, PUB1, SUT2, UBR1, ARO4, YAL061W, ISA1, RHO5, YGL039W, RTT106, SWI4, SMC6, TD(GUC)J1, SEC39, TG(CCC)D, NCA3, BUL1, EMP70, MYO1, SGF29, BIO3, MOT3, YLH47, RNH203, PSY4, MTR2, VPS38, ECM32, GOS1, SMB1, STF2, ERG9, YMR291W, HCR1, HEK2, MFT1, SHR5, RPS9B, ADE12, TS(AGA)B, YPR011C, LPP1, YPD1, PEX12, RPL9A, LAS21, PAN2, TA(UGC)L, SLY41,

				TR(UCU)B, LEU4, ERG7, SNT309, KRE6, YTM1, TQ(UUG)D1, MRPL44, RNR2, ECM21, DCC1, FUS2, NOP16, ACF2, HTZ1, ILV2, SER3, SSP120, HSL7, TS(AGA)A, PMT2, ERF2, RRI2, GRX3, PGI1, SRM1, IMG2, RRN6, OSW2, PRO2, YKU80, HPR5, EAF5, PDB1, SAC3, CDS1, CTL1, YBP2, SAD1, YGR043C, GCN1, TAF13, ARO1, ERV15, TS(AGA)L, CLN2, EAF7, IDH1, SKI3, SEN1, SXM1, YHR020W, GCR1, ADY2, SNR56, SAP155, COX17, ERG28, MNP1, KEL3, TGL3, PRB1, SEC18, YJR149W, BST1, TE(CUC)D, NUP84, MNN9, ADE5,7, CTM1, DSS1, SER1, GIP3, SSE2, YOL054W, GAD1, NAS6, ESP1, PRP42, NIP1, YHC1, GDA1, SLM2, BRF1, SSA2, RRI1, SHM1, BUD20, MAM33, BUR6, TOM40, MIA40, VID24, MCH1, YAH1, SNR6, ARD1, TSC13, LCB4, MIF2, FMP46, HSE1, MDJ2, LRO1, TAF4, KRE5, NOP53, TT(AGU)J, MAL23, CCT2, STF1, PDA1, YLL053C, BPH1, TM(CAU)J1, ECM11, RPS0A, NUP85, YLR278C, KAE1, POM34, TSA2, GIS4, SEC21, CYK3, PRP6, RPL31B, SLX9, NUT2, ERJ5, TL(UAA)J, BUD8, ARG1, SPO12, COX18, DMA2, URA1, CNE1, AFT1, GET3, NCP1, SNF1, JLP2, STE50, SAM3, PAI3, DOC1, TDP1, SIS2, SSU72, PDR5, MRP10, TRM10, PDE2, PMT1, ERV29, TRA1, ILS1, LEU2, ERP1,

				YPR004C, SLD5, ARP2, PCI8, USO1, MRPL33, BUD7, NRG2, RPB9, YPR127W, RPC82, TPC1, UTP13, UGA3, PSK2, MNN2, OPT1, TOS4, ESC8, PEX22, GAR1, HST2, YNL320W, COX15, MST1, CHS3, NOP14, KRE33, HOS1, TQ(UUG)D2, STE18, PDR8, ORC5, UBC13, PDS1, YGL157W, NEO1, SEC11, OSH6, NDC1, MSS18, ADO1, MHT1, COX9, CTF13, HAC1, RNR4, KAP122, HEF3, YRF1-6, AFG1, KEX2, COR1, MSH5, NSG1, YDL203C, TS(AGA)E, TOM20, SDH4, MUK1, FBP26, PRX1, ATP3, SEY1, DLD1, AVT7, ZIP1, CDC14, LOC1, URA8, ENT1, DBP2, SDA1, KAP120, YPL229W, MED11, ERG12, MGR1, ILV3, YER184C, CWH41, PDI1, PHD1, YMC2, SPT8, CUP1-2, URA2, BCS1, COY1, POP6, GPD1, HEM1, GUT2, TYS1, LEU9, EST2, VAM6, COP1, DFG10, PUS6, MBF1, WBP1, MSF1, TE(UUC)L, GRE1, NSG2, YBR242W, GIP2, CAR2, YIF1, RRP1, CLC1, AQY2, YCL074W, SIW14, VPS20, CDC5, PCL1, RSB1, NNF2, YCR023C, NAB6, SNR61, AIR1, DRS2, ACO1, PPG1, ARO3, DRS1, ZRG17, SNA2, DAL81, KRI1, GPH1, GLT1, CUS2, FUN12, MAM3, TIF5, RPS30A, MCM16, SRB8, RNR3, MIH1, RAD50, SCT1, YPR091C, FMS1, YPL141C, PMT3, NUT1, IML3, YHL017W, ERV41, HMG2, MEC1, SPH1, PBN1, IKI1, SUR2, GUS1, SLT2, CSI1,

				DCS2, SSD1, CDC10, RPN1, YRF1-1, YRB2, SUP45, HFD1, TR(UCU)J1, HSM3, CUP9, GAL83, DBP8, TC(GCA)P1, ICL2, NMA1, CAF130, HPR1, PBS2, PPT1, MET18, MBA1, SCP160, ECM38, RAD26, KAR1, YPS1, CWH43, HSP60, SSA4, DIB1, YGL080W, VTH1, SWI5, IES6, AAC3, GTT3, TM(CAU)J3, ENA2, PHR1, REV7, MSE1, ALG2, RPL13A, GAL1, YGR130C, CLB1, DIA4, RDH54, TIM21, YCL045C, PIB1, HEM12, ZRC1, CAC2, AAH1, SIP1, PES4, ARG5,6, UBC6, GDI1, RPS24B, STT3, APA1, SFA1, DOA1, YMR031C, NDE1, PTC7, TQ(UUG)B, ARO7, LOT5, SDL1, TE(UUC)C, EMP24, PDR3, FAT1, PDH1, SYS1, YMR226C, HRR25, HSP82, CHL1, CLB2, SMX2, ATG19, ELP2, CPR5, SUR4, PRE5, SLH1, AAR2, TPO3, GAT1, CHD1, YPR172W, RPL16B, SEF1, CSF1, RIM2, IDS2, SAP4, BIG1, SEC28, POP3, YOL048C, FKS1, ARG2, APS3, MRPS18, CSR2, RPL35A, RAD3, GDH1, PNG1, VPS24, ALG12, RGD2, MEF1, TOM1, USA1, SAK1, RPO21, SED4, PCM1, CLN1, GIM3, YMR118C, PAN6, YBR033W, PUS1, CDC43, PHO12, YMR221C, YGL220W, PGM2, GBP2, MCD4, PRP18, FAA2, YPR174C, ADD37, ISU2, IXR1, HYS2, YDR061W, PHO8, RPA49, RPL8A, CAK1, KRS1, MID1, TFG1, ATG18, RPS0B, STV1, GLO2, SEN2, YKL071W,

				LEM3, SEC17, GAT2, FPR3, PET112, BUD21, COX1, THI4, AMD1, SEC13, HSP12, MBR1, TMA17, STP4, YLR050C, TRR2, MET28, COQ3, ATG17, APQ12, ATG1, SWA2, PSK1, TVP15, CCC2, SNR19, ATG3, RPL6B, AIP1, YHC3, VBA1, BNR1, CDC39, YBL054W, TR(UCU)K, YOX1, YIP1, RPC40, ORM1, SVS1, VMA6, VAS1, HPT1, ISY1, GEA2, MSM1, TH(GUG)E1, YKT6, YNL134C, TAD3, ADE8, LYS2, COX23, SMX3, LYS12, PPZ1, TN(GUU)K, PTP2, DOT6, YSC84, CTK2, YGR111W, APC1, SKG3, UBX3, HOR2, YEA6, YJL045W, INO4, LSM3, OM45, YLR004C, CDC31, ATG7, TG(GCC)M, INM1, CCT4, MLP1, TR(CCG)L, GLY1, GPI12, TP(AGG)C, STU1, TPA1, KTR4, SUC2, ORC4, GAL3, WRS1, HOF1, ERG2, YEL007W, CRN1, ATG2, CRC1, YCF1, YLR126C, ERG11, IST3, YPT1, RPG1, RPT3, TOS8, RFS1, MAL33, MSH2, BNA4, CRH1, GET1, RPL5, DPL1, YNL217W, MDY2, YKE4, SVF1, ECM31, MSD1, TPK2, MKT1, MPA43, ENB1, TLG2, TS(AGA)J, DED1, ARN1, FLC1, FCF2, YFR006W, MET31, TG(GCC)C, MRPL25, SDS3, QCR8, SME1, TEL2, COQ1, SMM1, PUS7, IMP1, GLO4, POL5, NHP10, TR(UCU)J2, SAM4, VIK1, ERG5, SKI2, PAC10, UTR1, CDC55, YPL144W, SEC16, EFR3, DOG2, SSL1, ERP3, STB5, TG(UCC)O, TT(UGU)P,

				YNR024W, UGA2, TPS3, NNF1, SRL2, ATC1, ADE6, VIP1, TRM12, KAR2, ATP8, AVT1, SUT1, PEX11, APD1, YBR238C, SSN2, YIP5, SEC61, UTP5, PCL5, HMS1, RPL9B, ALG14, ARG81, SEC24, UTP21, PRS2, SWD3, AME1, NDI1, YSF3, DAL80, JEM1, BET4, VAM7, SPA2, RPS24A, MDL1, KAP123, MTQ1, CLB4, PEX10, ERG3, YCR062W, NMD5, ALG6, RDS3, MSC1, MSB3, HOG1, SNF11, RPL7B, SLS1, RPA135, TOS1, LSM4, CFT1, YRF1-3, RGT1, RPL22B, CCA1, CWC23, RET2, EDC2, NRM1, ADH4, SIR1, YOR285W, IRE1, IZH1, CTI6, SPT7, KIP1, UPC2, AVT2, HNT1, LST4, RPT4, VRG4, LSM5, MNN1, MRPL20, LEU1, TKL1, MRPL40, MST28, MMS2, DAD4, CCE1, TSC3, TQ(UUG)E1, DBF4, STE7, ZTA1, PPA2, ILM1, YRF1-2, PEP7, TEL1, RSC30, SER33, CDC36, PET122, ENT5, CLB6, SEC27, WSC2, DER1, DBP5, PAC2, TUB3, NAS2, TR(UCU)M2, YLR247C, PIC2, NOC4, RRP14, PGS1, SCO1, BRR2, EFT2, CHS6, TMA20, PRD1, ULP1, TG(GCC)P2, TR(ACG)D, GPG1, IOC4, MAK3, FIP1, MRPL37, RPL35B, DAD2, RSC58, NOP1, NUP170, YRO2, YGR250C, PHO85, SNX41, NTG2, MNT3, YKR070W, TKL2, SWP1, SCEI, CCT6, PRP28, TE(UUC)P, ACO2, YDR307W, YKR096W, RRP5, SSL2, VTA1,

				TS(AGA)M, MRPL27, YPC1, RPO31, HAT1, BEM4, ODC1, YRM1, UBA1, SRB7, YDR128W, PRI2, PER1, KAP95, RAD1, MSS116, TV(CAC)D, SEC15, IDH2, HMRA1, PRP8, URA7, YNR063W, FPS1, MVB12, YRF1-7, YOR271C, RPS27B, TA(AGC)K2, NOP13, GTO1, SNM1, PHB2, MND2, TA(UGC)A, GCD6, PEX13, LAG1, CBC2, TAF14, TAL1, RNT1, DLD2, IMD4, CRP1, UBP3, ATG5, UTP15, IDI1, STO1, MOD5, DFM1, VMA22, SIP5, GIP4, HPA3, MSS51, SHC1, MTG2, ORC2, GCS1, HAL9, TMA10, BSP1, SUE1, ARO2, CDC21, APN1, CYC7, ROT2, GRX5, YBR025C, RRB1, TOP3, INO80, SPT21, SEN54, RGD1, SPT3, RML2, HMX1, QCR9, DGA1, PMU1, HHT1, SKI6, PMC1, YNL144C, VPS15, TAF2, FYV6, YLR412W, ALD2, PHS1, APL3, MDM20, GIS1, UBP14, VPS25, HRT3, RCE1, GAP1, SEC12, VMA10, CPR3, YAP3, FDH1, SOD1, SPT4, PFS2, LPD1, HSP26, YFR011C, URB2, SEC23, MRPL39, VPS52, PBP1, OMS1, OSH3, MXR1, CIN5, TS(AGA)D3, OMA1, YDR520C, SWH1, ADE3, VPS73, YNK1, FET5, YNL274C, PTH1, THP1, PEA2, MET7, YNL168C, NUP157, MAF1, YNL024C, SUI3, OPY2, BOI2, SUA7, NBP1, DUS3, NPR1, ISM1, SBE22, RPT6, YKR075C, SEC59, LTE1, CSH1, SLF1, PMT6, FSH2, YLR108C, CTP1, MSS1,

				TMT1, YOR059C, GIS2, ICL1, PRS3, YIH1, SNU114, VCX1, WTM2, GCV2, LSB3, ACN9, RSM10, MTG1, ERB1, ALD3, SUI1, IBD2, PBI2, DAP2, PCL6, ADH3, MRPL8, MTM1, HAS1, YCR087C-A, PSE1, GAL4, GCN20, AI4, GRE3, CAR1, RPA190, PCL8, AUS1, RPS29A, MIS1, PIM1, MNS1, GIM4, YPT52, GRX4, VAM3, YJL103C, ICY1, DBF20, LDB17, ALG7, MRH1, TYR1, TV(UAC)B, RNR1, PFK2, HHT2, THP2, YPR022C, PTH2, MOB1, RBG2, YLR345W, NUP145, DDI1, ARX1, MDM35, PDS5, PSD2, DCS1, PRR1, RUB1, FMT1, RHR2, YOR262W, NPT1, MAG1, TAF10, TAZ1, YNL194C, PUF2, ADY3, TQ(UUG)C, RAD57, HYP2, TN(GUU)C, SEC53, CDC4, SGN1, NPL6, LGE1, FPR4, GCD1, ADD66, HSP10, GYP6, GPI16, SWF1, ECM18, NTH1, YNL247W, IML1, RPB7, UTP22, PKR1, FZF1, COX20, OST3, YRR1, SPT10, SOL4, ELP3, AAT2, PAN5, TG(GCC)J2, SHY1, TG(GCC)O2, EPS1, PKH1, PET111, CIN2, CDC50, MTF1, ATH1, YJL213W, TE(UUC)E1, PEP12, HAM1, UBC8, CCT5, SSF2, NUP192, ASN2, YPR118W, DLS1, MSI1, LST7, YLL054C, RPI1, CDC6, DPM1, NIT3, SIZ1, AST2, SLC1, RAD59, ALD4, DAL82, TE(UUC)M, SNR58, GSC2, LSC2, TRF5, AAP1, NOG1, TR(UCU)D, NAM2, RPF1, MDM30, CSM4, RAD34, SMD3,

				YTA7, SMF1, YAL049C, MGM1, RPL24B, TPO2, BRR1, DAL7, TS(AGA)D1, YPR003C, ALG9, IMP2, MED2, SFB3, YOR283W, GUK1, PGA3, PPH3, FAS2, YJL171C, TUB1, TE(UUC)B, DPS1, MET10, TS(UGA)P, SFB2, APT1, VAC8, UMP1, SWI1, RTN1, MAK10, BFR1, ILV1, GNP1, DEG1, PFA3, MIG3, RME1, MET2, YEL043W, SLM5, AZF1, ARP10, SIT1, ABP140, ASH1, YDJ1, ACA1, CDC26, HOM2, CNS1, AXL2, LIP1, RFC3, EFT1, TG(CCC)O, ESS1, HXK1, UBC1, HAP2, GPI14, PRT1, ZWF1, YNL176C, NUP100, AI2, TR(UCU)M1, POS5, ECI1, YLR281C, HOL1, EHD3, HSP42, ESF1, SWD1, PRO1, SSO2, STB2, NAT2, CTF18, RRF1, KTR2, CSE4, MPD1, MRPL11, MIC17, TQ(UUG)E2, COG8, RTS2, ALG3, PRK1, TA(UGC)O, MRP51, AHA1, IES4, ASP1, CDC2, YPL191C, REC107, GTR2, DPB2, PCL7, VPS74, YHR113W, NRD1, SLX8, USE1, XBP1, PFK1, MSS2, PXA2, YNR029C, TH(GUG)K, CUP1-1, ALG5, FAR1, COX2, SLM6, TAF6, MSP1, YBR139W, AEP2, SKG6, NCS2, ACB1, VTC4, TIF6, YIL064W, HOM6, VMA21, GND1, SPC97, STR3, YDR341C, EMG1, SGF11, THR4, YBR014C, RIB3, ASK10, PIL1, RPL27A, TFB1, YFR055W, TOM71, RPA43, IRR1, TL(GAG)G, CAT8, SWR1, ARF3, HAP1, TMS1, NRG1, NIF3, ENT4, TRS120, AVT4, TCM62, DUT1,

				ALG1, YVC1, SOM1, NOC2, KOG1, STB3, TOM6, AAT1, NDE2, TS(UGA)E, HMF1, RPL8B, RPL18A, HHO1, SCH9, RET3, OAC1, SGA1, VHS1, HST4, OYE2, PFK27, RDS1, ECM29, CLB5, YCS4, YMR31, SDS24, RPN4, TRS130, MRL1, RPS16B, BAP3, RAD28, ARH1, TQ(UUG)L, MRPL50, RPS30B, ABD1, YML081W, MSH4, IRA2, JEN1, MUB1, NMD2, SLI15, YMR171C, NCA2, IST1, GAS2, RFA2, RPA14, SNO1, COX12, RGR1, HCH1, UBC9, URA6, SUA5, DTD1, SRL3, TOR1, RRP9, COX7, ADH2, LSB1, TSC10, SHM2, NUS1, DIM1, CAP2, SED1, HBT1, COS10, SET7, YDL124W, LRP1, SRN2, YOR246C, UTP20, GTO3, RAP1, NPY1, TPP1, ARG3, YFH1, BDH1, TE(UUC)K, ARN2, ARG80, MRM1, MRS1, ASF2, YPL109C, PCS60, FLO8, IPI3, THS1, LIP5, SRP72, FRS2
endoplasmic reticulum	173 out of 1943 genes, 8.9%	428 out of 6348 genes, 6.7%	0.00227	ERG2, ERG11, YPT1, ALG2, GET1, PMT1, DPL1, YCL045C, SSO2, ERV29, SWP1, ERP1, SEC39, YKE4, MNS1, YPT52, YDR307W, UBC6, ALG7, YPC1, STT3, MPD1, ATF2, EMP24, FLC1, ALG3, OPT1, TYW1, ERG9, PER1, SHR5, CPR5, HUT1, USE1, SUR4, ALG5, ERG5, SEC11, SEC16, LAG1, BIG1, LAS21, ERP3, VTC4, SLY41, VMA21, ERG7, NSG1, KRE6, CSG2, KAR2, ERD1, YNL194C, DFM1, SEC61, VMA22, YPR071W, ALG12, ALG14, SEC24, USA1, GPI16, SWF1, SED4, PKR1, PMT2,

				OST3, CWH41, PDI1, ERF2, ROT2, JEM1, ALG1, HOR7, EPS1, SAR1, MCD4, CDS1, HMX1, ERG3, DGA1, ALG6, MSC1, WBP1, NSG2, ERP2, YIF1, AQY2, MID1, GPI18, LEM3, RSB1, SEC17, NNF2, ERG28, DPM1, PHS1, BST1, RER2, SEC13, MNN9, SEC31, ERG6, RCE1, SEC12, YLR050C, GIP3, ERV2, ZRG17, APQ12, YOR285W, SWA2, GPI11, IRE1, CSM4, IZH1, YPR114W, SEC23, SCT1, YPR091C, YPR003C, ALG9, AVT2, PMT3, SFB3, BUD20, PGA3, YIP1, ERV41, YJL171C, HMG2, SEC66, ORM1, SWH1, PBN1, LHS1, SUR2, TSC13, LCB4, PMT5, MST28, SFB2, RPN1, LRO1, KRE5, TSC3, RTN1, YLL053C, TSC10, ILM1, SEC59, NUS1, YEL043W, PMT6, MST27, COS10, FPR2, SCP160, DER1, YLR004C, ERG25, LIP1, ERJ5, YPS1, GPI12, CWH43, GPI19, CNE1, GPI14, SRP72, GET3, NCP1, SAM3
cytoplasm	1333 out of 1943 genes, 68.6%	4102 out of 6348 genes, 64.6%	0.00232	SNX3, TR(UCU)E, TUF1, SPC19, DID2, URM1, BCH1, LOS1, IPP1, MUM2, YIR035C, SFI1, DYS1, AGX1, HIT1, QRI5, BSC2, YAR029W, DNF1, ABF2, CMP2, GCD10, MTO1, TAH1, MDM31, YDL119C, PUS4, CLU1, MNT2, RPL13B, CBP6, DMA1, ATF2, PET130, IRS4, YPT32, YSC83, TYW1, NTO1, TOS3, TK(CUU)J, MET14, TG(UCC)N, LYS1, TS(AGA)D2, CYM1, SEC14, YOR286W, CCP1, HUT1, NAT1, SEN15, YGR207C, YLR419W,

				ATO3, SSC1, HSP78, FAB1, TRM3, CYR1, ATG11, BUD13, CSG2, LSG1, ERD1, YPL236C, YTA6, GAL80, YPR071W, GCD7, CMK1, TE(UUC)J, ATP2, RIB4, GID7, RKI1, DOA4, SOL3, GPM3, CKS1, RNA1, CAN1, SUV3, YDR161W, HOM3, MSW1, LSP1, HOR7, TIF4632, YOL019W, PET309, TQ(UUG)D3, RRD1, CWC27, SAR1, CCL1, CAD1, YNL045W, ERP2, VHR1, GLO1, IMD2, SNF7, YIA6, MRP2, GPI18, SUP35, SWI6, HSP104, PCK1, AI5_BETA, HEM13, RIM20, RER2, TR(ACG)K, TG(GCC)B, PYK2, MDH2, SEC31, ERG6, FAA4, LST8, SGT2, CBR1, ARC15, ERV2, NDD1, TIM9, TOM70, PTC6, URE2, EHT1, GPI11, TI(AAU)L1, ARG8, YPR114W, TH(GUG)M, YHB1, IDP1, SNF4, INO1, MRPL3, CSL4, HAT2, ODC2, COT1, SEC66, YBR204C, GLC8, LHS1, UTR2, PMT5, PET10, RMI1, GOT1, TRP5, ILV5, LAG2, MRP20, NAB2, KTR7, DIN7, KAP104, RLI1, MST27, POM152, UBP16, POT1, KTR1, FPR2, MAK31, DUR1,2, CTR3, FMC1, SSK1, ERG25, ATP11, PIH1, HEM3, DIC1, YML018C, GPI19, ATG8, HUG1, ATG26, PRS4, SET5, IVY1, PUB1, ARO4, YAL061W, ISA1, RHO5, YGL039W, SMC6, TD(GUC)J1, SEC39, TG(CCC)D, NCA3, BUL1, EMP70, MYO1, BIO3, YLH47, RNH203, VPS38, ECM32, GOS1, ERG9, STF2, YMR291W, HCR1,

				HEK2, SHR5, RPS9B, ADE12, TS(AGA)B, YPR011C, LPP1, YPD1, PEX12, RPL9A, LAS21, PAN2, TA(UGC)L, SLY41, TR(UCU)B, LEU4, ERG7, KRE6, TQ(UUG)D1, MRPL44, RNR2, ECM21, FUS2, ACF2, ILV2, SER3, SSP120, HSL7, TS(AGA)A, PMT2, ERF2, RRI2, GRX3, PGI1, IMG2, RRN6, OSW2, PRO2, PDB1, CDS1, CTL1, YBP2, YGR043C, GCN1, ARO1, ERV15, TS(AGA)L, CLN2, IDH1, SKI3, SXM1, YHR020W, ADY2, SAP155, COX17, ERG28, MNP1, KEL3, TGL3, PRB1, SEC18, YJR149W, BST1, TE(CUC)D, MNN9, ADE5,7, CTM1, DSS1, SER1, GIP3, SSE2, GAD1, NAS6, ESP1, NIP1, GDA1, SSA2, RRI1, SHM1, BUD20, MAM33, TOM40, MIA40, VID24, MCH1, YAH1, ARD1, TSC13, LCB4, FMP46, HSE1, MDJ2, LRO1, KRE5, TT(AGU)J, CCT2, STF1, PDA1, YLL053C, BPH1, TM(CAU)J1, RPS0A, KAE1, TSA2, GIS4, SEC21, CYK3, RPL31B, SLX9, ERJ5, TL(UAA)J, ARG1, COX18, DMA2, URA1, CNE1, AFT1, GET3, NCP1, SNF1, JLP2, STE50, SAM3, PAI3, DOC1, SIS2, PDR5, MRP10, TRM10, PDE2, PMT1, ERV29, ILS1, LEU2, ERP1, YPR004C, ARP2, PCI8, USO1, MRPL33, BUD7, YPR127W, RPC82, TPC1, UTP13, PSK2, MNN2, OPT1, TOS4, ESC8, PEX22, HST2, YNL320W, COX15, MST1, CHS3, NOP14, KRE33, TQ(UUG)D2, STE18, PDR8, UBC13,

				PDS1, YGL157W, NEO1, SEC11, OSH6, NDC1, MSS18, ADO1, MHT1, COX9, RNR4, KAP122, HEF3, AFG1, KEX2, COR1, NSG1, YDL203C, TS(AGA)E, TOM20, SDH4, MUK1, FBP26, PRX1, ATP3, SEY1, DLD1, AVT7, CDC14, URA8, ENT1, DBP2, KAP120, YPL229W, ERG12, MGR1, ILV3, CWH41, PDI1, YMC2, CUP1-2, URA2, BCS1, COY1, GPD1, HEM1, GUT2, TYS1, LEU9, VAM6, COP1, DFG10, PUS6, MBF1, WBP1, MSF1, TE(UUC)L, GRE1, NSG2, YBR242W, GIP2, CAR2, YIF1, RRP1, CLC1, AQY2, SIW14, VPS20, PCL1, RSB1, NNF2, YCR023C, NAB6, AIR1, DRS2, ACO1, PPG1, ARO3, ZRG17, SNA2, GPH1, GLT1, FUN12, MAM3, TIF5, RPS30A, RNR3, MIH1, RAD50, SCT1, YPR091C, FMS1, YPL141C, PMT3, NUT1, YHL017W, ERV41, HMG2, MEC1, SPH1, PBN1, IKI1, SUR2, GUS1, SLT2, CSI1, DCS2, SSD1, CDC10, RPN1, SUP45, HFD1, TR(UCU)J1, HSM3, GAL83, DBP8, TC(GCA)P1, ICL2, NMA1, CAF130, PBS2, PPT1, MBA1, SCP160, ECM38, RAD26, KAR1, YPS1, CWH43, HSP60, SSA4, YGL080W, VTH1, SWI5, AAC3, TM(CAU)J3, ENA2, PHR1, REV7, MSE1, ALG2, RPL13A, GAL1, YGR130C, CLB1, DIA4, TIM21, YCL045C, PIB1, HEM12, ZRC1, AAH1, SIP1, ARG5,6, UBC6, GDI1, RPS24B, STT3, APA1,

				SFA1, DOA1, YMR031C, NDE1, PTC7, TQ(UUG)B, ARO7, LOT5, SDL1, TE(UUC)C, EMP24, PDR3, FAT1, PDH1, SYS1, YMR226C, HRR25, HSP82, CLB2, ATG19, ELP2, CPR5, SUR4, PRE5, SLH1, AAR2, TPO3, GAT1, CHD1, YPR172W, RPL16B, CSF1, RIM2, IDS2, SAP4, BIG1, SEC28, YOL048C, FKS1, ARG2, APS3, MRPS18, CSR2, RPL35A, GDH1, PNG1, VPS24, ALG12, RGD2, MEF1, TOM1, USA1, SAK1, RPO21, SED4, PCM1, CLN1, GIM3, YMR118C, PAN6, PHO12, YMR221C, YGL220W, PGM2, MCD4, FAA2, YPR174C, ADD37, ISU2, YDR061W, PHO8, RPL8A, CAK1, KRS1, MID1, ATG18, RPS0B, STV1, GLO2, SEN2, YKL071W, LEM3, SEC17, PET112, BUD21, COX1, THI4, AMD1, SEC13, HSP12, MBR1, TMA17, STP4, YLR050C, TRR2, MET28, ATG17, COQ3, APQ12, ATG1, SWA2, PSK1, TVP15, CCC2, ATG3, RPL6B, AIP1, YHC3, VBA1, BNR1, CDC39, YBL054W, TR(UCU)K, YIP1, ORM1, SVS1, VMA6, VAS1, HPT1, ISY1, GEA2, MSM1, TH(GUG)E1, YKT6, YNL134C, ADE8, TAD3, LYS2, COX23, LYS12, TN(GUU)K, PPZ1, PTP2, DOT6, YSC84, YGR111W, APC1, SKG3, UBX3, HOR2, YEA6, YJL045W, LSM3, OM45, YLR004C, CDC31, ATG7, TG(GCC)M, INM1, CCT4, TR(CCG)L, GLY1, GPI12, TP(AGG)C, STU1,

				KTR4, SUC2, GAL3, WRS1, HOF1, ERG2, YEL007W, CRN1, ATG2, YCF1, YLR126C, ERG11, CRC1, YPT1, RPG1, RPT3, RFS1, BNA4, GET1, RPL5, DPL1, YNL217W, MDY2, YKE4, SVF1, ECM31, MSD1, MKT1, MPA43, ENB1, TLG2, TS(AGA)J, DED1, ARN1, FLC1, YFR006W, MET31, TG(GCC)C, MRPL25, QCR8, SME1, COQ1, SMM1, IMP1, GLO4, TR(UCU)J2, SAM4, VIK1, SKI2, ERG5, UTR1, PAC10, YPL144W, SEC16, EFR3, DOG2, ERP3, TT(UGU)P, TG(UCC)O, YNR024W, UGA2, TPS3, SRL2, ATC1, ADE6, VIP1, KAR2, ATP8, TRM12, AVT1, SUT1, PEX11, APD1, YBR238C, SEC61, YIP5, UTP5, RPL9B, ALG14, ARG81, SEC24, UTP21, PRS2, AME1, NDI1, JEM1, BET4, VAM7, SPA2, RPS24A, MDL1, KAP123, MTQ1, CLB4, PEX10, ERG3, NMD5, YCR062W, ALG6, MSC1, MSB3, HOG1, RPL7B, SLS1, LSM4, CFT1, TOS1, RPL22B, CCA1, CWC23, RET2, EDC2, NRM1, ADH4, YOR285W, IRE1, IZH1, SPT7, KIP1, UPC2, AVT2, HNT1, LST4, RPT4, VRG4, MNN1, LEU1, MRPL20, TKL1, MRPL40, MST28, MMS2, CCE1, TSC3, TQ(UUG)E1, STE7, ZTA1, PPA2, PEP7, ILM1, TEL1, SER33, CDC36, PET122, ENT5, SEC27, WSC2, DER1, DBP5, NAS2, TUB3, PAC2, TR(UCU)M2, YLR247C, PIC2, NOC4, RRP14, PGS1, SCO1, EFT2, CHS6, TMA20, PRD1,

				TG(GCC)P2, TR(ACG)D, GPG1, MAK3, MRPL37, RPL35B, NOP1, YRO2, YGR250C, SNX41, PHO85, MNT3, YKR070W, TKL2, SCEI, CCT6, SWP1, PRP28, TE(UUC)P, ACO2, YDR307W, YKR096W, RRP5, VTA1, TS(AGA)M, MRPL27, YPC1, HAT1, BEM4, ODC1, YRM1, UBA1, YDR128W, PER1, KAP95, MSS116, TV(CAC)D, IDH2, SEC15, URA7, FPS1, MVB12, YRF1-7, YOR271C, RPS27B, TA(AGC)K2, GTO1, PHB2, TA(UGC)A, GCD6, LAG1, PEX13, CBC2, TAL1, DLD2, IMD4, ATG5, UTP15, UBP3, IDI1, STO1, MOD5, DFM1, VMA22, SIP5, GIP4, HPA3, MSS51, SHC1, MTG2, GCS1, TMA10, HAL9, BSP1, SUE1, ARO2, APN1, CYC7, ROT2, GRX5, YBR025C, SEN54, RGD1, RML2, HMX1, QCR9, DGA1, PMU1, SKI6, PMC1, YNL144C, VPS15, ALD2, PHS1, YLR412W, APL3, MDM20, GIS1, UBP14, VPS25, RCE1, GAP1, SEC12, VMA10, CPR3, YAP3, FDH1, SOD1, LPD1, HSP26, YFR011C, SEC23, MRPL39, VPS52, PBP1, OMS1, OSH3, MXR1, CIN5, TS(AGA)D3, OMA1, YDR520C, SWH1, ADE3, VPS73, YNK1, FET5, YNL274C, PTH1, PEA2, MET7, YNL168C, MAF1, YNL024C, SUI3, OPY2, BOI2, NBP1, NPR1, DUS3, ISM1, SBE22, RPT6, YKR075C, SEC59, LTE1, CSH1, PMT6, SLF1, FSH2, CTP1, MSS1, TMT1,

				YOR059C, GIS2, ICL1, PRS3, YIH1, VCX1, GCV2, LSB3, ACN9, RSM10, MTG1, ALD3, SUI1, PBI2, DAP2, PCL6, ADH3, MRPL8, HAS1, MTM1, PSE1, GCN20, AI4, GRE3, CAR1, PCL8, AUS1, RPS29A, MIS1, PIM1, MNS1, GIM4, YPT52, VAM3, ICY1, DBF20, LDB17, ALG7, MRH1, TYR1, TV(UAC)B, RNR1, PFK2, YPR022C, PTH2, RBG2, YLR345W, MOB1, DDI1, ARX1, MDM35, PSD2, DCS1, PRR1, RUB1, FMT1, RHR2, YOR262W, NPT1, YNL194C, TAZ1, PUF2, HYP2, TQ(UUG)C, TN(GUU)C, SGN1, SEC53, ADD66, GCD1, HSP10, GYP6, GPI16, SWF1, ECM18, YNL247W, IML1, RPB7, NTH1, UTP22, PKR1, OST3, COX20, YRR1, SOL4, ELP3, AAT2, PAN5, TG(GCC)J2, SHY1, TG(GCC)O2, EPS1, PKH1, PET111, CIN2, CDC50, MTF1, ATH1, YJL213W, TE(UUC)E1, PEP12, HAM1, UBC8, CCT5, ASN2, YPR118W, MSI1, LST7, DPM1, NIT3, SIZ1, AST2, SLC1, ALD4, TE(UUC)M, GSC2, LSC2, AAP1, NOG1, TR(UCU)D, NAM2, MDM30, CSM4, SMD3, SMF1, YAL049C, MGM1, RPL24B, TPO2, DAL7, TS(AGA)D1, YPR003C, ALG9, IMP2, SFB3, YOR283W, GUK1, PPH3, PGA3, YJL171C, FAS2, TUB1, TE(UUC)B, DPS1, MET10, TS(UGA)P, SFB2, APT1, VAC8, UMP1, RTN1, MAK10, ILV1, GNP1, DEG1, PFA3, MIG3, MET2,

				YEL043W, SLM5, ARP10, SIT1, ABP140, YDJ1, HOM2, CNS1, AXL2, LIP1, EFT1, TG(CCC)O, ESS1, HXK1, GPI14, ZWF1, PRT1, YNL176C, AI2, TR(UCU)M1, POS5, ECI1, YLR281C, HOL1, EHD3, HSP42, PRO1, SSO2, NAT2, CTF18, RRF1, KTR2, MPD1, MRPL11, MIC17, COG8, TQ(UUG)E2, RTS2, ALG3, PRK1, TA(UGC)O, MRP51, AHA1, ASP1, YPL191C, GTR2, DPB2, PCL7, VPS74, YHR113W, PFK1, MSS2, USE1, PXA2, YNR029C, TH(GUG)K, CUP1-1, ALG5, FAR1, COX2, MSP1, YBR139W, AEP2, SKG6, NCS2, ACB1, VTC4, TIF6, YIL064W, HOM6, VMA21, GND1, SPC97, STR3, YDR341C, EMG1, THR4, RIB3, YBR014C, ASK10, PIL1, RPL27A, YFR055W, TOM71, TL(GAG)G, ARF3, TMS1, NIF3, ENT4, TRS120, AVT4, TCM62, DUT1, ALG1, YVC1, SOM1, NOC2, KOG1, STB3, TOM6, AAT1, NDE2, TS(UGA)E, HMF1, RPL8B, RPL18A, SCH9, OAC1, RET3, SGA1, VHS1, HST4, OYE2, PFK27, ECM29, YMR31, SDS24, TRS130, MRL1, RPS16B, BAP3, ARH1, TQ(UUG)L, MRPL50, RPS30B, IRA2, JEN1, NMD2, YMR171C, NCA2, IST1, GAS2, SNO1, COX12, HCH1, URA6, SUA5, SRL3, DTD1, TOR1, RRP9, COX7, ADH2, LSB1, TSC10, SHM2, NUS1, DIM1, CAP2, SED1, COS10, HBT1, YDL124W, SRN2, YOR246C, UTP20, GTO3,

				NPY1, ARG3, YFH1, BDH1, ARN2, TE(UUC)K, MRM1, MRS1, PCS60, YPL109C, FLO8, THS1, LIP5, SRP72, FRS2
membrane-bounded organelle	1262 out of 1943 genes, 65.0%	3892 out of 6348 genes, 61.3%	0.01791	SNX3, SOH1, TUF1, SPC19, GCN4, DID2, URM1, BCH1, LOS1, AGX1, HIT1, QRI5, DNF1, STP3, RTT102, ABF2, GCD10, MTO1, TAH1, MDM31, YDL119C, PUS4, MNT2, CBP6, ATF2, STD1, PET130, IRS4, YPT32, YSC83, TYW1, NTO1, TK(CUU)J, CYM1, SEC14, YOR286W, CCP1, HUT1, NAT1, SEN15, YGR207C, YLR419W, ATO3, SSC1, HSP78, FAB1, CYR1, BUD13, NET1, CSG2, ERD1, YPL236C, BDF1, URB1, GAL80, YPR071W, GCD7, ATP2, RIB4, GRC3, SGS1, GID7, RKI1, DOA4, SOL3, CKS1, RNA1, CAN1, SUV3, YDR161W, MSW1, LSP1, HOR7, YOL019W, PET309, RRD1, CWC27, SAR1, CCL1, HDA2, CAD1, RET1, YNL045W, ERP2, VHR1, GLO1, SNF7, YIA6, MRP2, SNR51, GPI18, SPT20, SWI6, HSP104, AI5_BETA, HEM13, RIM20, RER2, PYK2, SEC31, ERG6, LST8, SGT2, CBR1, ARC15, TFB3, POP8, REF2, ERV2, NDD1, TIM9, TOM70, CET1, PTC6, EHT1, GPI11, DSN1, ARG8, ZAP1, YPR114W, YHB1, FOB1, IDP1, SNF4, STE12, MRPL3, CSL4, HAT2, ODC2, COT1, RPC37, SEC66, YBR204C, RAD16, GLC8, LHS1, PMT5, RMI1, GOT1, TRP5, ILV5, LAG2, MRP20, YTH1, NAB2, RLM1, SRB2, KTR7, DIN7, UBX6, RLI1, MAM1, CDC7,

				MSN5, PRP5, MST27, POM152, UBP16, POT1, SNR11, KTR1, FPR2, CTR3, FMC1, ERG25, ATP11, RFM1, PIH1, HEM3, DIC1, YML018C, GPI19, ATG8, HUG1, HTB1, SET5, YER130C, RSC9, IVY1, PUB1, SUT2, ARO4, YAL061W, ISA1, RHO5, RTT106, SWI4, SMC6, SEC39, NCA3, EMP70, SGF29, MOT3, YLH47, RNH203, PSY4, MTR2, VPS38, GOS1, ERG9, STF2, SMB1, YMR291W, HEK2, MFT1, SHR5, RPS9B, YPR011C, LPP1, YPD1, PEX12, LAS21, SLY41, LEU4, ERG7, SNT309, KRE6, YTM1, MRPL44, RNR2, FUS2, NOP16, HTZ1, ILV2, PMT2, ERF2, RRI2, GRX3, PGI1, SRM1, IMG2, RRN6, PRO2, YKU80, HPR5, EAF5, PDB1, SAC3, CDS1, CTL1, YBP2, SAD1, YGR043C, GCN1, TAF13, ERV15, CLN2, EAF7, IDH1, SKI3, SEN1, SXM1, GCR1, ADY2, SNR56, COX17, ERG28, MNP1, PRB1, BST1, NUP84, MNN9, DSS1, GIP3, YOL054W, ESP1, PRP42, YHC1, GDA1, BRF1, SSA2, RRI1, SHM1, BUD20, MAM33, BUR6, TOM40, MIA40, VID24, MCH1, YAH1, SNR6, TSC13, LCB4, MIF2, FMP46, HSE1, MDJ2, LRO1, TAF4, KRE5, NOP53, MAL23, STF1, PDA1, YLL053C, BPH1, ECM11, NUP85, YLR278C, KAE1, POM34, SEC21, PRP6, SLX9, NUT2, ERJ5, SPO12, COX18, CNE1, AFT1, GET3, NCP1, SNF1, SAM3, DOC1, TDP1, SIS2,

				SSU72, PDR5, MRP10, TRM10, PDE2, PMT1, ERV29, TRA1, ERP1, YPR004C, SLD5, ARP2, PCI8, USO1, MRPL33, BUD7, NRG2, RPB9, YPR127W, RPC82, TPC1, UTP13, PSK2, UGA3, MNN2, OPT1, TOS4, ESC8, PEX22, GAR1, HST2, YNL320W, COX15, MST1, CHS3, NOP14, KRE33, HOS1, PDR8, ORC5, UBC13, PDS1, YGL157W, NEO1, SEC11, NDC1, MSS18, ADO1, COX9, CTF13, HAC1, RNR4, KAP122, YRF1-6, AFG1, KEX2, COR1, NSG1, MSH5, YDL203C, TOM20, SDH4, PRX1, ATP3, DLD1, AVT7, ZIP1, CDC14, LOC1, DBP2, SDA1, KAP120, MED11, MGR1, ILV3, YER184C, CWH41, PDI1, PHD1, YMC2, SPT8, URA2, BCS1, COY1, POP6, GPD1, HEM1, GUT2, TYS1, LEU9, EST2, VAM6, COP1, PUS6, MBF1, WBP1, MSF1, NSG2, YBR242W, CAR2, YIF1, RRP1, CLC1, AQY2, YCL074W, VPS20, CDC5, PCL1, RSB1, NNF2, YCR023C, SNR61, AIR1, DRS2, ACO1, PPG1, ARO3, DRS1, ZRG17, DAL81, KRI1, GLT1, CUS2, FUN12, MAM3, MCM16, SRB8, RNR3, MIH1, RAD50, SCT1, YPR091C, PMT3, NUT1, IML3, YHL017W, ERV41, HMG2, MEC1, PBN1, IKI1, SUR2, SLT2, CSI1, RPN1, YRF1-1, YRB2, HFD1, CUP9, GAL83, DBP8, ICL2, NMA1, CAF130, HPR1, PPT1, MET18, MBA1, SCP160, ECM38, RAD26,

				YPS1, CWH43, HSP60, SSA4, DIB1, YGL080W, VTH1, SWI5, IES6, AAC3, GTT3, ENA2, PHR1, REV7, MSE1, ALG2, CLB1, DIA4, RDH54, TIM21, YCL045C, PIB1, ZRC1, HEM12, CAC2, AAH1, SIP1, PES4, ARG5,6, UBC6, APA1, STT3, SFA1, DOA1, YMR031C, NDE1, PTC7, ARO7, LOT5, EMP24, PDR3, FAT1, PDH1, SYS1, YMR226C, HRR25, CHL1, CLB2, SMX2, ELP2, CPR5, SUR4, PRE5, AAR2, TPO3, GAT1, CHD1, YPR172W, SEF1, CSF1, RIM2, IDS2, SAP4, BIG1, SEC28, POP3, FKS1, ARG2, APS3, MRPS18, CSR2, RAD3, GDH1, PNG1, VPS24, ALG12, MEF1, TOM1, USA1, RPO21, SED4, PCM1, CLN1, YMR118C, PAN6, YBR033W, PUS1, PHO12, YMR221C, YGL220W, GBP2, MCD4, PRP18, FAA2, YPR174C, ISU2, IXR1, HYS2, YDR061W, PHO8, RPA49, MID1, TFG1, ATG18, STV1, SEN2, LEM3, SEC17, GAT2, FPR3, PET112, BUD21, COX1, THI4, SEC13, HSP12, MBR1, TMA17, STP4, YLR050C, TRR2, MET28, COQ3, APQ12, SWA2, TVP15, SNR19, CCC2, AIP1, YHC3, VBA1, CDC39, YBL054W, YOX1, YIP1, RPC40, ORM1, SVS1, VMA6, VAS1, HPT1, ISY1, GEA2, MSM1, YKT6, YNL134C, ADE8, TAD3, COX23, SMX3, LYS12, PPZ1, PTP2, DOT6, CTK2, YGR111W, APC1, HOR2, YEA6, YJL045W, INO4, LSM3, OM45, YLR004C,

				CDC31, ATG7, INM1, MLP1, GPI12, TPA1, STU1, KTR4, SUC2, ORC4, ERG2, YEL007W, YCF1, ERG11, CRC1, IST3, YPT1, RPT3, TOS8, MSH2, BNA4, MAL33, GET1, DPL1, YNL217W, MDY2, YKE4, ECM31, SVF1, MSD1, TPK2, ENB1, TLG2, MPA43, ARN1, FLC1, FCF2, MET31, MRPL25, SDS3, QCR8, SME1, COQ1, TEL2, SMM1, PUS7, IMP1, GLO4, POL5, NHP10, SAM4, VIK1, SKI2, ERG5, UTR1, CDC55, SEC16, EFR3, DOG2, SSL1, ERP3, STB5, YNR024W, NNF1, SRL2, ATC1, KAR2, ATP8, AVT1, SUT1, PEX11, APD1, YBR238C, SEC61, YIP5, SSN2, UTP5, HMS1, ALG14, ARG81, SEC24, UTP21, SWD3, AME1, NDI1, YSF3, DAL80, JEM1, VAM7, RPS24A, MDL1, KAP123, MTQ1, CLB4, PEX10, ERG3, NMD5, ALG6, MSC1, RDS3, HOG1, SNF11, SLS1, RPA135, LSM4, CFT1, TOS1, YRF1-3, RGT1, CCA1, CWC23, RET2, EDC2, NRM1, ADH4, SIR1, YOR285W, IRE1, IZH1, CTI6, SPT7, UPC2, AVT2, HNT1, LST4, RPT4, VRG4, LSM5, MNN1, MRPL20, MRPL40, MST28, MMS2, DAD4, CCE1, TSC3, ZTA1, PPA2, YRF1-2, PEP7, ILM1, TEL1, RSC30, CDC36, PET122, ENT5, CLB6, SEC27, DER1, DBP5, TUB3, YLR247C, PIC2, NOC4, RRP14, PGS1, SCO1, BRR2, CHS6, PRD1, ULP1, MAK3, IOC4, FIP1, MRPL37, DAD2, RSC58, NOP1,

				NUP170, YRO2, SNX41, PHO85, NTG2, YKR070W, MNT3, TKL2, SCEI, SWP1, PRP28, ACO2, YDR307W, YKR096W, RRP5, SSL2, VTA1, MRPL27, YPC1, RPO31, HAT1, BEM4, ODC1, YRM1, UBA1, SRB7, YDR128W, PRI2, PER1, KAP95, RAD1, MSS116, IDH2, HMRA1, PRP8, YNR063W, FPS1, MVB12, YRF1-7, YOR271C, GTO1, NOP13, PHB2, SNM1, MND2, LAG1, PEX13, CBC2, TAF14, RNT1, DLD2, CRP1, ATG5, UTP15, STO1, MOD5, DFM1, VMA22, HPA3, MSS51, MTG2, ORC2, GCS1, TMA10, HAL9, SUE1, CDC21, APN1, CYC7, ROT2, GRX5, RRB1, TOP3, INO80, SPT21, SEN54, SPT3, RML2, HMX1, QCR9, PMU1, DGA1, HHT1, SKI6, PMC1, YNL144C, VPS15, TAF2, FYV6, PHS1, APL3, GIS1, UBP14, VPS25, RCE1, GAP1, SEC12, VMA10, CPR3, YAP3, SOD1, SPT4, PFS2, LPD1, HSP26, YFR011C, URB2, SEC23, MRPL39, VPS52, PBP1, OMS1, MXR1, CIN5, OMA1, YDR520C, SWH1, ADE3, VPS73, YNK1, FET5, YNL274C, PTH1, THP1, MET7, YNL168C, MAF1, NUP157, OPY2, SUA7, NBP1, NPR1, DUS3, ISM1, SBE22, RPT6, YKR075C, SEC59, CSH1, PMT6, SLF1, YLR108C, CTP1, MSS1, YIH1, SNU114, VCX1, WTM2, GCV2, LSB3, ACN9, RSM10, MTG1, ERB1, PBI2, IBD2, DAP2, PCL6,

				ADH3, MRPL8, HAS1, MTM1, PSE1, YCR087C-A, GAL4, AI4, GRE3, RPA190, PCL8, AUS1, MIS1, PIM1, MNS1, YPT52, GRX4, VAM3, YJL103C, ICY1, ALG7, MRH1, PFK2, HHT2, THP2, YPR022C, PTH2, MOB1, NUP145, ARX1, MDM35, PDS5, PSD2, DCS1, FMT1, RHR2, NPT1, MAG1, TAF10, YNL194C, TAZ1, RAD57, HYP2, CDC4, NPL6, LGE1, FPR4, HSP10, GYP6, GPI16, SWF1, ECM18, IML1, RPB7, PKR1, UTP22, FZF1, OST3, COX20, YRR1, SPT10, SOL4, AAT2, ELP3, SHY1, EPS1, PET111, CDC50, MTF1, ATH1, PEP12, HAM1, SSF2, NUP192, YPR118W, DLS1, MSI1, LST7, YLL054C, RPI1, CDC6, DPM1, NIT3, SIZ1, RAD59, ALD4, DAL82, SNR58, LSC2, AAP1, TRF5, NOG1, NAM2, RPF1, MDM30, CSM4, RAD34, SMD3, SMF1, YTA7, MGM1, TPO2, BRR1, DAL7, YPR003C, ALG9, MED2, IMP2, SFB3, YOR283W, GUK1, PPH3, PGA3, YJL171C, FAS2, TUB1, SFB2, APT1, VAC8, UMP1, SWI1, RTN1, ILV1, GNP1, DEG1, PFA3, MIG3, RME1, YEL043W, SLM5, AZF1, SIT1, ASH1, ACA1, CDC26, HOM2, LIP1, RFC3, ESS1, GPI14, HAP2, YNL176C, NUP100, AI2, POS5, ECI1, YLR281C, HOL1, EHD3, ESF1, SWD1, SSO2, STB2, NAT2, CTF18, RRF1, KTR2, CSE4, MPD1, MRPL11, MIC17, COG8, RTS2, ALG3, MRP51, IES4, CDC2,

				REC107, GTR2, DPB2, PCL7, VPS74, YHR113W, NRD1, XBP1, PFK1, MSS2, USE1, PXA2, ALG5, FAR1, COX2, SLM6, TAF6, MSP1, YBR139W, AEP2, ACB1, VTC4, TIF6, HOM6, VMA21, STR3, GND1, SPC97, YDR341C, EMG1, SGF11, THR4, RIB3, YBR014C, ASK10, PIL1, TFB1, RPA43, TOM71, IRR1, CAT8, SWR1, ARF3, HAP1, TMS1, NIF3, NRG1, TRS120, AVT4, TCM62, DUT1, ALG1, YVC1, SOM1, KOG1, NOC2, AAT1, TOM6, NDE2, HMF1, HHO1, SCH9, OAC1, RET3, SGA1, HST4, OYE2, RDS1, ECM29, CLB5, YCS4, YMR31, RPN4, TRS130, MRL1, BAP3, RAD28, ARH1, MRPL50, ABD1, YML081W, MSH4, IRA2, JEN1, MUB1, SLI15, YMR171C, NCA2, IST1, RPA14, RFA2, COX12, RGR1, HCH1, URA6, UBC9, TOR1, RRP9, COX7, LSB1, TSC10, NUS1, DIM1, SED1, SET7, COS10, YDL124W, SRN2, LRP1, UTP20, RAP1, NPY1, TPP1, YFH1, ARN2, MRS1, MRM1, ARG80, ASF2, PCS60, YPL109C, FLO8, IPI3, THS1, LIP5, SRP72
intracellular membrane-bounded organelle	1262 out of 1943 genes, 65.0%	3892 out of 6348 genes, 61.3%	0.01791	SNX3, SOH1, TUF1, SPC19, GCN4, DID2, URM1, BCH1, LOS1, AGX1, HIT1, QRI5, DNF1, STP3, RTT102, ABF2, GCD10, MTO1, TAH1, MDM31, YDL119C, PUS4, MNT2, CBP6, ATF2, STD1, PET130, IRS4, YPT32, YSC83, TYW1, NTO1, TK(CUU)J, CYM1, SEC14, YOR286W, CCP1,

				HUT1, NAT1, SEN15, YGR207C, YLR419W, ATO3, SSC1, HSP78, FAB1, CYR1, BUD13, NET1, CSG2, ERD1, YPL236C, BDF1, URB1, GAL80, YPR071W, GCD7, ATP2, RIB4, GRC3, SGS1, GID7, RKI1, DOA4, SOL3, CKS1, RNA1, CAN1, SUV3, YDR161W, MSW1, LSP1, HOR7, YOL019W, PET309, RRD1, CWC27, SAR1, CCL1, HDA2, CAD1, RET1, YNL045W, ERP2, VHR1, GLO1, SNF7, YIA6, MRP2, SNR51, GPI18, SPT20, SWI6, HSP104, AI5_BETA, HEM13, RIM20, RER2, PYK2, SEC31, ERG6, LST8, SGT2, CBR1, ARC15, TFB3, POP8, REF2, ERV2, NDD1, TIM9, TOM70, CET1, PTC6, EHT1, GPI11, DSN1, ARG8, ZAP1, YPR114W, YHB1, FOB1, IDP1, SNF4, STE12, MRPL3, CSL4, HAT2, ODC2, COT1, RPC37, SEC66, YBR204C, RAD16, GLC8, LHS1, PMT5, RMI1, GOT1, TRP5, ILV5, LAG2, MRP20, YTH1, NAB2, RLM1, SRB2, KTR7, DIN7, UBX6, RLI1, MAM1, CDC7, MSN5, PRP5, MST27, POM152, UBP16, POT1, SNR11, KTR1, FPR2, CTR3, FMC1, ERG25, ATP11, RFM1, PIH1, HEM3, DIC1, YML018C, GPI19, ATG8, HUG1, HTB1, SET5, YER130C, RSC9, IVY1, PUB1, SUT2, ARO4, YAL061W, ISA1, RHO5, RTT106, SWI4, SMC6, SEC39, NCA3, EMP70, SGF29, MOT3, YLH47, RNH203, PSY4, MTR2, VPS38, GOS1, ERG9, STF2, SMB1, YMR291W, HEK2,

				MFT1, SHR5, RPS9B, YPR011C, LPP1, YPD1, PEX12, LAS21, SLY41, LEU4, ERG7, SNT309, KRE6, YTM1, MRPL44, RNR2, FUS2, NOP16, HTZ1, ILV2, PMT2, ERF2, RRI2, GRX3, PGI1, SRM1, IMG2, RRN6, PRO2, YKU80, HPR5, EAF5, PDB1, SAC3, CDS1, CTL1, YBP2, SAD1, YGR043C, GCN1, TAF13, ERV15, CLN2, EAF7, IDH1, SKI3, SEN1, SXM1, GCR1, ADY2, SNR56, COX17, ERG28, MNP1, PRB1, BST1, NUP84, MNN9, DSS1, GIP3, YOL054W, ESP1, PRP42, YHC1, GDA1, BRF1, SSA2, RRI1, SHM1, BUD20, MAM33, BUR6, TOM40, MIA40, VID24, MCH1, YAH1, SNR6, TSC13, LCB4, MIF2, FMP46, HSE1, MDJ2, LRO1, TAF4, KRE5, NOP53, MAL23, STF1, PDA1, YLL053C, BPH1, ECM11, NUP85, YLR278C, KAE1, POM34, SEC21, PRP6, SLX9, NUT2, ERJ5, SPO12, COX18, CNE1, AFT1, GET3, NCP1, SNF1, SAM3, DOC1, TDP1, SIS2, SSU72, PDR5, MRP10, TRM10, PDE2, PMT1, ERV29, TRA1, ERP1, YPR004C, SLD5, ARP2, PCI8, USO1, MRPL33, BUD7, NRG2, RPB9, YPR127W, RPC82, TPC1, UTP13, PSK2, UGA3, MNN2, OPT1, TOS4, ESC8, PEX22, GAR1, HST2, YNL320W, COX15, MST1, CHS3, NOP14, KRE33, HOS1, PDR8, ORC5, UBC13, PDS1, YGL157W, NEO1, SEC11, NDC1, MSS18, ADO1, COX9,

				CTF13, HAC1, RNR4, KAP122, YRF1-6, AFG1, KEX2, COR1, NSG1, MSH5, YDL203C, TOM20, SDH4, PRX1, ATP3, DLD1, AVT7, ZIP1, CDC14, LOC1, DBP2, SDA1, KAP120, MED11, MGR1, ILV3, YER184C, CWH41, PDI1, PHD1, YMC2, SPT8, URA2, BCS1, COY1, POP6, GPD1, HEM1, GUT2, TYS1, LEU9, EST2, VAM6, COP1, PUS6, MBF1, WBP1, MSF1, NSG2, YBR242W, CAR2, YIF1, RRP1, CLC1, AQY2, YCL074W, VPS20, CDC5, PCL1, RSB1, NNF2, YCR023C, SNR61, AIR1, DRS2, ACO1, PPG1, ARO3, DRS1, ZRG17, DAL81, KRI1, GLT1, CUS2, FUN12, MAM3, MCM16, SRB8, RNR3, MIH1, RAD50, SCT1, YPR091C, PMT3, NUT1, IML3, YHL017W, ERV41, HMG2, MEC1, PBN1, IKI1, SUR2, SLT2, CSI1, RPN1, YRF1-1, YRB2, HFD1, CUP9, GAL83, DBP8, ICL2, NMA1, CAF130, HPR1, PPT1, MET18, MBA1, SCP160, ECM38, RAD26, YPS1, CWH43, HSP60, SSA4, DIB1, YGL080W, VTH1, SWI5, IES6, AAC3, GTT3, ENA2, PHR1, REV7, MSE1, ALG2, CLB1, DIA4, RDH54, TIM21, YCL045C, PIB1, ZRC1, HEM12, CAC2, AAH1, SIP1, PES4, ARG5,6, UBC6, APA1, STT3, SFA1, DOA1, YMR031C, NDE1, PTC7, ARO7, LOT5, EMP24, PDR3, FAT1, PDH1, SYS1, YMR226C, HRR25, CHL1, CLB2, SMX2, ELP2, CPR5, SUR4, PRE5, AAR2, TPO3, GAT1,

				CHD1, YPR172W, SEF1, CSF1, RIM2, IDS2, SAP4, BIG1, SEC28, POP3, FKS1, ARG2, APS3, MRPS18, CSR2, RAD3, GDH1, PNG1, VPS24, ALG12, MEF1, TOM1, USA1, RPO21, SED4, PCM1, CLN1, YMR118C, PAN6, YBR033W, PUS1, PHO12, YMR221C, YGL220W, GBP2, MCD4, PRP18, FAA2, YPR174C, ISU2, IXR1, HYS2, YDR061W, PHO8, RPA49, MID1, TFG1, ATG18, STV1, SEN2, LEM3, SEC17, GAT2, FPR3, PET112, BUD21, COX1, THI4, SEC13, HSP12, MBR1, TMA17, STP4, YLR050C, TRR2, MET28, COQ3, APQ12, SWA2, TVP15, SNR19, CCC2, AIP1, YHC3, VBA1, CDC39, YBL054W, YOX1, YIP1, RPC40, ORM1, SVS1, VMA6, VAS1, HPT1, ISY1, GEA2, MSM1, YKT6, YNL134C, ADE8, TAD3, COX23, SMX3, LYS12, PPZ1, PTP2, DOT6, CTK2, YGR111W, APC1, HOR2, YEA6, YJL045W, INO4, LSM3, OM45, YLR004C, CDC31, ATG7, INM1, MLP1, GPI12, TPA1, STU1, KTR4, SUC2, ORC4, ERG2, YEL007W, YCF1, ERG11, CRC1, IST3, YPT1, RPT3, TOS8, MSH2, BNA4, MAL33, GET1, DPL1, YNL217W, MDY2, YKE4, ECM31, SVF1, MSD1, TPK2, ENB1, TLG2, MPA43, ARN1, FLC1, FCF2, MET31, MRPL25, SDS3, QCR8, SME1, COQ1, TEL2, SMM1, PUS7, IMP1, GLO4, POL5, NHP10, SAM4, VIK1, SKI2, ERG5,

				UTR1, CDC55, SEC16, EFR3, DOG2, SSL1, ERP3, STB5, YNR024W, NNF1, SRL2, ATC1, KAR2, ATP8, AVT1, SUT1, PEX11, APD1, YBR238C, SEC61, YIP5, SSN2, UTP5, HMS1, ALG14, ARG81, SEC24, UTP21, SWD3, AME1, NDI1, YSF3, DAL80, JEM1, VAM7, RPS24A, MDL1, KAP123, MTQ1, CLB4, PEX10, ERG3, NMD5, ALG6, MSC1, RDS3, HOG1, SNF11, SLS1, RPA135, LSM4, CFT1, TOS1, YRF1-3, RGT1, CCA1, CWC23, RET2, EDC2, NRM1, ADH4, SIR1, YOR285W, IRE1, IZH1, CTI6, SPT7, UPC2, AVT2, HNT1, LST4, RPT4, VRG4, LSM5, MNN1, MRPL20, MRPL40, MST28, MMS2, DAD4, CCE1, TSC3, ZTA1, PPA2, YRF1-2, PEP7, ILM1, TEL1, RSC30, CDC36, PET122, ENT5, CLB6, SEC27, DER1, DBP5, TUB3, YLR247C, PIC2, NOC4, RRP14, PGS1, SCO1, BRR2, CHS6, PRD1, ULP1, MAK3, IOC4, FIP1, MRPL37, DAD2, RSC58, NOP1, NUP170, YRO2, SNX41, PHO85, NTG2, YKR070W, MNT3, TKL2, SCEI, SWP1, PRP28, ACO2, YDR307W, YKR096W, RRP5, SSL2, VTA1, MRPL27, YPC1, RPO31, HAT1, BEM4, ODC1, YRM1, UBA1, SRB7, YDR128W, PRI2, PER1, KAP95, RAD1, MSS116, IDH2, HMRA1, PRP8, YNR063W, FPS1, MVB12, YRF1-7, YOR271C, GTO1, NOP13, PHB2, SNM1, MND2, LAG1, PEX13, CBC2,

				TAF14, RNT1, DLD2, CRP1, ATG5, UTP15, STO1, MOD5, DFM1, VMA22, HPA3, MSS51, MTG2, ORC2, GCS1, TMA10, HAL9, SUE1, CDC21, APN1, CYC7, ROT2, GRX5, RRB1, TOP3, INO80, SPT21, SEN54, SPT3, RML2, HMX1, QCR9, PMU1, DGA1, HHT1, SKI6, PMC1, YNL144C, VPS15, TAF2, FYV6, PHS1, APL3, GIS1, UBP14, VPS25, RCE1, GAP1, SEC12, VMA10, CPR3, YAP3, SOD1, SPT4, PFS2, LPD1, HSP26, YFR011C, URB2, SEC23, MRPL39, VPS52, PBP1, OMS1, MXR1, CIN5, OMA1, YDR520C, SWH1, ADE3, VPS73, YNK1, FET5, YNL274C, PTH1, THP1, MET7, YNL168C, MAF1, NUP157, OPY2, SUA7, NBP1, NPR1, DUS3, ISM1, SBE22, RPT6, YKR075C, SEC59, CSH1, PMT6, SLF1, YLR108C, CTP1, MSS1, YIH1, SNU114, VCX1, WTM2, GCV2, LSB3, ACN9, RSM10, MTG1, ERB1, PBI2, IBD2, DAP2, PCL6, ADH3, MRPL8, HAS1, MTM1, PSE1, YCR087C-A, GAL4, AI4, GRE3, RPA190, PCL8, AUS1, MIS1, PIM1, MNS1, YPT52, GRX4, VAM3, YJL103C, ICY1, ALG7, MRH1, PFK2, HHT2, THP2, YPR022C, PTH2, MOB1, NUP145, ARX1, MDM35, PDS5, PSD2, DCS1, FMT1, RHR2, NPT1, MAG1, TAF10, YNL194C, TAZ1, RAD57, HYP2, CDC4, NPL6, LGE1, FPR4, HSP10, GYP6, GPI16, SWF1, ECM18, IML1,

				RPB7, PKR1, UTP22, FZF1, OST3, COX20, YRR1, SPT10, SOL4, AAT2, ELP3, SHY1, EPS1, PET111, CDC50, MTF1, ATH1, PEP12, HAM1, SSF2, NUP192, YPR118W, DLS1, MSI1, LST7, YLL054C, RPI1, CDC6, DPM1, NIT3, SIZ1, RAD59, ALD4, DAL82, SNR58, LSC2, AAP1, TRF5, NOG1, NAM2, RPF1, MDM30, CSM4, RAD34, SMD3, SMF1, YTA7, MGM1, TPO2, BRR1, DAL7, YPR003C, ALG9, MED2, IMP2, SFB3, YOR283W, GUK1, PPH3, PGA3, YJL171C, FAS2, TUB1, SFB2, APT1, VAC8, UMP1, SWI1, RTN1, ILV1, GNP1, DEG1, PFA3, MIG3, RME1, YEL043W, SLM5, AZF1, SIT1, ASH1, ACA1, CDC26, HOM2, LIP1, RFC3, ESS1, GPI14, HAP2, YNL176C, NUP100, AI2, POS5, ECI1, YLR281C, HOL1, EHD3, ESF1, SWD1, SSO2, STB2, NAT2, CTF18, RRF1, KTR2, CSE4, MPD1, MRPL11, MIC17, COG8, RTS2, ALG3, MRP51, IES4, CDC2, REC107, GTR2, DPB2, PCL7, VPS74, YHR113W, NRD1, XBP1, PFK1, MSS2, USE1, PXA2, ALG5, FAR1, COX2, SLM6, TAF6, MSP1, YBR139W, AEP2, ACB1, VTC4, TIF6, HOM6, VMA21, STR3, GND1, SPC97, YDR341C, EMG1, SGF11, THR4, RIB3, YBR014C, ASK10, PIL1, TFB1, RPA43, TOM71, IRR1, CAT8, SWR1, ARF3, HAP1, TMS1, NIF3, NRG1, TRS120, AVT4, TCM62, DUT1, ALG1, YVC1,

				SOM1, KOG1, NOC2, AAT1, TOM6, NDE2, HMF1, HHO1, SCH9, OAC1, RET3, SGA1, HST4, OYE2, RDS1, ECM29, CLB5, YCS4, YMR31, RPN4, TRS130, MRL1, BAP3, RAD28, ARH1, MRPL50, ABD1, YML081W, MSH4, IRA2, JEN1, MUB1, SLI15, YMR171C, NCA2, IST1, RPA14, RFA2, COX12, RGR1, HCH1, URA6, UBC9, TOR1, RRP9, COX7, LSB1, TSC10, NUS1, DIM1, SED1, SET7, COS10, YDL124W, SRN2, LRP1, UTP20, RAP1, NPY1, TPP1, YFH1, ARN2, MRS1, MRM1, ARG80, ASF2, PCS60, YPL109C, FLO8, IPI3, THS1, LIP5, SRP72
membrane part	519 out of 1943 genes, 26.7%	1494 out of 6348 genes, 23.5%	0.02111	ERG2, ATG2, CRC1, ERG11, YOR223W, YCF1, YPT1, CRH1, GET1, AST1, DFG5, QRI5, BSC2, DNF1, YKE4, AGA1, MDM31, YDL119C, MTL1, TLG2, ENB1, MNT2, ATF2, ARN1, FLC1, YFR006W, SNF3, QCR8, CWP1, ITR2, IMP1, HUT1, SEN15, ATO3, SSC1, SEC16, ERP3, ATG11, CSG2, ATP8, AVT1, ERD1, YBR238C, YIP5, SEC61, YPR071W, ALG14, ATP2, SEC24, MUP1, CAN1, JEM1, DAL5, MDL1, KAP123, YOL019W, ENA5, PHO84, PET309, SAR1, ERG3, YCR062W, NMD5, ALG6, ERP2, SLS1, ENA1, SNF7, YIA6, GPI18, RET2, AI5_BETA, RER2, SEC31, LST8, CBR1, ARC15, ERV2, TOM70, GPI11, IRE1, IZH1, YPR114W, FIT1, PIN2, AVT2, LST4, ODC2, COT1, FLO1,

				SEC66, FRE3, VRG4, UTR2, MNN1, PMT5, GOT1, MST28, RGT2, BAP2, TSC3, MEP2, AXL1, KTR7, FUI1, HXT13, ILM1, PEP7, PRM2, MST27, POM152, ENT5, HXT2, SEC27, KTR1, FPR2, WSC2, CTR3, DER1, DBP5, FEN2, YLR046C, ERG25, PIC2, DIC1, SCO1, YML018C, GPI19, ATG8, ULP1, KTR3, NUP170, YRO2, NFT1, SSU1, MNT3, VTH2, SWP1, SEC39, EMP70, YDR307W, YLH47, YPC1, MNN4, DAN1, MTR2, IPT1, ODC1, EXG2, GOS1, YBR063C, ERG9, STF2, CDC1, PER1, KAP95, AGP1, SHR5, FPS1, MVB12, YOR271C, YPR011C, LPP1, PHB2, PEX12, PEX13, LAG1, LAS21, SLY41, ERG7, YHL044W, KRE6, DFM1, HXT16, VMA22, MSS51, FLO5, MTG2, TIR4, PMT2, STE4, ERF2, CYC7, FRE8, YIL171W, YBR284W, SEN54, SAC3, RAX1, TAT1, CDS1, QCR9, HMX1, DGA1, PMC1, FCY22, AZR1, ERV15, ECM7, SXM1, ADY2, ERG28, PHS1, TGL3, SEC18, APL3, BST1, NUP84, MNN9, VPS25, RCE1, GAP1, SEC12, VMA10, PRM7, DTR1, QDR2, GDA1, SEC23, DAL4, HKR1, PHO89, OMS1, GAS4, TOM40, MIA40, OMA1, SEC9, VID24, VPS73, MCH1, TSC13, FET5, LCB4, THP1, KRE1, MDJ2, LRO1, NUP157, OPY2, MCH5, STF1, YLL053C, SEC59, NUP85, CSH1, POM34, PMT6, CTP1, SEC21, YOR059C, ERJ5,

				VCX1, BUD8, COX18, URA1, CNE1, NCP1, SAM3, DAP2, MTM1, PDR5, TIR1, ITR1, AI4, PMT1, YPS6, ERP1, AUS1, ERV29, MNS1, VAM3, ALG7, MRH1, TPC1, DSE2, MNN2, OPT1, PEX22, YPS3, COX15, YNL320W, CHS3, NUP145, YPR117W, STE18, TAT2, SEC11, NEO1, NDC1, COX9, KAP122, COR1, KEX2, TRE1, NSG1, TOM20, SDH4, ATP3, YNL194C, SEY1, TAZ1, AVT7, YOR071C, HXT9, KAP120, YFL067W, HUR1, GPI16, SWF1, MGR1, YPR157W, PKR1, OST3, COX20, MID2, CWH41, YMC2, URA2, BCS1, COY1, GUT2, SHY1, VAM6, EPS1, COP1, DFG10, CDC50, WBP1, NSG2, PEP12, YIF1, AQY2, CLC1, NUP192, LST7, VPS20, RSB1, NNF2, YCR023C, DPM1, SLC1, GSC2, DRS2, PTR2, ZRG17, SNA2, SUL1, CSM4, MAM3, SMF1, MGM1, TPO2, SCT1, YPR091C, ALG9, YOR390W, YPR003C, FLO9, PMT3, IMP2, SFB3, YOR1, YHL017W, PGA3, YJL171C, HMG2, ERV41, PBN1, YOR008C-A, SUR2, SFB2, YRB2, RTN1, HFD1, BFR1, GNP1, PFA3, HXT12, SIT1, SCP160, ECM38, FCY2, AXL2, LIP1, YPS1, GAL2, YDR338C, ECM27, CWH43, BRE4, VTH1, AAC3, GPI14, MKC7, GTT3, YBR220C, ENA2, NUP100, YNL176C, MUC1, AI2, ALG2, HOL1, SFK1, YCL045C, FRE2, SSO2, TIM21, ZRC1, KTR2,

				HXT5, UBC6, STT3, EMP24, HXT11, NHA1, ALG3, FAT1, SYS1, SUR4, MSS2, USE1, PXA2, TPO3, DNF2, ALG5, COX2, CSF1, RIM2, MSP1, SKG6, SEC28, BIG1, FKS1, YOL048C, VTC4, APS3, PMP2, VMA21, ALP1, VPS24, ALG12, TOM71, SGE1, USA1, TMS1, SED4, AVT4, TCM62, ALG1, YMR118C, YVC1, SOM1, TOM6, YMR221C, MCD4, PHO8, OAC1, RET3, YNR066C, MID1, STV1, SEN2, LEM3, PRM5, YOL163W, COX1, SEC13, MRL1, BAP3, COS9, YLR050C, COQ3, APQ12, PCA1, SWA2, TVP15, CCC2, VHT1, VBA1, YHC3, JEN1, HXT15, YIP1, ORM1, FRE4, YMR171C, NCA2, VMA6, GAS2, COX12, YOR378W, GEA2, YIL166C, YKT6, TOR1, COX7, PPZ1, FUR4, TSC10, NUS1, SNG1, SED1, COS10, YEA6, SRN2, YAL065C, YOR246C, OM45, CDC31, YLR004C, MLP1, ARN2, GPI12, KTR4, YFL054C, MAL11, KTR6
mitochondrial part	204 out of 1943 genes, 10.5%	535 out of 6348 genes, 8.4%	0.02799	MRPL37, ADH3, MRPL8, MTM1, CRC1, TUF1, MSE1, POS5, BNA4, MRP10, ISA1, DIA4, QRI5, TIM21, PIM1, YPR004C, ABF2, MRPL33, ZRC1, MDM31, MSD1, ARG5,6, YDL119C, YLH47, MRPL27, MRPL11, TPC1, MIC17, NDE1, PDH1, PET130, MRP51, YPT32, YSC83, ODC1, MRPL25, QCR8, ERG9, STF2, COX15, MST1, MSS116, PTH2, COQ1, CYM1, IMP1, IDH2, CCP1, MDM35, GLO4, MSS2, SEN15,

				YGR207C, COX2, YOR271C, YPR011C, SSC1, PHB2, RIM2, HSP78, COX9, MSP1, ARG2, DLD2, MRPS18, AFG1, COR1, ATP8, TOM20, SDH4, ATP3, TAZ1, MRPL44, DLD1, RIB3, YBR238C, PIL1, TOM71, ATP2, MSS51, MTG2, HSP10, SUE1, MGR1, COX20, NDI1, SUV3, TCM62, CYC7, IMG2, YMC2, YMR118C, GRX5, SOM1, BCS1, MSW1, LSP1, MDL1, HEM1, GUT2, SHY1, SEN54, AAT1, TOM6, NDE2, PDB1, PET309, PET111, RML2, CDS1, QCR9, ISU2, MTF1, MSF1, OAC1, SLS1, YIA6, IDH1, MRP2, SEN2, CCA1, COX17, DPM1, MNP1, HEM13, YMR31, COX1, ALD4, ERG6, CBR1, ACO1, DSS1, ARC15, NAM2, COQ3, CPR3, TIM9, TOM70, PTC6, YOR285W, EHT1, SOD1, ARH1, ARG8, LPD1, MGM1, MRPL50, YHB1, MRPL39, IDP1, OMS1, IMP2, MRPL3, MAM33, TOM40, MIA40, ODC2, COT1, HMG2, OMA1, VPS73, YNK1, NCA2, YAH1, MRPL20, MRPL40, COX12, ILV5, MSM1, MRP20, MDJ2, CCE1, HFD1, ISM1, COX23, GNP1, COX7, STF1, PDA1, TSC10, ICL2, PET122, SLM5, UBP16, CTP1, YEA6, MSS1, MBA1, YJL045W, OM45, YFH1, ATP11, PIC2, MRS1, DIC1, ACN9, SCO1, RSM10, HSP60, COX18, MTG1, PRD1, AAC3, NCP1
