# Supplementary material for: Performing statistical analyses on quantitative data in Taverna workflows: An example using R and maxdBrowse to identify differentially-expressed genes from microarray data
Source: BMC Bioinformatics. 2008 Aug 7;9:334. doi: 10.1186/1471-2105-9-334 (PMC2528018; doi:10.1186/1471-2105-9-334)
Supplement: Additional file 3 — Nitrogen t-test. [file 1471-2105-9-334-S3.zip › 0.05ttest/0.05Go/molfunc.pdf]

Result Table

Terms from the Function Ontology with p-value as good or better than 0.05				
**Gene Ontology term**	**Cluster frequency**	**Genome frequency of use**	**Corrected P-value**	**Genes annotated to the term**
unannotated	24 out of 1943 genes, 1.2%	2 out of 6348 genes, 0.0%	0	YDL228C, ARS605, YFL015C, YKL033W-A_ALT, Q0270, YKR106W_1, YFL031W_EX2_ALT, PSY1, 2MIC_REP2, Q0155, TLC1_0, OPI6, YNL203C, Q0320, YRF, BUD30, MBB1, CEN13, YLR426W_EX2, Q0167, SNR17A_EX2, OPI8, TY1B_A_LR4, YEL074W
catalytic activity	860 out of 1943 genes, 44.3%	2371 out of 6348 genes, 37.4%	2.37e-11	ERG2, WRS1, ERG11, YLR126C, EEB1, YCF1, TUF1, RPT3, YPT1, IST3, RFS1, CRH1, BNA4, MSH2, IPP1, YIR035C, AST1, DFG5, DYS1, DPL1, AGX1, YNL217W, DNF1, CMP2, GCD10, ECM31, MSD1, TPK2, MKT1, PUS4, TGL2, MNT2, DED1, DMA1, ATF2, YFR006W, YPT32, TYW1, SDS3, QCR8, NTO1, TOS3, MET14, SME1, LYS1, COQ1, SMM1, CYM1, PUS7, CDC42, IMP1, YOR286W, CCP1, YNR071C, POL5, GLO4, NAT1, SEN15, SAM4, CDA1, YGR207C, YLR419W, ERG5, SKI2, SSC1, UTR1, CDC55, HSP78, FAB1, HIS3, DOG2, TRM3, CYR1, TPS3, UGA2, ADE6, VIP1, TRM12, ATP8, KAR2, LSG1, YPL236C, YTA6, GAL80, CMK1, HMS1, ALG14, ATP2, RIB4, PRS2, SWD3, SGS1, RKI1, DOA4, SOL3, GPM3, NDI1, SUV3, HOM3, BET4, BGL2, MSW1, MDL1, YIL165C, ENA5, MTQ1, RRD1, CWC27, PHO11, SAR1, GSH2, HDA2, ERG3, ALG6, AAD6, RET1, HOG1, YNL045W, RPA135, ENA1, LSM4, GLO1, IMD2, GLG1, YRF1-3, GPI18, CCA1, SUP35, RAS2, YJR142W, HSP104, PCK1, AI5_BETA, HEM13, RER2, PYK2, MDH2, ERG6, FAA4, CBR1, POP8, ADH4, ERV2, CET1, PTC6, EHT1, YOR285W, GPI11, IRE1, FSP2, ARG8, CTI6, YHB1, IDP1, SNF4, INO1, TEP1, MRPL3, CSL4, HNT1, HAT2, FAA3, RPC37, RPT4, YBR204C, FRE3, RAD16, LSM5, UTR2, MNN1, LEU1, PMT5, TKL1, TRP5, ILV5, MMS2, CCE1, NIT1, AXL1, KTR7, DIN7, PPA2, STE7, ZTA1, YRF1-2, TEL1, RSC30, SER33, RLI1, CDC36, CDC7, YFR007W, PRP5, UBP16, POT1, KTR1, FPR2, MAK31, DUR1,2, DBP5, BDS1, TUB3, YLR247C, ERG25, PGS1, HEM3, SCO1, GPI19, ATG26, BRR2, EFT2, PRS4, SET5,

				PRD1, ULP1, RHO3, MAK3, RSC9, KTR3, NOP1, UBR1, MCK1, NFT1, DAL3, ARO4, PHO85, YAL061W, NTG2, RHO5, YGL039W, CPR2, MNT3, YKR070W, TKL2, SWP1, SCEI, PRP28, ACO2, YDR307W, BIO3, SSL2, YPC1, RNH203, IPT1, HAT1, RPO31, ECM32, HEM4, UBA1, EXG2, ERG9, SMB1, PRI2, CDC1, YMR291W, RAD1, MSS116, SHR5, ADE12, IDH2, DIT2, PRP8, URA7, YRF1-7, LPP1, YPD1, GTO1, SNM1, YKL161C, GCD6, YKR104W, LAG1, TAF14, LAS21, PAN2, TAL1, RNT1, DLD2, IMD4, LEU4, AAD4, ERG7, SNT309, KRE6, UBP3, ATG5, IDI1, MOD5, RNR2, HPA3, MTG2, ACF2, ILV2, SER3, ADH7, HSL7, YMR130W, ARO2, PMT2, CDC21, APN1, ERF2, GRX3, PGI1, CYC7, ROT2, GRX5, FRE8, YBR025C, PRO2, YKU80, HPR5, DSF1, TOP3, INO80, YBR284W, SEN54, RNY1, PDB1, HSP31, CDS1, CTL1, RML2, QCR9, SAD1, HMX1, DGA1, YGR043C, SCW4, SKI6, ARO1, PMC1, VPS15, YMR085W, IDH1, SEN1, YHR020W, SAP155, COX17, PHS1, ALD2, TRM2, TGL3, HIS5, PRB1, SEC18, YJR149W, BST1, GIS1, MDM20, MNN9, UBP14, SNZ3, RCE1, HRT3, ADE5,7, CTM1, DSS1, SER1, VMA10, CPR3, GAD1, KCC4, FDH1, ESP1, SOD1, PDX3, PFS2, LPD1, GDA1, SSA2, RRI1, SHM1, OMS1, MXR1, GAS4, OMA1, ADE3, YNK1, YAH1, SNR6, ARD1, TSC13, FET5, LCB4, YNL274C, PTH1, FMP46, LRO1, MET7, YNL168C, KRE5, YNL024C, DUS3, NPR1, ISM1, PDA1, RPT6, SEC59, KAE1, CSH1, PMT6, TSA2, FSH2, MSS1, PRP6, TMT1, YOR059C, PRS3, ICL1, SNU114, GCV2, ARG1, DMA2, YLL056C, URA1, MTG1, ALD3, GET3, NCP1, SNF1, TDP1, DAP2, ADH3, HAS1, SIS2, SSU72, PDR5, GCN20, TRM10, AI4, PDE2, PMT1, GRE3, RPA190, CAR1, DAL2, YPS6, THR1, LEU2, AUS1, ILS1, TRA1, PIM1, MIS1, YPR004C, MNS1, YPT52, GRX4, RPB9, YPR127W, DBF20, ALG7, RPC82, DSE2, PSK2, MNN2, TYR1, KIN2, RHO4, RNR1, PFK2, GAR1, YPS3, HST2, COX15, MST1, CHS3, PTH2, YLR345W, NUP145, ARX1, DDI1, HOS1, TAT2, PPM1, PSD2, UBC13, DCS1, YGL157W, PRR1, NEO1, FMT1, SEC11, ADO1, MHT1, COX9, YSP3, RNR4, HEF3, RHR2, YRF1-6, KEX2, COR1, NPT1, MAG1, SDH4, FBP26, PRX1, ATP3, TAZ1, DLD1, CDC14, RAD57, SEC53, URA8, CDC4, NPL6,

				DBP2, KIN82, FPR4, GCD1, GPI16, SWF1, ERG12, ECM18, NTH1, RPB7, YNL247W, ILV3, OST3, CWH41, PDI1, SPT10, SOL4, URA2, BCS1, POP6, HEM1, GPD1, ELP3, TYS1, PAN5, AAT2, GUT2, LEU9, EST2, EPS1, PKH1, DFG10, PUS6, CDC50, MTF1, ATH1, WBP1, MSF1, YBR242W, YJL213W, CAR2, RSR1, HAM1, UBC8, ASN2, YPR118W, YCL074W, SIW14, CDC5, RSB1, CDC6, DPM1, NIT3, AST2, SIZ1, SLC1, ALD4, GSC2, LSC2, TRF5, AAP1, MKK1, AIR1, DRS2, RHO2, YHR044C, ACO1, PPG1, ARO3, NAM2, DRS1, YFR018C, GPH1, GLT1, FUN12, SMD3, YTA7, YAL049C, MGM1, RNR3, MIH1, RAD50, SCT1, DAL7, ALG9, YPL141C, FMS1, IMP2, PMT3, YOR283W, YOR1, GUK1, PGA3, PPH3, FAS2, HMG2, MEC1, PBN1, SUR2, GUS1, SLT2, DCS2, TUB1, MET10, DPS1, SSD1, CDC10, APT1, HIS2, RPN1, YRF1-1, UBP11, HFD1, MAK10, YGK3, ILV1, SNZ2, DEG1, PFA3, GAL83, MET2, DBP8, ICL2, NMA1, SLM5, PBS2, PPT1, ABP140, ECM38, RAD26, ASH1, RIB5, CDC26, HOM2, DSE4, LIP1, YPS1, RFC3, EFT1, SSA4, DIB1, ESS1, HXK1, UBC1, GPI14, YCK2, ZWF1, MKC7, ENA2, PHR1, REV7, AI2, POS5, MSE1, SKM1, ECI1, ROG1, ULA1, ALG2, GAL1, EHD3, SWD1, DAK2, RDH54, PRO1, DIA4, FRE2, PIB1, NAT2, HEM12, CTF18, AAH1, SIP1, RIB7, ARG5,6, KTR2, UBC6, APA1, STT3, MPD1, SFA1, PTC7, NDE1, ARO7, SDL1, ALG3, FAT1, PRK1, PDH1, YMR226C, HRR25, YPS5, ASP1, HSP82, CDC2, CHL1, DPB2, SMX2, HIS4, AYT1, YHR113W, CPR5, SLX8, SUR4, PFK1, PRE5, SLH1, PXA2, ALG5, DNF2, COX2, CHD1, YPR172W, MSP1, RKM2, SAP4, YBR139W, POP3, FKS1, ARG2, YIL064W, HOM6, GND1, STR3, YDR341C, RAD3, GDH1, PNG1, THR4, YBR014C, RIB3, YFR055W, ALG12, RPA43, YDR541C, AAD3, SWR1, MEF1, XYL2, ARF3, TOM1, SAK1, RPO21, PCM1, DUT1, YMR118C, ALG1, PAN6, PUS1, MRK1, CDC43, PHO12, AAT1, NDE2, PGM2, YMR041C, MCD4, PRP18, FAA2, HYS2, YDR061W, SCH9, PHO8, RPA49, SGA1, CAK1, VHS1, KRS1, HST4, TFG1, OYE2, PFK27, STV1, GLO2, SEN2, YKL071W, LEM3, FPR3, YKL033W-A, PET112, COX1, AAD15, AMD1, YJR107W, TRR2, COQ3, PCA1, ATG1, PSK1, ARH1, SNR19, CCC2, ATG3, ABD1, CDC39,

				YDR415C, RPC40, FRE4, VMA6, HPT1, VAS1, GAS2, RPA14, ISY1, SNO1, COX12, GPX1, MSM1, UBC9, URA6, DTD1, YKT6, YNL134C, ADE8, TAD3, LYS2, TOR1, COX7, SMX3, LYS12, ADH2, PPZ1, TSC10, SHM2, PTP2, NUS1, DIM1, CTK2, APC1, HOR2, YDL124W, YOR246C, YJL045W, GTO3, ATG7, NPY1, TPP1, INM1, ARG3, BDH1, GLY1, GPI12, MRM1, TPA1, PCS60, KTR4, SUC2, THS1, LIP5, FRS2, KTR6, GAL3
oxidoreductase activity	156 out of 1943 genes, 8.0%	335 out of 6348 genes, 5.3%	1.51e-07	ADH3, AI2, ERG11, RFS1, BNA4, YAL061W, YIR035C, AI4, AST1, GRE3, YGL039W, FRE2, LEU2, YPR004C, MIS1, GRX4, RIB7, ARG5,6, YPR127W, MPD1, SFA1, NDE1, TYR1, RNR1, YMR226C, TYW1, QCR8, ERG9, COX15, LYS1, SMM1, HIS4, IDH2, CCP1, DIT2, YGR207C, COX2, YPR172W, YGL157W, ERG5, COX9, RNR4, DLD2, IMD4, UGA2, HOM6, COR1, AAD4, GND1, SDH4, GDH1, PRX1, YBR014C, DLD1, RNR2, GAL80, YDR541C, AAD3, XYL2, SER3, ADH7, ARO2, NDI1, GRX3, PDI1, CYC7, YMR118C, GRX5, FRE8, PRO2, GPD1, DSF1, GUT2, PAN5, NDE2, PDB1, DFG10, YMR041C, HMX1, QCR9, ERG3, AAD6, ARO1, OYE2, IMD2, IDH1, YKL071W, COX17, ALD2, AI5_BETA, PCK1, AST2, HEM13, ALD4, COX1, YJR149W, AAD15, GIS1, MDH2, CBR1, ADH4, ERV2, TRR2, FDH1, SOD1, GLT1, ARH1, PDX3, LPD1, RNR3, YHB1, IDP1, FMS1, MXR1, PGA3, HMG2, FAS2, FRE4, FRE3, ADE3, YAH1, SUR2, TSC13, FET5, YNL274C, MET10, COX12, ILV5, GPX1, FMP46, HFD1, YNL134C, DUS3, LYS2, COX7, LYS12, PDA1, ADH2, ZTA1, TSC10, SER33, RLI1, TSA2, YDL124W, YOR246C, YJL045W, HOM2, BDH1, ERG25, GCV2, SCO1, TPA1, URA1, ZWF1, ALD3, NCP1
oxidoreductase activity, acting on CH-OH group of donors	47 out of 1943 genes, 2.4%	85 out of 6348 genes, 1.3%	0.00119	YMR041C, YGL157W, ADH3, AAD6, YNL274C, ILV5, YAL061W, ARO1, DLD2, GRE3, IMD4, YGL039W, YNL134C, IMD2, HOM6, IDH1, AAD4, GND1, LEU2, LYS12, ADH2, TSC10, RIB7, DLD1, YPR127W, AAD15, SER33, MDH2, SFA1, YDR541C, AAD3, YDL124W, ADH4, XYL2, ADH7, SER3, FDH1, BDH1, HIS4, IDH2, IDP1, GPD1, ZWF1, PAN5, GUT2, FAS2, HMG2
oxidoreductase activity, acting on	44 out of 1943	78 out of 6348	0.00123	YMR041C, YGL157W, ADH3, AAD6, YNL274C, ILV5, YAL061W, ARO1, GRE3,

the CH-OH group of donors, NAD or NADP as acceptor	genes, 2.3%	genes, 1.2%		IMD4, YGL039W, YNL134C, IMD2, HOM6, IDH1, AAD4, GND1, LEU2, LYS12, ADH2, TSC10, RIB7, YPR127W, AAD15, SER33, MDH2, SFA1, YDR541C, AAD3, YDL124W, ADH4, XYL2, ADH7, SER3, FDH1, BDH1, HIS4, IDH2, IDP1, GPD1, ZWF1, PAN5, FAS2, HMG2
transporter activity	175 out of 1943 genes, 9.0%	435 out of 6348 genes, 6.9%	0.00446	MTM1, CRC1, YCF1, PDR5, PSE1, NFT1, YRO2, HOL1, ITR1, SSU1, AUS1, DNF1, YKE4, USO1, ZRC1, HXT5, YDL119C, MRH1, TPC1, ENB1, HXT11, ARN1, NHA1, FLC1, FAT1, OPT1, ODC1, SNF3, QCR8, KAP95, AGP1, ITR2, SEC14, HUT1, FPS1, PXA2, TAT2, TPO3, DNF2, COX2, YOR271C, YPR011C, ATO3, NEO1, RIM2, COX9, YKR104W, SEC28, KAP122, ACB1, SLY41, COR1, ALP1, ATP8, AVT1, TOM20, ATP3, AVT7, SEC61, HXT16, YOR071C, TOM71, ATP2, HXT9, KAP120, SGE1, MUP1, AVT4, CAN1, YMC2, YVC1, DAL5, MDL1, YIL171W, KAP123, ENA5, TOM6, PHO84, COP1, TAT1, CDC50, QCR9, NMD5, OAC1, ENA1, PMC1, FCY22, AZR1, AQY2, MID1, CLC1, YIA6, STV1, LST7, SXM1, ADY2, LEM3, RSB1, YOL163W, YCR023C, RET2, COX1, APL3, DRS2, GAP1, BAP3, PTR2, VMA10, ZRG17, TIM9, TOM70, SUL1, PCA1, DTR1, QDR2, SMF1, VHT1, CCC2, TPO2, DAL4, VBA1, PHO89, YPR003C, AVT2, JEN1, HXT15, YOR1, LST4, TOM40, ODC2, COT1, SEC66, VPS73, VRG4, MCH1, VMA6, YOR378W, COX12, BAP2, RGT2, MDJ2, YIL166C, MCH5, MEP2, GNP1, COX7, YLL053C, FUI1, HXT13, FUR4, HXT12, MSN5, CTP1, HXT2, YEA6, SIT1, SEC21, SEC27, CTR3, YLR004C, FEN2, FCY2, ARN2, PIC2, VCX1, GAL2, YDR338C, DIC1, COX18, MAL11, YFL054C, AAC3, GET3, ENA2, SAM3
mannosyltransferase activity	27 out of 1943 genes, 1.4%	44 out of 6348 genes, 0.7%	0.01594	MNT2, KTR3, PBN1, KTR1, MNN1, MNN2, ALG3, PMT5, ALG2, PMT1, PMT2, MNT3, ALG1, GPI18, KTR7, KTR4, ALG9, YDR307W, DPM1, PMT3, GPI14, KTR2, MNN9, KTR6, CSH1, PMT6, ALG12
cofactor binding	72 out of 1943 genes, 3.7%	156 out of 6348 genes, 2.5%	0.01818	RFS1, CAR2, HST4, DPL1, YGL039W, AGX1, OYE2, FRE2, YPR004C, MTO1, BIO3, RIB7, HIS5, ARG5,6, NDE1, SDL1, SER1, TRR2, GAD1, FDH1, TYW1, GPH1, GLT1, HST2, PDX3, ARG8, LPD1, SMM1, HIS4, SHM1, HMG2, FRE3, FRE4, YPR172W, YGL157W, YNL274C, ILV5, TRP5, ACB1, DLD2, ILV1,

				DUS3, GND1, STR3, LYS2, PPZ1, SDH4, THR4, SHM2, DLD1, YFR055W, SER33, YDR541C, YJL045W, XYL2, SER3, ILV2, HOM2, NDI1, GLY1, YMR118C, YLL056C, FRE8, PRO2, HEM1, DSF1, GPD1, AAT2, PAN5, AAT1, NCP1, NDE2
binding	1033 out of 1943 genes, 53.2%	3138 out of 6348 genes, 49.4%	0.03048	SNX3, TR(UCU)E, TUF1, GCN4, LOS1, IPP1, YIR035C, AGX1, HIT1, DNF1, STP3, ABF2, CMP2, AGA1, GCD10, MTO1, TAH1, YDL119C, PUS4, CLU1, YFR032C, DMA1, IRS4, YPT32, TYW1, NTO1, TOS3, TK(CUU)J, MET14, TG(UCC)N, LYS1, TS(AGA)D2, CYM1, CDC42, CCP1, NAT1, YLR419W, SSC1, HSP78, FAB1, TRM3, CYR1, ATG11, DAP1, NET1, LSG1, YPL236C, YTA6, GAL80, GCD7, CMK1, TE(UUC)J, ATP2, SGS1, CKS1, SUV3, YDR161W, HOM3, MSW1, TIF4632, ENA5, TQ(UUG)D3, SAR1, CCL1, CAD1, RET1, YNL045W, ENA1, VHR1, GLO1, IMD2, SNF7, YIA6, SNR51, SPT20, SUP35, SWI6, RAS2, HSP104, PCK1, AI5_BETA, TG(GCC)B, TR(ACG)K, PYK2, MDH2, FAA4, LST8, SGT2, ARC15, TFB3, REF2, NDD1, TIM9, TOM70, URE2, TI(AAU)L1, ARG8, ZAP1, TH(GUG)M, YHB1, FOB1, IDP1, INO1, STE12, MRPL3, CSL4, HAT2, ODC2, COT1, FLO1, FRE3, RAD16, LHS1, RMI1, TRP5, ILV5, YTH1, NAB2, RLM1, DIN7, KAP104, RLI1, CDC7, MSN5, PRP5, MST27, SNR11, KTR1, YIL151C, DUR1,2, CTR3, ERG25, ATP11, RFM1, DIC1, HTB1, PRS4, RHO3, YER130C, RSC9, IVY1, PUB1, UBR1, YOR387C, YAL061W, ISA1, RHO5, YGL039W, RTT106, CPR2, SWI4, VTH2, SMC6, TD(GUC)J1, TG(CCC)D, BUL1, MYO1, BIO3, MOT3, MTR2, MFA2, ECM32, EXG2, GOS1, ERG9, SMB1, YMR291W, HCR1, HEK2, MFT1, RPS9B, ADE12, DIT2, TS(AGA)B, YPR011C, PEX12, PAN2, TA(UGC)L, TR(UCU)B, SNT309, TQ(UUG)D1, RNR2, FLO5, HTZ1, ILV2, SER3, SSP120, ADH7, TS(AGA)A, ERF2, FRE8, PRO2, YKU80, HPR5, DSF1, SAC3, HSP31, SAD1, GCN1, TAF13, ARO1, YMR085W, TS(AGA)L, IDH1, SKI3, SEN1, SXM1, YHR020W, GCR1, SNR56, COX17, ERG28, TGL3, PRB1, SEC18, TE(CUC)D, ADE5,7, DSS1, SER1, GIP3, SSE2, YOL054W, GAD1, ESP1, PRP42, NIP1, PDX3, YHC1, SLM2, BRF1, SSA2, RRI1, SHM1, BUD20, BUR6, TOM40, YAH1, SNR6, LCB4, MIF2, HSE1, MDJ2, NOP53, TT(AGU)J,

				MAL23, CCT2, TM(CAU)J1, RPS0A, YLR278C, KAE1, SEC21, PRP6, ERJ5, TL(UAA)J, ARG1, DMA2, YLL056C, CNE1, AFT1, GET3, NCP1, SNF1, STE50, SSU72, PDR5, TAP42, LEU2, ILS1, TRA1, YPR004C, ARP2, USO1, NRG2, RPB9, RPC82, TPC1, UTP13, PSK2, UGA3, TOS4, PEX22, GAR1, HST2, MST1, CHS3, NOP14, KRE33, TQ(UUG)D2, PDR8, ORC5, PDS1, YGL157W, NEO1, OSH6, MHT1, CTF13, HAC1, RNR4, KAP122, HEF3, YRF1-6, AFG1, KEX2, COR1, NSG1, MSH5, YDL203C, TS(AGA)E, SDH4, FBP26, ATP3, SEY1, DLD1, ZIP1, LOC1, ENT1, DBP2, KIN82, KAP120, ERG12, ILV3, YER184C, YBR062C, PHD1, YMC2, SPT8, CUP1-2, URA2, BCS1, GPD1, HEM1, TYS1, EST2, PUS6, MBF1, MSF1, TE(UUC)L, NSG2, CAR2, RSR1, CLC1, YCL074W, VPS20, CDC5, PCL1, NAB6, SNR61, MKK1, AIR1, DRS2, ACO1, PPG1, DRS1, DAL81, GPH1, GLT1, CUS2, FUN12, TIF5, MCM16, RNR3, RAD50, YPL141C, YOR1, JSN1, HMG2, MEC1, SPH1, GUS1, SLT2, SSD1, CDC10, RPN1, YRF1-1, SUP45, YGK3, TR(UCU)J1, CUP9, DBP8, TC(GCA)P1, HPR1, PBS2, PPT1, MET18, MBA1, MF(ALPHA)1, SCP160, RAD26, KAR1, HSP60, SSA4, VTH1, SWI5, AAC3, TM(CAU)J3, ENA2, PHR1, MSE1, GAL1, DAK2, RDH54, DIA4, PIB1, ZRC1, PES4, RIB7, ARG5,6, RPS24B, APA1, SFA1, DOA1, PTC7, NDE1, TQ(UUG)B, SDL1, TE(UUC)C, NHA1, PDR3, PDH1, YMR226C, HRR25, HSP82, CHL1, YPL230W, SMX2, ATG19, CPR5, SLH1, GAT1, CHD1, YPR172W, RPL16B, SEF1, RIM2, RAD3, GDH1, PNG1, BOI1, VPS24, MEF1, XYL2, TOM1, SAK1, RPO21, PCM1, GIM3, YMR118C, YBR033W, PUS1, MRK1, CDC43, PGM2, GBP2, FAA2, ISU2, IXR1, HYS2, YDR061W, PHO8, RPA49, CAK1, KRS1, MID1, TFG1, ATG18, RPS0B, GLO2, SEN2, YKL071W, SEC17, GAT2, BUD21, COX1, STP4, TRR2, MET28, ATG17, ATG1, PCA1, SWA2, PSK1, SIP18, SNR19, CCC2, RPL6B, AIP1, BNR1, YBL054W, TR(UCU)K, YOX1, RPC40, HPT1, VAS1, MSM1, TH(GUG)E1, YKT6, YNL134C, TAD3, LYS2, SMX3, LYS12, TN(GUU)K, PPZ1, DOT6, APC1, YEA6, LSM3, INO4, YJL045W, ATG7, CDC31, TG(GCC)M, INM1, CCT4, MLP1, TR(CCG)L, GLY1, TP(AGG)C, TPA1, STU1, ORC4, GAL3, WRS1,

				HOF1, CRN1, YCF1, ERG11, CRC1, IST3, YPT1, RPG1, RPT3, RFS1, TOS8, MSH2, MAL33, AST1, DPL1, RPL5, YKE4, TPK2, MSD1, ENB1, TLG2, TS(AGA)J, DED1, ARN1, YFR006W, MET31, TG(GCC)C, SNF3, SME1, TEL2, SMM1, PUS7, YNR071C, GLO4, POL5, NHP10, TR(UCU)J2, SAM4, SKI2, ERG5, PAC10, SSL1, TT(UGU)P, TG(UCC)O, STB5, ADE6, KAR2, SUT1, SEC61, YIP5, UTP5, HMS1, ALG14, ARG81, SEC24, PRS2, NDI1, DAL80, JEM1, VAM7, BGL2, SPA2, RPS24A, MDL1, KAP123, PEX10, GSH2, ERG3, NMD5, HOG1, RPA135, LSM4, CFT1, YRF1-3, RGT1, CCA1, CWC23, RET2, EDC2, SIR1, ADH4, IRE1, FSP2, IZH1, FIT1, CTI6, KIP1, UPC2, HNT1, FAA3, RPT4, LSM5, LEU1, TKL1, MST28, RGT2, DAD4, CCE1, TQ(UUG)E1, AXL1, DBF4, ZTA1, STE7, PPA2, YRF1-2, PEP7, TEL1, RSC30, SER33, PET122, ENT5, DER1, DBP5, TUB3, PAC2, YLR247C, TR(UCU)M2, PIC2, PGS1, SCO1, BRR2, CHS6, EFT2, TMA20, PRD1, TG(GCC)P2, TR(ACG)D, IOC4, FIP1, NOP1, MCK1, NFT1, YGR250C, SNX41, PHO85, NTG2, TKL2, CCT6, TE(UUC)P, PRP28, ACO2, YKR096W, RRP5, SSL2, TS(AGA)M, RPO31, HAT1, ODC1, YRM1, UBA1, CDC1, PRI2, KAP95, RAD1, MSS116, MF(ALPHA)2, TV(CAC)D, IDH2, HMRA1, SEC15, PRP8, YNR063W, YRF1-7, RPS27B, YOR271C, NOP13, TA(AGC)K2, SNM1, YKL161C, TA(UGC)A, GCD6, PEX13, CBC2, RNT1, DLD2, IMD4, CRP1, UTP15, IDI1, STO1, MOD5, VMA22, GIP4, SHC1, MTG2, ORC2, GCS1, HAL9, BSP1, APN1, CYC7, YBR025C, TOP3, INO80, RNY1, SPT3, RML2, HMX1, HHT1, SKI6, PMC1, VPS15, ECM7, HIS5, APL3, GIS1, UBP14, VPS25, SNZ3, YAP3, CPR3, KCC4, FDH1, SOD1, PFS2, SPT4, LPD1, HSP26, SEC23, VPS52, OSH3, GAS4, CIN5, TS(AGA)D3, OMA1, YDR520C, SWH1, SEC9, ADE3, YNK1, FET5, YNL274C, PEA2, THP1, MET7, YNL168C, SUI3, SUA7, BOI2, NPR1, DUS3, ISM1, RPT6, SLF1, CTP1, MSS1, GIS2, PRS3, SNU114, VCX1, WTM2, MTG1, SUI1, ADH3, HAS1, MTM1, PSE1, YCR087C-A, GCN20, GAL4, AI4, CAR1, RPA190, AUS1, THR1, MIS1, PIM1, RPS29A, MNS1, YPT52, GIM4, VAM3, YJL103C, MFA1, DBF20, AGA2, TYR1, TV(UAC)B, RHO4, KIN2, RNR1, PFK2, HHT2, YPR022C, THP2,

				YLR345W, RBG2, MOB1, NUP145, DDI1, ARX1, PDS5, PRR1, YSP3, YOR262W, FLO10, MAG1, PUF2, ADY3, RAD57, HYP2, TQ(UUG)C, TN(GUU)C, SGN1, FPR4, GCD1, HSP10, SWF1, YNL247W, RPB7, NTH1, UTP22, FZF1, COX20, YRR1, SPT10, AAT2, PAN5, TG(GCC)J2, ELP3, SHY1, EPS1, TG(GCC)O2, PKH1, MTF1, ATH1, PEP12, TE(UUC)E1, SSF2, CCT5, DLS1, YLL054C, RPI1, CDC6, SIZ1, AST2, RAD59, TE(UUC)M, LSC2, SNR58, AAP1, RHO2, TR(UCU)D, NOG1, NAM2, RPF1, GFD2, SMD3, RAD34, SMF1, YTA7, MGM1, RPL24B, BRR1, TS(AGA)D1, FLO9, SFB3, GUK1, PPH3, FAS2, TUB1, TE(UUC)B, DPS1, TS(UGA)P, APT1, SFB2, VAC8, SWI1, ILV1, BFR1, SNZ2, PFA3, DEG1, MIG3, RME1, SLM5, AZF1, SIT1, ABP140, YDJ1, ASH1, ACA1, HOM2, CDC26, AXL2, CNS1, RFC3, EFT1, TG(CCC)O, HXK1, DCN1, YCK2, HAP2, ZWF1, PRT1, AI2, TR(UCU)M1, POS5, SKM1, ULA1, YLR281C, HSP42, ESF1, PRO1, SSO2, FRE2, RRF1, CTF18, CSE4, TQ(UUG)E2, RTS2, PRK1, TA(UGC)O, AHA1, CDC2, GTR2, DPB2, HIS4, YHR113W, VPS74, NRD1, SLX8, XBP1, PFK1, USE1, PXA2, YNR029C, CUP1-1, TH(GUG)K, DNF2, FAR1, COX2, TAF6, MSP1, ACB1, TIF6, HOM6, STR3, GND1, YDR341C, EMG1, THR4, RIB3, YBR014C, YFR055W, TOM71, YDR541C, IRR1, TL(GAG)G, CAT8, SWR1, HAP1, ARF3, NRG1, ENT4, TCM62, YVC1, KOG1, AAT1, NDE2, TS(UGA)E, HHO1, SCH9, OAC1, RET3, VHS1, HST4, PFK27, OYE2, RDS1, ECM29, YCS4, RPN4, MRL1, TQ(UUG)L, ABD1, YML081W, MSH4, MUB1, YDR415C, NMD2, FRE4, RFA2, HCH1, URA6, TOR1, RRP9, ADH2, SHM2, TSC10, DIM1, CAP2, SRN2, YOR246C, UTP20, RAP1, NPY1, YFH1, ARG3, ARN2, TE(UUC)K, BDH1, MRS1, MRM1, ARG80, PCS60, THS1, LIP5, SRP72, FRS2
substrate-specific transporter activity	144 out of 1943 genes, 7.4%	359 out of 6348 genes, 5.7%	0.03632	YCF1, PSE1, YRO2, HOL1, ITR1, SSU1, DNF1, YKE4, USO1, ZRC1, HXT5, MRH1, TPC1, ENB1, HXT11, ARN1, NHA1, FLC1, FAT1, OPT1, ODC1, SNF3, QCR8, KAP95, AGP1, ITR2, SEC14, HUT1, FPS1, TAT2, DNF2, COX2, YOR271C, ATO3, NEO1, RIM2, COX9, SEC28, KAP122, ACB1, COR1, ALP1, ATP8, AVT1, TOM20, ATP3, SEC61, HXT16, YOR071C, TOM71, ATP2, HXT9, KAP120,

				MUP1, AVT4, CAN1, YMC2, YVC1, DAL5, MDL1, YIL171W, KAP123, ENA5, TOM6, PHO84, COP1, TAT1, CDC50, QCR9, NMD5, OAC1, ENA1, PMC1, FCY22, AQY2, MID1, CLC1, AZR1, YIA6, STV1, LST7, SXM1, ADY2, LEM3, RSB1, YCR023C, RET2, COX1, APL3, GAP1, DRS2, BAP3, PTR2, VMA10, ZRG17, TOM70, TIM9, PCA1, SUL1, SMF1, CCC2, DAL4, VBA1, PHO89, YPR003C, JEN1, HXT15, LST4, ODC2, TOM40, COT1, SEC66, VRG4, VPS73, VMA6, COX12, BAP2, RGT2, MDJ2, MEP2, GNP1, COX7, FUI1, HXT13, FUR4, HXT12, MSN5, HXT2, SIT1, SEC21, SEC27, CTR3, FCY2, ARN2, PIC2, VCX1, GAL2, DIC1, COX18, YFL054C, MAL11, GET3, ENA2, SAM3
transferase activity, transferring hexosyl groups	43 out of 1943 genes, 2.2%	84 out of 6348 genes, 1.3%	0.04136	KTR3, PBN1, ALG6, MNN1, PMT5, ALG2, WBP1, FKS1, PMT1, KRE5, TPS3, MNT3, GLG1, SWP1, GPI18, KTR7, YDR307W, DPM1, KTR2, GSC2, MNN9, CSH1, STT3, PMT6, ALG12, MNT2, ALG14, KTR1, MNN2, ALG3, GPH1, PMT2, OST3, CHS3, ALG1, GPI19, ALG9, ATG26, KTR4, PMT3, GPI14, KTR6, ALG5
